# Validation of clinical acceptability of deep-learning-based automated segmentation of organs-at-risk for head-and-neck radiotherapy treatment planning

**DOI:** 10.3389/fonc.2023.1137803

**Published:** 2023-04-06

**Authors:** J. John Lucido, Todd A. DeWees, Todd R. Leavitt, Aman Anand, Chris J. Beltran, Mark D. Brooke, Justine R. Buroker, Robert L. Foote, Olivia R. Foss, Angela M. Gleason, Teresa L. Hodge, Cían O. Hughes, Ashley E. Hunzeker, Nadia N. Laack, Tamra K. Lenz, Michelle Livne, Megumi Morigami, Douglas J. Moseley, Lisa M. Undahl, Yojan Patel, Erik J. Tryggestad, Megan Z. Walker, Alexei Zverovitch, Samir H. Patel

**Affiliations:** ^1^ Department of Radiation Oncology, Mayo Clinic, Rochester, MN, United States; ^2^ Department of Health Sciences Research, Mayo Clinic, Phoenix, AZ, United States; ^3^ Department of Radiation Oncology, Mayo Clinic, Phoenix, AZ, United States; ^4^ Department of Radiation Oncology, Mayo Clinic, Jacksonville, FL, United States; ^5^ Google Health, Mountain View, CA, United States; ^6^ Research Services, Comprehensive Cancer Center, Mayo Clinic, Rochester, MN, United States; ^7^ Robert D. and Patricia E. Kern Center for the Science of Health Care Delivery, Mayo Clinic, Rochester, MN, United States

**Keywords:** deep learning, autosegmentation, head and neck cancer, radiation therapy, clinical validation, comprehensive, organs-at-risk

## Abstract

**Introduction:**

Organ-at-risk segmentation for head and neck cancer radiation therapy is a complex and time-consuming process (requiring up to 42 individual structure, and may delay start of treatment or even limit access to function-preserving care. Feasibility of using a deep learning (DL) based autosegmentation model to reduce contouring time without compromising contour accuracy is assessed through a blinded randomized trial of radiation oncologists (ROs) using retrospective, de-identified patient data.

**Methods:**

Two head and neck expert ROs used dedicated time to create gold standard (GS) contours on computed tomography (CT) images. 445 CTs were used to train a custom 3D U-Net DL model covering 42 organs-at-risk, with an additional 20 CTs were held out for the randomized trial. For each held-out patient dataset, one of the eight participant ROs was randomly allocated to review and revise the contours produced by the DL model, while another reviewed contours produced by a medical dosimetry assistant (MDA), both blinded to their origin. Time required for MDAs and ROs to contour was recorded, and the unrevised DL contours, as well as the RO-revised contours by the MDAs and DL model were compared to the GS for that patient.

**Results:**

Mean time for initial MDA contouring was 2.3 hours (range 1.6-3.8 hours) and RO-revision took 1.1 hours (range, 0.4-4.4 hours), compared to 0.7 hours (range 0.1-2.0 hours) for the RO-revisions to DL contours. Total time reduced by 76% (95%-Confidence Interval: 65%-88%) and RO-revision time reduced by 35% (95%-CI,-39%-91%). All geometric and dosimetric metrics computed, agreement with GS was equivalent or significantly greater (p<0.05) for RO-revised DL contours compared to the RO-revised MDA contours, including volumetric Dice similarity coefficient (VDSC), surface DSC, added path length, and the 95%-Hausdorff distance. 32 OARs (76%) had mean VDSC greater than 0.8 for the RO-revised DL contours, compared to 20 (48%) for RO-revised MDA contours, and 34 (81%) for the unrevised DL OARs.

**Conclusion:**

DL autosegmentation demonstrated significant time-savings for organ-at-risk contouring while improving agreement with the institutional GS, indicating comparable accuracy of DL model. Integration into the clinical practice with a prospective evaluation is currently underway.

## 1 Introduction

Head and Neck (HN) cancer is a significant burden on global health, accounting for an estimated5% of world-wide cancer-related mortality in 2020 (1) – similar in magnitude to breast and pancreas cancers – and it is expected that over 700,000 people will die from HN cancer in 2030 (an increase of 38% from 2016) (2). Unfortunately, this burden is shouldered primarily by low and middle income countries lacking adequate capacity and access to radiation therapy (RT), chemotherapy, and surgery (2). RT plays a critical role in the management of HN cancer: it is indicated in an estimated 74% of HN cancer patients per published guidelines and evidence (3).

Delivering function-preserving, curative HN-RT is challenging due to the complex anatomy and the need to balance the competing objectives of delivering adequate radiation dose to the tumor while sparing adjacent organs-at-risk (OARs). A custom RT treatment plan needs to be designed that finds the optimal balance for an individual patient, and the quality of the treatment plan plays an important role in improving clinical outcomes (4). Furthermore, improved survival has been associated with RT provided by high-volume radiation oncologists (ROs) (5). Integral to this process is the accurate segmentation of the OARs, as radiation injury to OARs can lead to a significant detriment in function and quality of life (6), as seen by the high incidence of suicide in patients with HNC (7, 8). This segmentation must also be comprehensive to mitigate the wide range of potential severe adverse effects, ranging from dysphagia and xerostomia to neuropathy and necrosis. Managing these risks requires segmentation not only of the swallowing structures, mandible, mastoid, and salivary glands, but also neurological organs (brachial plexus, brainstem, cord, optic nerves, optic chiasm, and brain), auditory structures (external auditory canal, and cochlea), and optical structures (eye, lens, lacrimal gland, and retina). It has also been demonstrated that risk of stroke (9) and general cerebrovascular events (10, 11) is associated with RT for HN cancer patients, motivating the need for delineation of carotid arteries (CAs).

High-quality RT for HN cancer patients requires accurate and comprehensive OAR segmentation. Our institutional guidelines define 42 OAR structures that may be contoured for HN cancer patients (see **Table S1** in the **Supplemental materials**). While the specific OAR structures required for treatment planning for each patient varies depending on the site, extent, and staging of the disease, each of these structures has situations in which it is necessary to include it. In addition, having a comprehensive set of contours included in the patient’s data set simplifies the collection of dose-volume histogram (DVH) data for outcomes analysis. HN anatomy is complex, and manual segmentation of them is particularly time-consuming and requires significant investment in personnel resources (12). Furthermore, heterogeneity in the quality of manually segmented structures has been widely reported (13–15). Ultimately, the requirements for manual contouring of OARs can be a barrier to patient access for intensity-modulated RT for HN cancer, particularly in low and medium resource environments.

There is great interest in expanding indirect access to high-quality RT *via* autosegmentation tools using deep learning (DL) models informed by expert-level contouring experience (16). These autosegmentation tools have the potential to produce efficiency gains and standardization in the treatment planning process (16). Consequently, there has been much interest in pursuing these models (17–19), but to date there has not been widespread clinical adoption. One limitation is that none of the reported models provides a comprehensive set of all recommended OARs for HN cancer (20). For instance, the brachial plexus (BP) is often not included in the model, despite having both an important role in treatment planning and generally requiring substantial time to contour (21). In addition, the segmentations produced by these models generally require substantial manual edits of multiple OARs in order to be accurate enough for treatment planning.

One common challenge for deep learning (DL) tools in autosegmentation for RT is the absence of training and validation datasets of sufficient size, consistency, and quality (16). At our institution, all OAR segmentation is governed by a detailed set of institutional standards, primarily based on international consensus guidelines (20). Using these standards, two of the authors (both HN-expert ROs) were given protected time away from clinical responsibilities to contour on retrospectively-collected patient datasets, and without the time constraints experienced during daily clinical practice, spending an average of more than 11 hours per patient dataset (21) (exceeding the typical amount of time available for a clinical case). This effort resulted in a consistent “gold standard” (GS) dataset that best reflects the international consensus and institutional standards for 490 retrospectively-identified patients (21). Using this foundation, a 3D U-Net convolutional neural network (19) was trained using the planning computed tomography (CT) images and curated set of 42 OARs from 445 of these patients.

The standard contouring workflow using humans only is time intensive. We hypothesize that a DL-assisted workflow could significantly reduce contouring time compared to a fully manual workflow. Here, we report the results of a randomized, single-blind observational study comparing the OAR contouring workflow with and without the use of our DL autosegmentation for a hold out (HO) cohort of 20 patients’ data available from the GS that had not been previously used for model training, testing, or validation. The study assessed the feasibility of integrating the model into clinical practice and readiness for external validation by measuring the potential time-savings, geometric agreement with the institutional gold standard contours for each patient, and dosimetric impact.

## 2 Materials and methods

### 2.1 OAR contouring workflow

Our department has developed a comprehensive set of OAR contouring guidelines that is used for all campuses. The HN OAR guidelines were primarily developed by three of the authors of this study, two HN-expert ROs (SHP and RLF) and a senior certified medical dosimetrist (AEH). It was based on international consensus guidelines (20) and standardized nomenclature (22). Training was provided to all staff involved in contouring after the adoption of the guidelines, and a detailed electronic document was distributed as a reference.

The contouring workflow at our institution starts with initial contouring performed on a patient’s planning CT (pCT) by a member of the dosimetry staff: either a CMD or medical dosimetry assistant (MDA). The MDA’s role is data preparation for treatment planning, with a large focus on OAR contouring (for which they receive extensive training). The RO reviews and revises the OAR contours and adds the target volumes. These final contours are then used for treatment plan design.

### 2.2 Selection of patient data sets

This study was conducted at two campuses of Mayo Clinic, a National Cancer Institute-designated comprehensive cancer center within an academic medical center in the United States, with data collected under approval by the Mayo Clinic institutional review board. Retrospective chart data including CTs for adult patients (age > 18 years) receiving HN-RT between January 1, 2016 and October 1, 2020 were collected. Patients were included if they had a pCT for external beam RT (either with x-rays or protons) that was acquired according to the departmental standard protocol with 2 mm slice thickness. Patient data was excluded if the patient was not in a thermoplastic mask, if the pCT contained a proton-specific range-shifting device, or if a small field-of-view reconstruction of the planning CT was not available. Patient data were not automatically excluded on the basis of previous surgery and/or RT; however, each of those cases was reviewed by an author who was a HN-expert RO (RLF or SHP) prior to inclusion to ensure that the anatomical changes associated with the previous treatment still allowed for identification of the majority of OARs of interest. Typical voxel size was 1.27 x 1.27 x 2 mm^3^. A total of 490 patient data sets were collected and anonymized for model development and evaluation.

### 2.3 Curation of gold standard data sets

**Figure 1:**
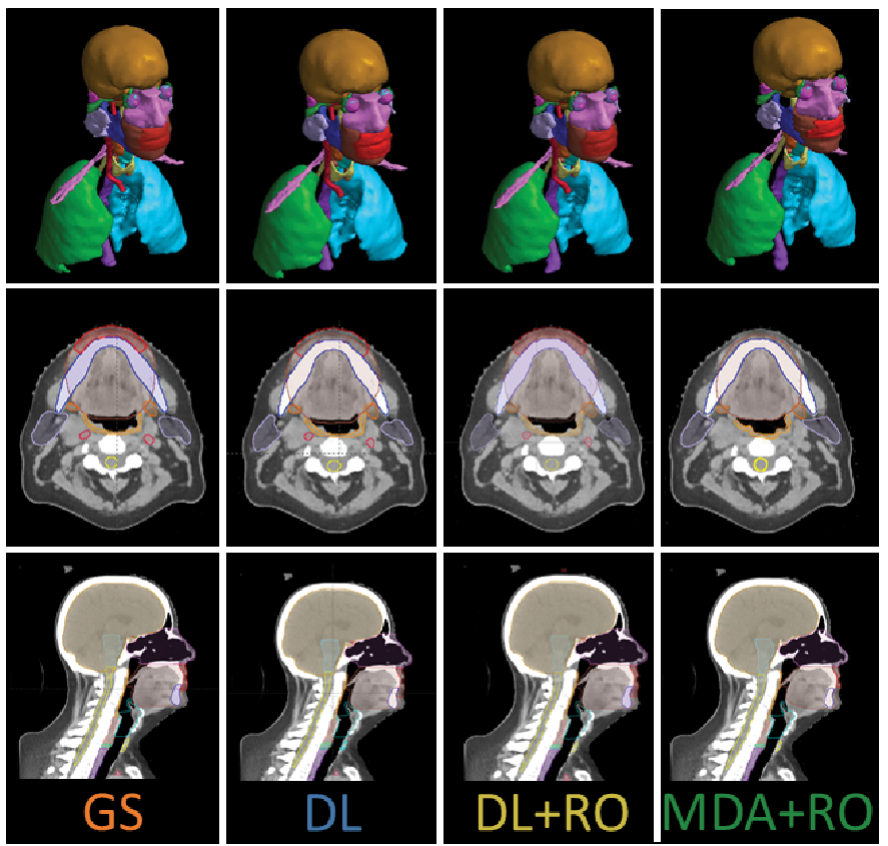
3D visualization, and an axial and sagittal slice of the CT with the contours from the gold standard (GS) dataset, as well as the DL, DL+RO, and MDA+RO contours for a representative patient.

The same two HN-expert RO-authors (referred to as the RO-As below) who developed the institutional contouring guidelines were given time away from clinical duties to create a set of OAR contours for each of the 490 collected patient data sets, assisted by members of the dosimetry team. The dosimetry team was composed of certified medical dosimetrists and MDAs. The contouring staff had access to the pCT, a small field-of-view reconstruction, and – if available – a contrast-enhanced CT, but not the contours used for the patient’s treatment. Due to the protected time, and retrospective nature of the curation, the contouring team was able to spend a mean of 11.6 hours per case during curation. The data curation process and infrastructure is discussed elsewhere (21). The 3D representation and select CT slices with contours for the GS are shown in **Figures 1** for a sample patient, with additional representative slices is given in **Figure S1** of the **Supplemental material** (**Figure S1**).

### 2.4 Model architecture and training

**Figure 2:**
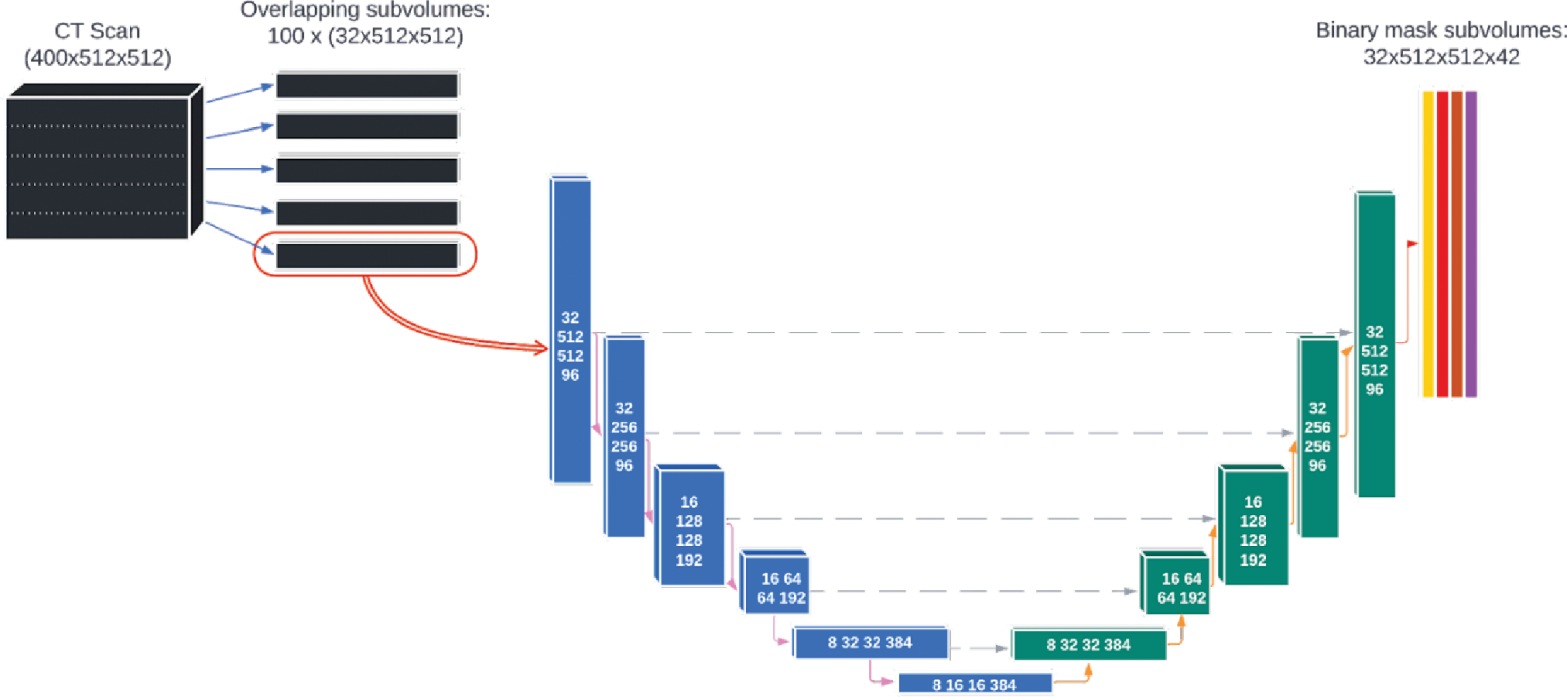
The architecture of the deep learning model used for autosegmentation of organ-at-risk for head and neck cancer radiation therapy.

The deep learning model was based on a single, custom 3D U-Net architecture (**Figure 2**). This architecture has been shown to be well-adapted to the complexity of medical image segmentation and has been shown to perform very well compared to other architectures and approaches (19, 23, 24). The model was adapted from the model used by Nikolov, et al. (19) and is fully 3D: it operates on 32x512x512 voxel sub-volumes of a CT. This is the largest sub-volumes used by any previously reported 3D autosegmentation model (19, 25–62). The model consists of 6 convolution blocks each for the encoding and decoding directions, and outputs 42 binary OAR labels for each input voxel (discussed in details in section 2 of the **Supplemental material**). The number of slices used in each subvolume and the number of convolution blocks was chosen because the authors felt it would achieve a satisfactory balance between providing the model with enough depth to appropriately handle the complexity of the contouring task while also being computationally tractable for model training and inference.

The model was trained on TPUv3 with spatial partitioning using a hybrid loss function consisting of a region-based Dice loss and a voxel-wise focal loss to account for the large variability in OAR sizes. This model was initially re-trained using 544 retrospectively identified patient datasets that were not included in the GS. Training and inference were done using only the GS contours and the planning CT, without access to any additional image series or reconstructions. Some patients were missing structures due to previous surgical excision. In such cases, the DL model was simply presented with an empty contour for the corresponding structure and not given any additional guidance or patient metadata. The empty contours served as negative examples during model training. The model’s ability to omit the missing structures was then assessed by computing a contingency table for the presence of the contour compared to the presence in the GS structure set. 312 CTs were used for training, 51 for validation, and 82 for testing (the remaining data sets were held out for the clinical feasibility study reported here). After inference, the binary masks were post-processed to produced vectorized contours that were stored as DICOM-RT Structure Set representations. Further details are provided in the section 2 of the **Supplemental material**.

### 2.5 Study design

Under the approval of the institutional review board, we recruited as participants ROs and MDAs who were staff members with HN contouring expertise at our institution for the observational study. Participants were consented by the departmental clinical trials team. Prior to the recruitment period (11/3-11/10/2021), the model was finalized (AI-HN-v3b) and a study protocol pre-specification was publicly released (63). The staff members were eligible to participate if OAR contouring for HN patients was a part of their regular practice and were excluded if they had been involved in the data curation, model training or validation for this project.

The HO datasets, withheld from model training, testing, and validation, were prospectively selected from a pool of retrospective candidate datasets with an emphasis on diversity in terms of anatomic subsites of disease, surgical status (including both definitive and adjuvant RT), and traditional patient demographical information (race, sex, and ethnicity). 20 patient datasets were selected with patient characteristics given in **Table S2** of the **Supplemental materials**. For each patient data set, one set of OAR contours was created by an MDA participant and a second set was generated by the DL model. Using block randomization without replacement (**Figure 1**), a RO participant was allocated to review and revise each set of initial contours (blinded to their origin) to make them acceptable for clinical treatment planning. In this way, the initial MDA contours with RO-revisions is the standard arm of the study, representing the fully manual contouring workflow, and the RO-revisions of the DL contours represents the experimental arm. These arms will be referred to as MDA+RO and DL+RO, respectively. To achieve balance between the arms, each participating RO was assigned an equal number of cases for each arm, and the ROs were not assigned to both arms for the same patient to avoid the effects of recall. Randomization was completed by the statistical team and entered in the REDCap database to ensure blinding for the rest of the study team using a REDCap database (REDCap Cloud, Encintas, CA). A CONSORT-AI reporting checklist is provided in the **Supplemental materials** (section 7).

### 2.6 Study endpoints

#### 2.6.1 Time savings

The primary endpoint was reduction in the total time to complete OAR contouring for participants. Timing was calculated from manual review of the contouring session recorded using screen-capture software (Capture, Kaltura, New York, NY), excluding any significant period of time without activity (greater than 5 minutes). For the MDA+RO arm, the time required for the MDA to perform initial contours and the RO to revise them was collected, and the total time for both participants was computed.

#### 2.6.2 Evaluation of missing structures

The model’s ability to identify and omit missing structures was assessed by computing a contingency table for the presence of the contour compared to the presence in the GS structure set.

#### 2.6.3 Comparison of geometric agreement with gold standard

For each patient, the geometric agreement with the GS contours was assessed with multiple measures for the MDA+RO, and DL+RO contours, as well as the unrevised contours from the autosegmentation model (which will be referred to as the DL arm) with multiple measures: volumetric Dice similarity coefficient (64) (VDSC), surface Dice similarity coefficient (SDCS, with τ=1, 1.5, 2, and 3mm) (19), 95-percentile Hausdorff distance (64) (HD95%), added path length (APL, computed with tolerances of 1, 2, 3, and 5 mm) (65), precision (64), sensitivity (64), contour Dice coefficient (CDC) (66), and the change in volume and centroid of structure.

#### 2.6.4 Comparison of impact on treatment plan dosimetry

To facilitate a comparison of the dosimetric impact of the autosegmentation, a new reference treatment plan was generated for each patient using the OAR structures generated by the DL model before RO revisions. The choice to use the DL contours (without RO revision) for the OARs for the reference plan was made to allow a comparison of the quality of the plan designed using the DL model being evaluated against the GS contours, and to assess the feasibility of running a prospective trial using the unrevised DL contours for treatment planning. The clinical target volumes (CTVs) were taken from the patient’s previously delivered treatment plan, and planning target volumes were generated from them by performing a uniform 3mm expansion (cropped to the patient’s body surface). Each patient had between one and three prescription dose levels. These OARs were briefly reviewed to detect major defects by MDAs (who were not study participants), and minor post-processing was performed consistent with routine clinical practice. The review and post-processing process was not allowed to take more than 15 minutes, no major defects were noted, and the VDSC was compared before and after the post-processing to ensure that no significant changes were made to the contours. The prescription dose levels for each plan were determined by the RO-As based on the department’s guidelines for conventionally fractionated x-ray treatments based on the patient’s disease site and treatment intent, ranging from 54 Gy to 72 Gy total dose in fractions of 1.8 to 2.12 Gy. CMDs with significant HN planning experience worked with the RO-As to create a 6 MV volumetric modulated arc therapy treatment plan, with treatment objectives adapted by the RO-As to the specific patient based on institutional guidelines.

Ultimately, HN RT treatment planning is challenging because it involves balancing a complex set of trade-offs to achieve the optimal plan for a given patient. That means assessing the clinical impact of contour accuracy on treatment planning requires looking not just at the impact on one DVH statistic, but also on how it impacts the overall trade-offs that inform plan quality. To quantify this impact, we adapted the concept of the plan quality metric from Nelms, et al. (67). A plan quality metric scoring template was built from DVH statistics for both target volumes and OARs derived from the institutional planning guidelines for HN cancer (see **Table S2** in the **Supplemental material**). To account for the variation in the number of target levels and OARs between patients within the HO cohort, the plan quality metric score for a given set of contours was reported as the percentage of the maximum possible value of the plan quality metric given the structures present, which we refer to as the normalized plan quality metric (NPQM).

The focus of this study was the agreement of the DVH statistics and NPQM with the GS for the contours on each arm (rather than a plan quality study), so all dosimetric statistics were reported as the absolute value of the difference between the experimental arm and the GS. Using the reference dose distribution for each patient, the mean dose (Dmean) and D0.03cc were computed for the region of the OAR contours that was not overlapping with the PTVs. If the volume of the contour that was non-overlapping with the targets was less than 0.1cc, that contour was excluded from dosimetric analysis. All dose-volume statistics were extracted using a commercial treatment plan quality software (ProKnow Systems, Sanford, FL, USA). The mean value of the absolute difference in the OAR’s mean dose (|ΔDmean|) and D0.03cc (|ΔD0.03cc|) between the arms were computed for all structures, and for each individual OAR. In addition, the percent difference in NPQM relative to the GS (|ΔNPQM|) is reported.

#### 2.6.5 Participant survey

Surveys were administered to understand the RO’s experience reviewing and revising the contours and their perceived quality. After each case was completed, a survey was administered to the ROs who were still blinded to the origin of the contours. The survey included questions on subjective quality, clinical impact, and task load (68, 69) for that case. After all allocated cases were completed, an exit survey was administered to the RO in which cases were unblinded, allowing ROs to comment on the use of the DL model for autosegmentation.

### 2.7 Statistical methods

Data analysis was performed using SAS v9.04 and R v3.6.2. Categorical values are reported in terms of absolute and relative frequencies, while continuous variables are described in terms of mean and 95% confidence intervals (95%-CI). Prior to the study, we hypothesized that a 30% time-savings with the deep learning model would be clinically significant. Based on previous internally collected data on timing results showing a reduction in contouring time of approximately 65% (standard deviation 20%), we conservatively estimated that the time-savings in this study would be 50%. From these estimates, we would have 92.4% power to demonstrate that the time-savings was significantly more than 30% (with a one-sided significance level of 0.025) using a sample size of 20 patient datasets. All other group comparisons were performed using independent two-sided paired *t*-tests with significance level of 0.05.

## 3 Results

### 3.1 Recruitment and study completion

The study recruited 8 ROs and 8 MDAs to participate. The study was run from 12/6/2021 through 1/31/2022, and two sets of RO-revised contours were obtained for each of the 20 patient data sets. However, one CT dataset had to be excluded from comparison due to unintentional data cross-over during the blinding process. Analyses were performed only on the remaining 19 datasets. **Figures 1B** shows the contours for a representative patient from both arms, as well as the un-revised contours from the DL and GS contour sets.

### 3.2 Time savings

The mean contouring times are presented in **Table 1**. The total contouring time for the MDA+RO contouring time was 3.4 hours, compared to 0.7 hours for the revisions to the DL contours, a time savings of 76% (95% CI: 65% - 88%). In addition, the RO revisions to the DL contours showed a non-significant reduction compared to the revisions of the MDA contours of 35% (95% CI, -39%-91%, p=0.09). For all cases, the DL revision time was less than the combined MDA+RO time.

**Table 1:** Mean time (95%-CI) for MDA initial contouring, and physician revisions of the MDA and DL contouring for all patients (N=19).

Arm	MDA Contouring (hour)	RO Revision (hour)	Total (hour)
**MDA+RO**	0.7 (0.6 – 1.6)	1.1 (0.4-4.4)	3.4 (2.9-3.9)
**DL+RO**	n/a	0.7 (0.6 – 1.6)	0.7 (0.6 – 1.6)
**Time Savings**	n/a	35% (65% - 88%)	76% (-39% - 91%)*

*Indicates a statistically significant (p<0.05) difference.

### 3.3 Missing structures

The DL model correctly identified the presence of 818 OAR structures and omitted no structures that were present (100% sensitivity). The DL model correctly identified that 15 structures were not present, and incorrectly identified the presence of 7 that were not present (68% specificity).

For clinical cases at our institution, the ROs are responsible for contouring the BPs or CAs. As part of the blinding process, no empty (placeholder) structures for the BP or CA structures in the structure set were added for the MDA-derived contours. The unanticipated result was that for the MDA+RO arm, the ROs did not add the CAs in any cases and only added 13 pairs of BP contours, although these structures were present in all 19 cases (and were correctly identified by the DL model). As such, the CAs and missing BPs were excluded from all aggregate statistics and comparisons. However, while no comparisons are performed, the geometric similarity of the CAs with the GS is shown for the DL and DL+RO arm for reference.

### 3.4 Geometric agreement with gold standard

All geometric and dosimetric comparisons and analysis were performed only for structures that were present in all 4 arms for a given patient. With this criteria, there were 777 structures (3108 total contours) that were eligible for analysis from the 19 patients, representing 40 OARs.

#### 3.4.1 DL+RO arm *vs* MDA+RO arm

Comparing the agreement between the final, RO-revised contours from the DL model (DL+RO) to the final contours from the MDA+RO allows us to assess the impact of the DL model on the current workflow. The mean value of the geometric agreement metrics for all experimental contours compared to the GS are given in **Table 2** (additional metrics are in section 4 of the **Supplemental material**). The agreement with the GS of the DL+RO contours was significantly better than for the MDA+RO contours for all geometric metrics except for sensitivity and specificity (for which there was no significant difference). The mean VDSC for the DL+RO contours was 0.86 ± 0.01 compared to 0.78 ± 0.01 for the MDA+RO.

**Table 2:** Mean value (95%-CI) of select metrics of geometric agreement between each experimental arm and the GS for all contours for comparison (N=777).

Metric	DL (95%-CI)	DL+RO (95%-CI)	MDA+RO (95%-CI)
**VDSC**	0.87 (0.01)*	0.86 (0.01)	0.78 (0.01)*
**HD95% (mm)**	2.2 (0.1)*	2.8 (0.2)	5.3 (1.2)*
**APL-1mm (mm)**	19.1 (1.9)*	21.6 (2.2)	25.5 (2.5)*
**SDCS-1mm**	0.81 (0.01)*	0.78 (0.01)	0.70 (0.01)*
**ΔVolume (cc)**	0.9 (0.6)	1.3 (0.8)	-3.0 (1.2)*
**ΔCentroid (mm)**	0.8 (0.1)	0.9 (0.1)	1.8 (0.3)*
**Precision**	0.87 (0.01)*	0.86 (0.01)	0.77 (0.01)*
**Sensitivity**	0.89 (0.01)*	0.87 (0.01)	0.88 (0.01)
**Specificity**	1.0 (0.0)*	1.0 (0.0)	1.0 (0.0)
**CDC-1mm**	0.77 (0.01)*	0.73 (0.01)	0.64 (0.02)*

*Indicates a statistically significant (p<0.05) difference with the DL+RO arm.

**Figure 3:**
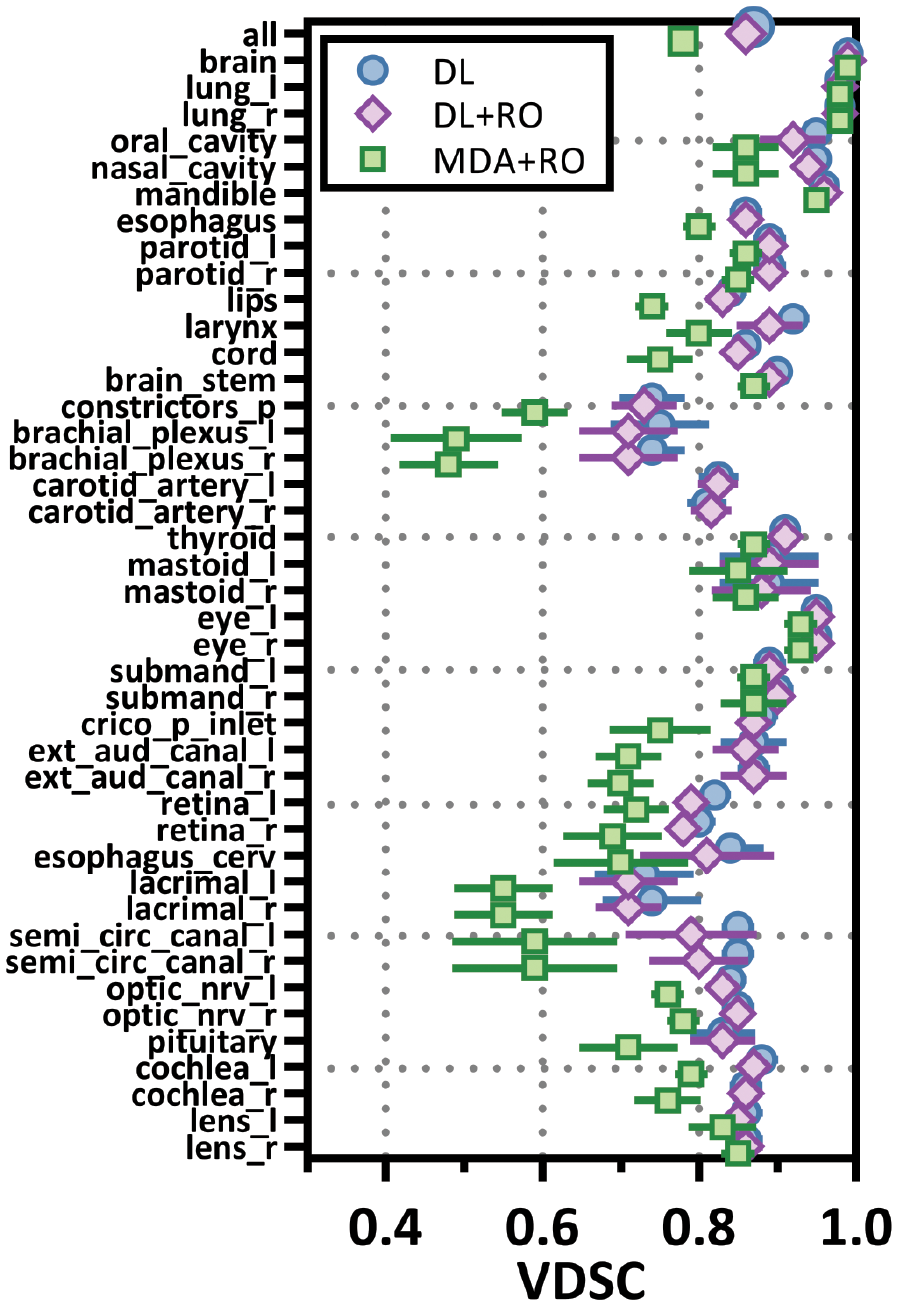
Mean volumetric Dice similarity coefficient (VDSC) and 95% confidence intervals for the unrevised deep learning (DL), radiation oncologist (RO) revised deep learning (DL+RO), and RO-revisions to the initial MDA contours (MDA+RO), categorized by organ-at-risk type and for all OARs. OARs listed in order of decreasing volume.

**Figure 4:**
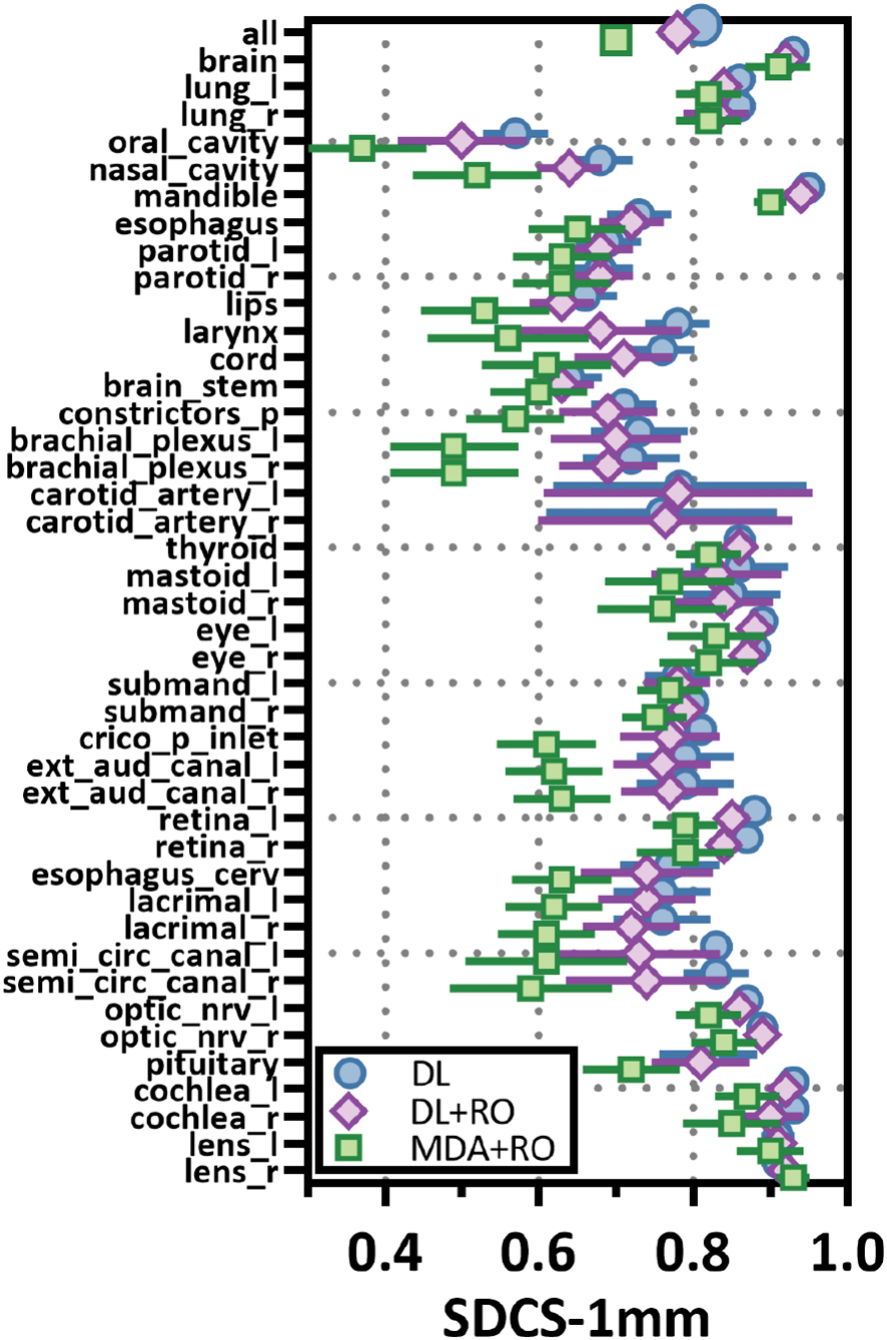
Mean surface Dice similarity coefficient (SDSC) with a 1mm tolerance, and 95% confidence intervals for the unrevised deep learning (DL), radiation oncologist (RO) revised deep learning (DL+RO), and RO-revisions to the initial MDA contours (MDA+RO), categorized by organ-at-risk type and for all OARs. OARs listed in order of decreasing volume.

**Figure 5:**
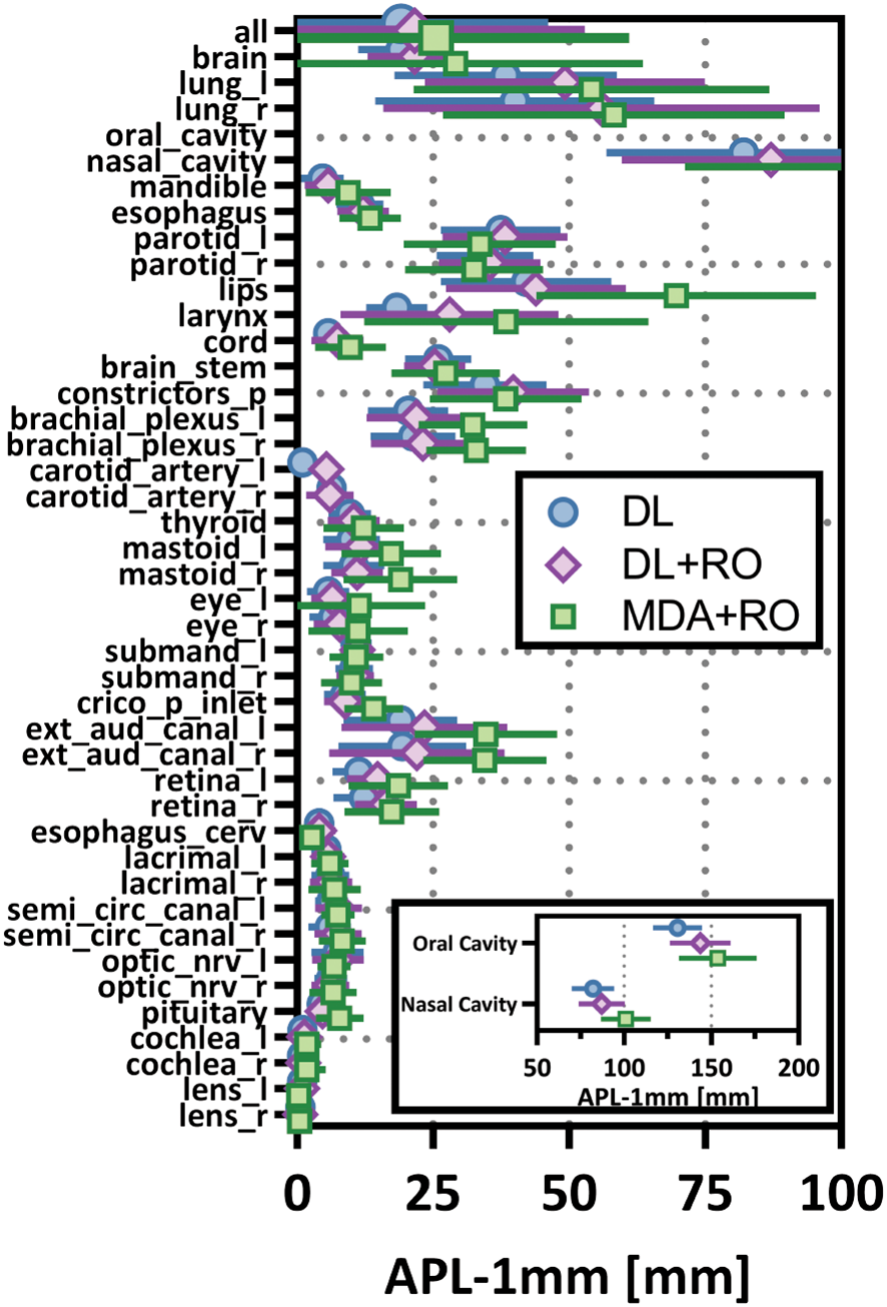
Mean added path length (APL) with a 1mm tolerance, and 95% confidence intervals for the unrevised deep learning (DL), radiation oncologist (RO) revised deep learning (DL+RO), and RO-revisions to the initial MDA contours (MDA+RO), categorized by organ-at-risk type and for all OARs. OARs listed in order of decreasing volume.

**Figure 6:**
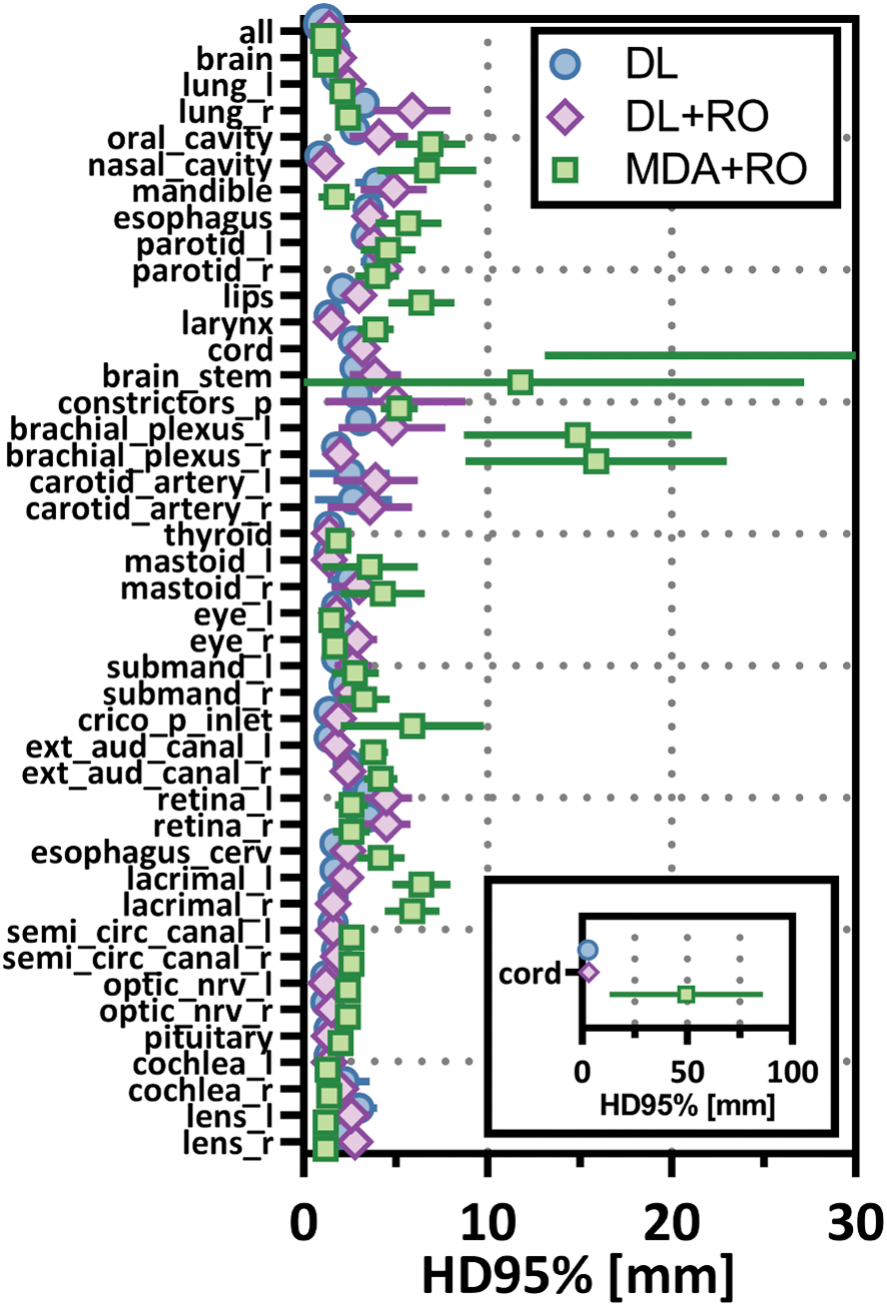
Mean 95%-percentile Hausdorff distance (HD95%) with a 1mm tolerance, and 95% confidence intervals for the unrevised deep learning (DL), radiation oncologist (RO) revised deep learning (DL+RO), and RO-revisions to the initial MDA contours (MDA+RO), categorized by organ-at-risk type and for all OARs. OARs listed in order of decreasing volume.

Categorizing the contours by OAR, the mean VDSC, HD95%, APL-1mm, and SDCS-1mm are shown in **Figures 3**–**6** and **Table S5** of the **Supplemental material** l. 32 of the OARs had a mean VDSC greater than 0.8 for DL+RO arm, compared to 20 for the MDA+RO arm. In addition, all OARs showed either a significantly better agreement for the DL+RO contours or no difference, compared to the MDA+RO by all 4 metrics (summarized in **Table 3** from the full data provided in **Table S5** of the **Supplemental material**), with one exception (while the pharyngeal constrictor muscles showed better agreement with VDSC and SDCS-1mm for the DL+RO arm and no significant difference as measured by HD95%, there was a better agreement for the MDA+RO as measured by APL-1mm). Better agreement was demonstrated for most OARs for the DL+RO arm using VDSC and APL, while most showed no difference according to HD95%.and SDCS-1mm.

**Table 3:** Number of OARs demonstrating statistically significant differences (p<0.05) in agreement with gold standard between arms for select measures of geometric similarity between the DL and DL+RO contours, and MDA+RO and DL+RO contours, and if the difference is significant, which arm showed better agreement with gold standard.

Metric	DL v DL+RO			MDA+RO v DL+RO		
	No Difference	Favor DL	Favor DL+RO	No Difference	Favor DL+RO	Favor MDA+ RO
**VDSC**	38	2	0	8	32	0
**HD95% (mm)**	35	5	0	32	8	0
**APL-1mm (mm)**	29	11	0	32	7	1
**SDCS-1mm**	30	10	0	27	13	0

#### 3.4.2 DL+RO arm *vs* DL arm

The comparison of the unrevised (DL) and revised (DL+RO) contours enables an assessment of the quality and consistency of the DL model. The mean agreement with the GS for all contours was significantly better for the unrevised contours compared to the revised ones (**Table 2**) except for volume and centroid (no statistical differences). Overall, there was very strong agreement with the GS both before and after the revisions: mean VDSC was 0.87 ± 0.01 and 0.86 ± 0.01 for the DL and DL+RO contours, respectively. In addition, before Dean RO-revisions, only 4 of the 777 (<1%) individual contours from the DL model had a VDSC < 0.5 compared to GS, and none of the RO-revisions improved agreement to be greater than 0.5. In addition, there were 3 contours that showed VDSC > 0.5 before RO revisions to the DL model contours, but the revisions reduced agreement below 0.5.

**Figure 7:**
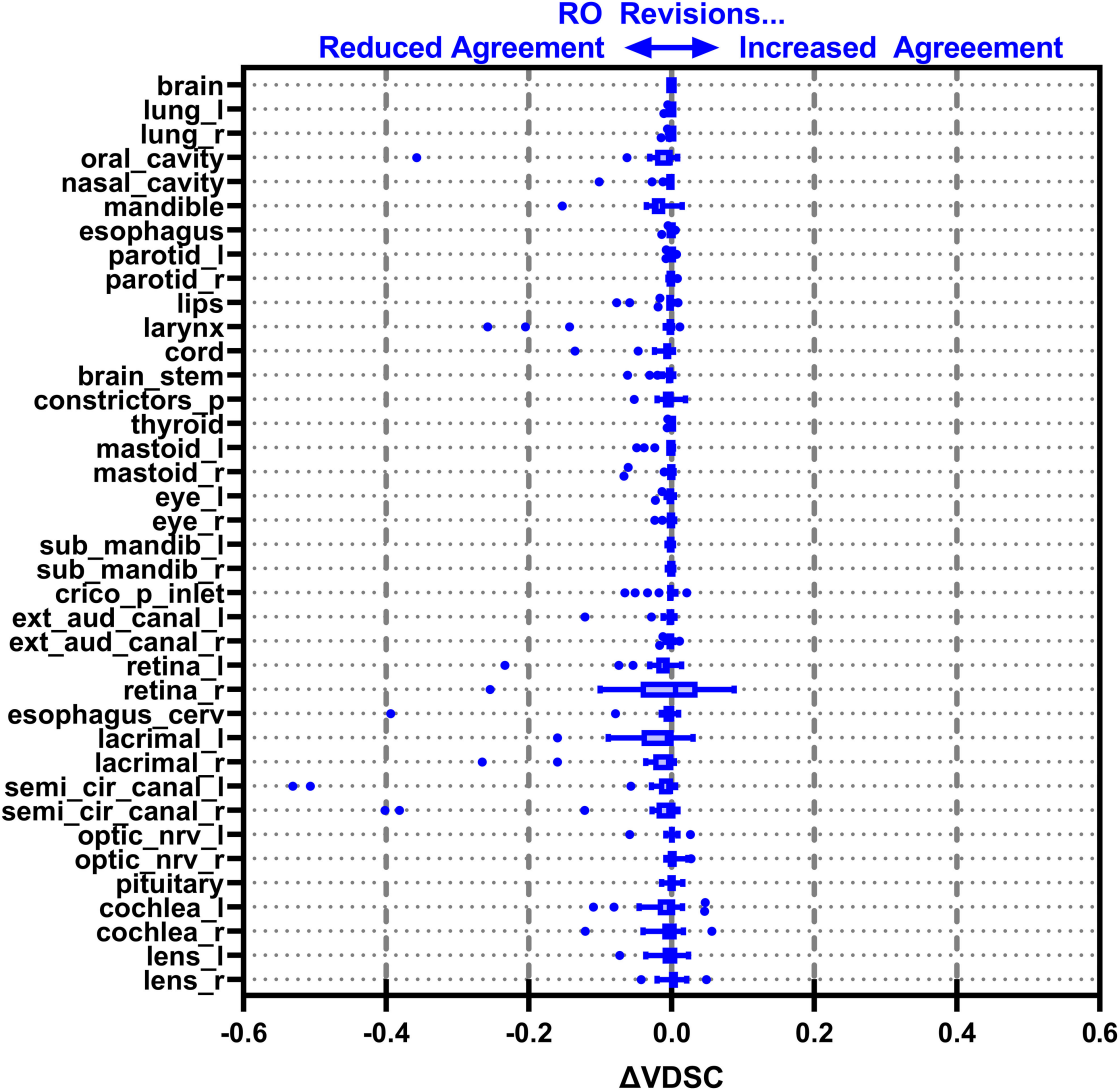
Box-and-whisker plot showing the change in agreement with the gold standard (GS) before (DL) and after (DL+RO) the radiation oncologist’s (RO’s) revisions to the deep learning (DL) contours measured by volumetric Dice similarity coefficient (VDSC). A positive value indicates that the revisions improved agreement with the GS, and a negative value indicates reduced agreement. OARs listed in order of decreasing volume.

**Figures 3**–**6** also show the agreement with the GS broken down by OAR for both the DL and DL+RO contours (as well as in **Table S5** of the **Supplemental material**). For each of the metrics, none of the OARs showed significantly better agreement with GS after RO revisions compared to before (**Table 3**): in fact, most showed no differences. However, 2 more OARs had a mean VDSC greater than 0.8 for the DL arm, bringing the total to 34 compared to 32 for the DL+RO arm. The explicit change in geometric agreement with the GS of the RO’s revisions is summarized in **Figure 7**. The mean change in the VDSC compared to GS was not significantly different from zero (p=0.8). There were no individual cases in which the RO-revisions resulted in an improvement of larger than 0.05, and 22 (3%) revisions decreased the agreement by more than 0.1.

### 3.5 Dosimetric impact

The average magnitude of the difference of the D0.03cc, mean dose, and NPQM relative to the GS for the DL, DL+RO, MDA+RO contours for all OARs are shown in **Table 4**. There is no significant difference in the agreement of the NPQM with the GS between the DL+RO contours and either the DL or the MDA+RO contours. In terms of D0.03cc and Dmean, the agreement with the GS was significantly better for the DL+RO contours compared to the MDA+RO contours, (DL contours showed significantly better agreement than DL+RO). The dosimetric data for each OAR are shown in **Table S11** of the **Supplemental material**. The DL+RO contours had significantly better agreement than the MDA+RO contours with the GS in terms of D0.03cc and the mean dose for 5 and 9 of the OARs, respectively, while there was no significant difference in agreement between the DL and DL+RO contours for any OAR.

**Table 4:** Mean agreement with gold standard using dosimetric comparisons.

Metric	DL (95%-CI)	DL+RO (95%-CI)	MDA+RO (95%-CI)
**|ΔD0.03cc| (Gy)**	0.6 (0.1)*	0.8 (0.2)	1.2 (0.2)*
**|ΔDmean| (Gy)**	0.4 (0.1)	0.4 (0.1)	0.8 (0.1)*
**|ΔNPQM| (%)**	1.3 (0.8)	1.7 (1.2)	3.0 (1.4)

*Indicates a statistically significant (p<0.05) difference compared to the DL+RO arm.

### 3.6 Participant survey

**Figure 8:**
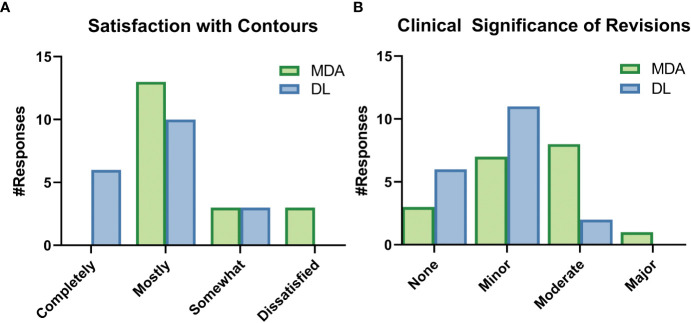
Selected results from the survey given to radiation oncologists (ROs) after completion of revision of a particular dataset. These survey questions were asked while the ROs were still blinded to origin of the initial contours, either by a medical dosimetry assistant (MDA) or the deep learning (DL) model. **(A)** The ROs were asked to rate their satisfaction with the initial contours, as well as **(B)** rate the clinical significance of the revisions to the contours.

While still blinded to the origin of the contours, ROs reported they did not need to make any edits of major clinical significance to the DL contours and reported fewer edits of any significance compared to the MDA contours (**Figure 8**). ROs rated their subjective impression of the quality of all DL contours from “somewhat satisfied” to “completely satisfied” and all indicated that they were “very interested” in using the DL contours for clinical cases. Complete survey results are available in section 6 of the **Supplemental materials**.

## 4 Discussion

The potential of this DL autosegmentation model to be integrated in the clinic and undergo external validation was investigated using a multi-observer randomized trial. Eight ROs with significant experience in HN cancer participated in the study, reviewing and revising two sets of contours (one manually delineated by MDAs, the other from the DL model) for 19 patient datasets. The scale of this study, in terms of both number of participants and patient data sets, is meant to represent the clinical practice at our large institution (and applicability at other institutions). The use of comparisons to an independent GS dataset is a key novelty of this study: created without the demands on the time that routine clinical practice imposes, the GS in this study represents the realization of the institutional contouring standards derived from international consensus guidelines. The independence of the GS enables us to assess the effect of the DL model in terms of agreement with the institutional standards, in addition to finding differences between the standard and DL-assisted workflows.

The DL-assisted workflow demonstrated significant time savings compared to the standard workflow of 76% reduction (2.7 hours). Furthermore, this reduction in time may underestimate the true time savings, as the CAs and many of the BPs were not contoured in the MDA+RO arm but were reviewed and revised by the ROs on the DL+RO arm. These are complex and often time-consuming OARs to contour: during the GS curation process, it took an average of 33 minutes to manually contour the CAs and 56 minutes for the BPs (21). While all of the OARs may not be required for treatment planning for an individual patient, in our existing workflow the MDAs are expected to contour all of them (except the BP and CA), so this increased efficiency translates to the clinical practice. Crucially, these efficiency gains did not result in lower quality contours. On the contrary, whether for the aggregate statistics for all 777 analyzed structures or categorized by OAR, the geometric and dosimetric agreement with the GS either showed no statistical difference or favored the DL+RO arm relative to the MDA+RO (except for the pharyngeal constrictors, which showed improved agreement for the MDA+RO using APL-1mm but not any other metrics). Ultimately, the DL model has potential to reduce contouring time while improving standardization across the clinic.

The RO’s revisions to the DL contours tended to be very minimal, indicating excellent performance by the model. The mean change in VDSC before and after the revisions was not significantly different from zero and there were no revisions resulting in an increase of VDSC greater than 0.06. This finding holds with SDCS-1mm and APL-1mm, which have been shown to have a strong correlation with time-savings (19, 32, 65), and HD95% which is often used to assess treatment planning impact. Importantly, the quality of the DL contours was evident by the fact that the ROs required less time to revise them compared to the MDA contours (an average reduction of 0.4 hours per patient, or 35%). This was also evident in the RO’s subjective assessment of contour quality. While still blinded to the origin of the contours, the physicians indicated higher satisfaction with the DL contours and reported that none of the revisions were of major clinical significance. After unblinding, the ROs all expressed interest in using this tool clinically. The contours produced by the DL model have potential to be used for clinical treatment planning with at most minor revisions.

**Figure 9:**
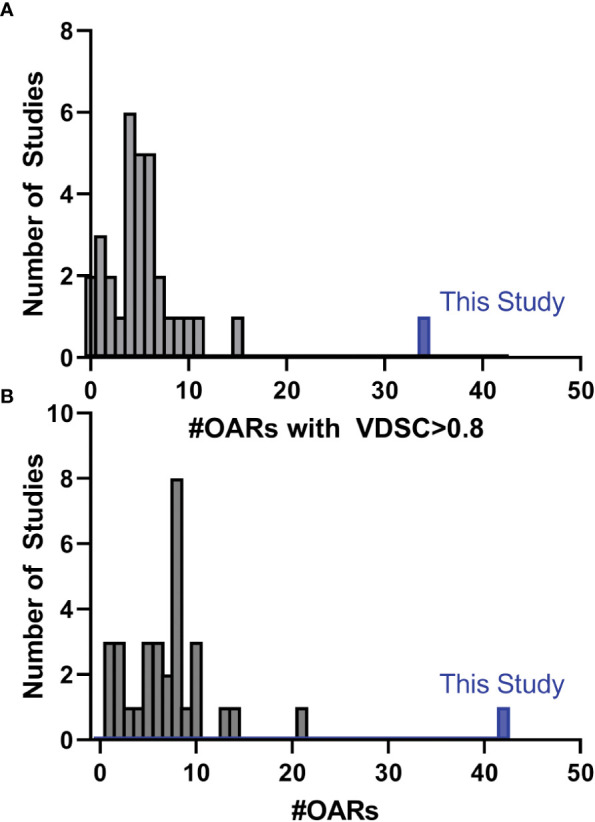
Histogram showing the number of studies found during the literature that reported the number of distinct OARs (of those used in this study) that had **(A)** a mean VDSC greater than 0.8 or **(B)** the total number of OARs included in the study that corresponded with those in this study. Detailed discussion is given in section 5 of the **Supplemental material**.

While there have been many published reports of DL-based autosegmentation using 3D U-net architectures, as well as other approaches, the performance of this model is noteworthy (19, 25–62). Model performance was most frequently reported in terms of VDSC, and often a threshold of 0.8 was used for clinical acceptability. In this study, for the 777 analyzed structures, the mean VDSC for the unrevised DL contours was 0.87 ± 0.01, and that 34 of the included OARs showed a mean VDSC of greater than 0.8 (with none less than 0.73). In comparison, none of the other studies report more than 16 OARs which showed a VDSC greater than 0.8 with their reference structures (**Figure 9**, please see section 5 of the **Supplemental materials** for further discussion). In addition, this model contains 10 OARs that are not reported elsewhere in the literature. This is the first demonstration of a DL-model that produces the comprehensive set of HN OAR contours needed for treatment planning at our institution: the model demonstrated excellent performance for 42 OARs.

The accuracy, reliability, consistency of this of model for all 42 OARs against the institutional GS reflects well on both the quality and quantity of the curated data as well as the appropriateness of the model architecture and training process. Providing adequate resources to produce a large, standardized, and high-quality dataset provided a strong foundation for both model training and validation. In addition, deep learning in general (and 3D U-Nets specifically) have been shown to have advantages over other reported approaches to HN autosegmentation (16, 19, 23, 24, 32, 34). The architecture of this model is unique because of both the size of the 3D sub-volumes it uses and the depth of the network (six layers), which enabled the creation of a model of sufficient complexity to tackle this challenging problem. Ultimately, this process required a very significant investment in terms of curation efforts and model training. In the end, differences in the model architecture, training method, and training data contribute to the differences between this model and previously reported models.

The purpose of this study was to assess whether the current DL model was ready for clinical integration and external validation. As such, although the hold out cohort of 19 patients was selected to be as representative of the US population as possible (and is large compared to other studies), it is still a limited sample size and number of participants (8 ROs). Naturally, this single-institution study on retrospective data is limited in terms of more general applicability: prospective study of the impact on clinical integration in our clinic and an external validation study are in development. Since the institutional standards are based on international consensus guidelines, this model could have applicability for many institutions. The benefits of an accurate and widely available autosegmentation tool would include providing access to comprehensive organ-sparing RT to patients throughout the world, particularly in low-resource environments.

## 5 Conclusion

A DL model capable of highly accurate autosegmentation of a comprehensive set of 42 HN OARs was demonstrated to provide significant time-savings in a blinded randomized controlled trial involving 19 patient datasets and 8 ROs. The DL contours have the potential to be used for clinical treatment planning with, at most, minor revisions. An interventional clinical trial is being developed to prospectively assess the capability of the model in patient care as well as external validation.

## Data availability statement

The raw data supporting the conclusions of this article will be made available by the authors, without undue reservation.

## Ethics statement

The studies involving human participants were reviewed and approved by Mayo Clinic Institutional Review Board. The patients/participants provided their written informed consent to participate in this study. Written informed consent was obtained from the individual(s) for the publication of any potentially identifiable images or data included in this article.

## Author contributions

All authors were involved in study design, execution of the study, data analysis, writing of the manuscript and final manuscript approval. TD, TL and JL were responsible for statistical analysis. All authors contributed to the article and approved the submitted version.

## References

[1] Sung H Ferlay J Siegel RL Laversanne M Soerjomataram I Jemal A. Global cancer statistics 2020: GLOBOCAN estimates of incidence and mortality worldwide for 36 cancers in 185 countries. CA Cancer J Clin (2021) 71(3):209–49. doi: 10.3322/caac.21660 33538338

[2] Patterson RH Fischman VG Wasserman I Siu J Shrime MG Fagan JJ. Global burden of head and neck cancer: economic consequences, health, and the role of surgery. Otolaryngol Head Neck Surg (2020) 162(3):296–303. doi: 10.1177/0194599819897265 31906785

[3] Atun R Jaffray DA Barton MB Bray F Baumann M Vikram B. Expanding global access to radiotherapy. Lancet Oncol (2015) 16(10):1153–86. doi: 10.1016/S1470-2045(15)00222-3 26419354

[4] Peters LJ O’Sullivan B Giralt J Fitzgerald TJ Trotti A Bernier J. Critical impact of radiotherapy protocol compliance and quality in the treatment of advanced head and neck cancer: results from TROG 02.02. J Clin Oncol (2010) 28(18):2996–3001. doi: 10.1200/JCO.2009.27.4498 20479390

[5] Boero IJ Paravati AJ Xu B Cohen EE Mell LK Le Q-T. Importance of radiation oncologist experience among patients with head-and-neck cancer treated with intensity-modulated radiation therapy. J Clin Oncol (2016) 34(7):684. doi: 10.1200/JCO.2015.63.9898 26729432 PMC4872027

[6] Hawkins PG Kadam AS Jackson WC Eisbruch A. Organ-sparing in radiotherapy for head-and-Neck cancer: Improving quality of life. Semin Radiat Oncol (2018) 28(1):46–52. doi: 10.1016/j.semradonc.2017.08.002 29173755

[7] Kam D Salib A Gorgy G Patel TD Carniol ET Eloy JA. Incidence of suicide in patients with head and neck cancer. JAMA Otolaryngol Head Neck Surg (2015) 141(12):1075–81. doi: 10.1001/jamaoto.2015.2480 26562764

[8] Osazuwa-Peters N Simpson MC Zhao L Boakye EA Olomukoro SI Deshields T. Suicide risk among cancer survivors: Head and neck versus other cancers. Cancer (2018) 124(20):4072–9. doi: 10.1002/cncr.31675 30335190

[9] Arthurs E Hanna TP Zaza K Peng Y Hall SF. Stroke after radiation therapy for head and neck cancer: What is the risk? Int J Radiat Oncol Biol Phys (2016) 96(3):589–96. doi: 10.1016/j.ijrobp.2016.07.007 27681754

[10] Smith GL Smith BD Buchholz TA Giordano SH Garden AS Woodward WA. Cerebrovascular disease risk in older head and neck cancer patients after radiotherapy. J Clin Oncol (2008) 26(31):5119–25. doi: 10.1200/JCO.2008.16.6546 PMC4879715 18725647

[11] Swisher–McClure S Mitra N Lin A Ahn P Wan F O’Malley B. Risk of fatal cerebrovascular accidents after external beam radiation therapy for early-stage glottic laryngeal cancer. Head Neck. (2014) 36(5):611–6. doi: 10.1002/hed.23342 PMC3795979 23595858

[12] Vorwerk H Zink K Schiller R Budach V Böhmer D Kampfer S. Protection of quality and innovation in radiation oncology: the prospective multicenter trial the German society of radiation oncology (DEGRO-QUIRO study). Strahlenther Onkol. (2014) 190(5):433–43. doi: 10.1007/s00066-014-0634-0 24595416

[13] van der Veen J Gulyban A Willems S Maes F Nuyts S. Interobserver variability in organ at risk delineation in head and neck cancer. Radiot Oncol (2021) 16(1):1–11. doi: 10.1186/s13014-020-01677-2 PMC8240214 34183040

[14] Geets X Daisne J-F Arcangeli S Coche E De Poel M Duprez T. Inter-observer variability in the delineation of pharyngo-laryngeal tumor, parotid glands and cervical spinal cord: comparison between CT-scan and MRI. Radiat Oncol (2005) 77(1):25–31. doi: 10.1016/j.radonc.2005.04.010 15919126

[15] Peng Y-l Chen L Shen G-z Li Y-n Yao J-j Xiao W-w. Interobserver variations in the delineation of target volumes and organs at risk and their impact on dose distribution in intensity-modulated radiation therapy for nasopharyngeal carcinoma. Oral Oncol (2018) 82:1–7. doi: 10.1016/j.oraloncology.2018.04.025 29909882

[16] Huynh E Hosny A Guthier C Bitterman DS Petit SF Haas-Kogan DA. Artificial intelligence in radiation oncology. Nat Rev Clin Oncol (2020) 17(12):771–81. doi: 10.1038/s41571-020-0417-8 32843739

[17] Vrtovec T Močnik D Strojan P Pernuš F Ibragimov B. Auto-segmentation of organs at risk for head and neck radiotherapy planning: From atlas-based to deep learning methods. Med Phys (2020) 47(9):e929–e50. doi: 10.1002/mp.14320 32510603

[18] Cardenas CE Yang J Anderson BM Court LE Brock KB. Advances in auto-segmentation. Semin Radiat Oncol (2019) 29(3):185–97. doi: 10.1016/j.semradonc.2019.02.001 31027636

[19] Nikolov S Blackwell S Zverovitch A Mendes R Livne M De Fauw J. Clinically applicable segmentation of head and neck anatomy for radiotherapy: deep learning algorithm development and validation study. J Med Internet Res (2021) 23(7):e26151. doi: 10.2196/26151 34255661 PMC8314151

[20] Brouwer CL Steenbakkers RJ Bourhis J Budach W Grau C Grégoire V. CT-based delineation of organs at risk in the head and neck region: DAHANCA, EORTC, GORTEC, HKNPCSG, NCIC CTG, NCRI, NRG oncology and TROG consensus guidelines. Radiother Oncol (2015) 117(1):83–90. doi: 10.1016/j.radonc.2015.07.041 26277855

[21] Tryggestad E Anand A Beltran C Brooks J Cimmiyotti J Grimaldi N. Scalable radiotherapy data curation infrastructure for deep-learning based autosegmentation of organs-at-risk: A case study in head and neck cancer. Front Oncol (2022) 12. doi: 10.3389/fonc.2022.936134 PMC9464982 36106100

[22] Mayo CS Moran JM Bosch W Xiao Y McNutt T Popple R. American Association of physicists in medicine task group 263: Standardizing nomenclatures in radiation oncology. Int J Radiat Oncol Biol Phys (2018) 100(4):1057–66. doi: 10.1016/j.ijrobp.2017.12.013 PMC7437157 29485047

[23] Antonelli M Reinke A Bakas S Farahani K Kopp-Schneider A Landman BA. The medical segmentation decathlon. Nat Commun (2022) 13(1):4128. doi: 10.1038/s41467-022-30695-9 35840566 PMC9287542

[24] Siddique N Paheding S Elkin CP Devabhaktuni V. U-Net and its variants for medical image segmentation: A review of theory and applications. IEEE Access. (2021) 9:82031–57. doi: 10.1109/ACCESS.2021.3086020

[25] Zhong Y Yang Y Fang Y Wang J Hu W. A preliminary experience of implementing deep-learning based auto-segmentation in head and neck cancer: a study on real-world clinical cases. Front Oncol (2021) 1572. doi: 10.3389/fonc.2021.638197 PMC8132944 34026615

[26] Van der Veen J Willems S Deschuymer S Robben D Crijns W Maes F. Benefits of deep learning for delineation of organs at risk in head and neck cancer. Radiother Oncol (2019) 138:68–74. doi: 10.1016/j.radonc.2019.05.010 31146073

[27] Fang Y Wang J Ou X Ying H Hu C Zhang Z. The impact of training sample size on deep learning-based organ auto-segmentation for head-and-neck patients. Phys Med Biol (2021) 66(18):185012. doi: 10.1088/1361-6560/ac2206 34450599

[28] Amjad A Xu J Thill D Lawton C Hall W Awan MJ. General and custom deep learning autosegmentation models for organs in head and neck, abdomen, and male pelvis. Med Phys (2022) 49(3):1686–700. doi: 10.1002/mp.15507 PMC8917093 35094390

[29] Thor M Iyer A Jiang J Apte A Veeraraghavan H Allgood NB. Deep learning auto-segmentation and automated treatment planning for trismus risk reduction in head and neck cancer radiotherapy. Phys Imaging Radiat Oncol (2021) 19:96–101. doi: 10.1016/j.phro.2021.07.009 34746452 PMC8552336

[30] Wang W Wang Q Jia M Wang Z Yang C Zhang D. Deep learning-augmented head and neck organs at risk segmentation from CT volumes. Front Phys (2021) 612. doi: 10.3389/fphy.2021.743190

[31] Brunenberg EJ Steinseifer IK van den Bosch S Kaanders JH Brouwer CL Gooding MJ. External validation of deep learning-based contouring of head and neck organs at risk. Phys Imaging Radiat Oncol (2020) 15:8–15. doi: 10.1016/j.phro.2020.06.006 33458320 PMC7807543

[32] Costea M Zlate A Durand M Baudier T Grégoire V Sarrut D. Comparison of atlas-based and deep learning methods for organs at risk delineation on head-and-neck CT images using an automated treatment planning system. Radiothe Oncol (2022) 177:61–70. doi: 10.1016/j.radonc.2022.10.029 36328093

[33] Fritscher K Raudaschl P Zaffino P Spadea MF Sharp GC Schubert R. Deep neural networks for fast segmentation of 3D medical images, in: International Conference on Medical Image Computing and Computer-Assisted Intervention, (New York, NY, US: Springer Cham) (2016).

[34] Ibragimov B Xing L. Segmentation of organs-at-risks in head and neck CT images using convolutional neural networks. Med Phys (2017) 44(2):547–57. doi: 10.1002/mp.12045 PMC5383420 28205307

[35] Močnik D Ibragimov B Xing L Strojan P Likar B Pernuš F. Segmentation of parotid glands from registered CT and MR images. Phys Med (2018) 52:33–41. doi: 10.1016/j.ejmp.2018.06.012 30139607 PMC6110103

[36] Ren X Xiang L Nie D Shao Y Zhang H Shen D. Interleaved 3D-CNNs for joint segmentation of small-volume structures in head and neck CT images. Med Phys (2018) 45(5):2063–75. doi: 10.1002/mp.12837 PMC5948179 29480928

[37] Zhong T Huang X Tang F Liang S Deng X Zhang Y. Boosting-based cascaded convolutional neural networks for the segmentation of CT organs-at-risk in nasopharyngeal carcinoma. Med Phys (2019) 46(12):5602–11. doi: 10.1002/mp.13825 31529501

[38] Hänsch A Schwier M Gass T Morgas T Haas B Klein J. Comparison of different deep learning approaches for parotid gland segmentation from CT images. Proceeding of SPIE 10575. Houston, TX, US: SPIE (2018).

[39] Zhu W Huang Y Tang H Qian Z Du N Fan W. AnatomyNet: Deep 3D squeeze-and-excitation U-nets for fast and fully automated whole-volume anatomical segmentation. bioRxiv (2018) 392969:1–14. doi: 10.1101/392969

[40] Tong N Gou S Yang S Ruan D Sheng K. Fully automatic multi-organ segmentation for head and neck cancer radiotherapy using shape representation model constrained fully convolutional neural networks. Med Phys (2018) 45(10):4558–67. doi: 10.1002/mp.13147 PMC6181786 30136285

[41] Liang S Tang F Huang X Yang K Zhong T Hu R. Deep-learning-based detection and segmentation of organs at risk in nasopharyngeal carcinoma computed tomographic images for radiotherapy planning. Eur Radiol (2019) 29(4):1961–7. doi: 10.1007/s00330-018-5748-9 30302589

[42] Willems S Crijns W Greca Saint-Esteven AL Veen JVD Robben D Depuydt T. Implementation of DeepVoxNet for Auto-Delineation of Organs at Risk in Head and Neck Cancer Patients in Radiotherapy. In: OR 2.0 Context-aware operating theaters, computer assisted robotic endoscopy, clinical image-based procedures, and skin image analysis. New York, NY, USA: Springer, Cham (2018) 1104:223–32. doi: 10.1007/978-3-030-01201-4_24

[43] Kodym O Španěl M Herout A. Segmentation of head and neck organs at risk using cnn with batch dice loss. In: Brox T. Bruhn A. Fritz M. (Eds.) Pattern Recognition. GCPR 2018 - Lecture Notes in Computer Science. Cham: Springer. (2018) 105–114. doi: 10.1007/978-3-030-12939-2_8

[44] Wang Y Zhao L Wang M Song Z. Organ at risk segmentation in head and neck CT images using a two-stage segmentation framework based on 3D U-net. IEEE Access. (2019) 7:144591–602. doi: 10.1109/ACCESS.2019.2944958

[45] Men K Geng H Cheng C Zhong H Huang M Fan Y. Technical note: More accurate and efficient segmentation of organs-at-risk in radiotherapy with convolutional neural networks cascades. Med Phys (2019) 46(1):286–92. doi: 10.1002/mp.13296 PMC6322972 30450825

[46] Tappeiner E Pröll S Hönig M Raudaschl PF Zaffino P Spadea MF. Multi-organ segmentation of the head and neck area: an efficient hierarchical neural networks approach. Int J Comput Assist Radiol Surg (2019) 14(5):745–54. doi: 10.1007/s11548-019-01922-4 30847761

[47] Rhee DJ Cardenas CE Elhalawani H McCarroll R Zhang L Yang J. Automatic detection of contouring errors using convolutional neural networks. Med Phys (2019) 46(11):5086–97. doi: 10.1002/mp.13814 PMC6842055 31505046

[48] Tang H Chen X Liu Y Lu Z You J Yang M. Clinically applicable deep learning framework for organs at risk delineation in CT images. Nat Mach Intell (2019) 1(10):480–91. doi: 10.1038/s42256-019-0099-z

[49] van Rooij W Dahele M Ribeiro Brandao H Delaney AR Slotman BJ Verbakel WF. Deep learning-based delineation of head and neck organs at risk: Geometric and dosimetric evaluation. Int J Radiat Oncol Biol Phys (2019) 104(3):677–84. doi: 10.1016/j.ijrobp.2019.02.040 30836167

[50] Gou S Tong N Qi S Yang S Chin R Sheng K. Self-channel-and-spatial-attention neural network for automated multi-organ segmentation on head and neck CT images. Phys Med Biol (2020) 65(24):245034. doi: 10.1088/1361-6560/ab79c3 32097892

[51] Liang S Thung KH Nie D Zhang Y Shen D. Multi-view spatial aggregation framework for joint localization and segmentation of organs at risk in head and neck CT images. IEEE Trans Med Imag (2020) 39(9):2794–805. doi: 10.1109/TMI.2020.2975853 32091997

[52] Qiu B Guo J Kraeima J Glas HH Zhang W Borra RJ. Recurrent convolutional neural networks for 3D mandible segmentation in computed tomography. J Pers Med (2021) 11(6):492. doi: 10.3390/jpm11060492 34072714 PMC8229770

[53] Sun S Liu Y Bai N Tang H Chen X Huang Q. Attentionanatomy: A unified framework for whole-body organs at risk segmentation using multiple partially annotated datasets, in: 2020 IEEE 17th International Symposium on Biomedical Imaging (ISBI), Iowa City, IA, USA: IEEE. (2020) 1–5pp. doi: 10.1109/ISBI45749.2020.9098588.

[54] van Dijk LV Van den Bosch L Aljabar P Peressutti D Both S J.H.M. Steenbakkers R. Improving automatic delineation for head and neck organs at risk by deep learning contouring. Radiot Oncol (2020) 142:115–23. doi: 10.1016/j.radonc.2019.09.022 31653573

[55] Wong J Fong A McVicar N Smith S Giambattista J Wells D. Comparing deep learning-based auto-segmentation of organs at risk and clinical target volumes to expert inter-observer variability in radiotherapy planning. Radiot Oncol (2020) 144:152–8. doi: 10.1016/j.radonc.2019.10.019 31812930

[56] Chan JW Kearney V Haaf S Wu S Bogdanov M Reddick M. A convolutional neural network algorithm for automatic segmentation of head and neck organs at risk using deep lifelong learning. Med Phys (2019) 46(5):2204–13. doi: 10.1002/mp.13495 30887523

[57] Gao Y Huang R Chen M Wang Z Deng J Chen Y. FocusNet: Imbalanced large and small organ segmentation with an end-to-end deep neural network for head and neck CT images, in: International Conference on Medical Image Computing and Computer-Assisted Intervention. New York, NY, USA: Springer. (2019) 829–38pp. doi: 10.1007/978-3-030-32248-9_92

[58] Lei W Wang H Gu R Zhang S Zhang S Wang G. DeepIGeoS-V2: deep interactive segmentation of multiple organs from head and neck images with lightweight CNNs. In: Large-Scale annotation of biomedical data and expert label synthesis and hardware aware learning for medical imaging and computer assisted intervention. New York, NY, USA: Springer (2019). 61–9p. 10.1007/978-3-030-33642-4_4 PMC7486010 32924031

[59] Sun Y Shi H Zhang S Wang P Zhao W Zhou X. Accurate and rapid CT image segmentation of the eyes and surrounding organs for precise radiotherapy. Med Phys (2019) 46(5):2214–22. doi: 10.1002/mp.13463 30815885

[60] Tong N Gou S Yang S Cao M Sheng K. Shape constrained fully convolutional DenseNet with adversarial training for multiorgan segmentation on head and neck CT and low-field MR images. Med Phys (2019) 46(6):2669–82. doi: 10.1002/mp.13553 PMC6581189 31002188

[61] Xue Y Tang H Qiao Z Gong G Yin Y Qian Z. Shape-aware organ segmentation by predicting signed distance maps, in: Proceedings of the AAAI Conference on Artificial Intelligence. (2020) (Washington DC: AAAI). doi: 10.1609/aaai.v34i07.6946

[62] Jiang J Sharif E Um H Berry S Veeraraghavan H. Local block-wise self attention for normal organ segmentation [Internet]. arXiv [pre-print] (2019) 1–9. doi: 10.48550/arXiv.1909.05054

[63] Anand A Beltran CJ Brooke MD Buroker JR DeWees TA Foote RL. Study design: Validation of clinical acceptability of deep-learning-based automated segmentation of organs-at-risk for head-and-neck radiotherapy treatment planning. medRxiv (2021) 1–25. doi: 10.1101/2021.12.07.21266421 PMC10115982 37091160

[64] Taha AA Hanbury A. Metrics for evaluating 3D medical image segmentation: analysis, selection, and tool. BMC Med Imag (2015) 15(1):1–28. doi: 10.1186/s12880-015-0068-x PMC4533825 26263899

[65] Vaassen F Hazelaar C Vaniqui A Gooding M van der Heyden B Canters R. Evaluation of measures for assessing time-saving of automatic organ-at-risk segmentation in radiotherapy. Phys Imaging Radiat Oncol (2020) 13:1–6. doi: 10.1016/j.phro.2019.12.001 33458300 PMC7807544

[66] Moltz JH Hänsch A Lassen-Schmidt B Haas B Genghi A Schreier J. Learning a loss function for segmentation: A feasibility study, in: 2020 IEEE 17th International Symposium on Biomedical Imaging (New York, NY, USA: IEEE). (2020) 3–7pp.

[67] Nelms BE Robinson G Markham J Velasco K Boyd S Narayan S. Variation in external beam treatment plan quality: An inter-institutional study of planners and planning systems. Pract Radiat Oncol (2012) 2(4):296–305. doi: 10.1016/j.prro.2011.11.012 24674168

[68] Hart SG Staveland LE. Development of NASA-TLX (Task load index): Results of empirical and theoretical research. In: Hancock PA Meshkati N, editors. Advances in psychology (Amsterdam, Netherlands: Elsevier Science Publishing) (1988) 58, 139–83.

[69] Hart SG. Nasa-task load index (NASA-TLX); 20 years later. Proc Hum Factors Ergonom Soc Annu Meeting. (2006) 50(9):904–8. doi: 10.1177/154193120605000909

