# Supplementary material for: Validation of clinical acceptability of deep-learning-based automated segmentation of organs-at-risk for head-and-neck radiotherapy treatment planning
Source: Front Oncol. 2023 Apr 6;13:1137803. doi: 10.3389/fonc.2023.1137803 (PMC10115982; doi:10.3389/fonc.2023.1137803)
Supplement: Supplementary file 1 [file DataSheet_1.pdf]

Supplementary Material

Validation of clinical acceptability of deep-learning-based automated segmentation of organs-at-risk for head-and-neck radiotherapy treatment planning

J. John Lucido[1*], Todd A. DeWees[2], Todd R. Leavitt[2], Aman Anand[3], Chris J. Beltran[4], Mark D. Brooke[5], Justine R. Buroker[6], Robert L. Foote[1], Olivia R. Foss[7], Angela M. Gleason[7], Teresa L. Hodge[1], Cían O. Hughes[5], Ashley E. Hunzeker[1], Nadia N. Laack[1], Tamra K. Lenz[1], Michelle Livne[5], Megumi Morigami[5], Douglas J. Moseley[1], Lisa M. Undah1[1], Yojan Patel[5], Erik J. Tryggestad[1], Megan Z. Walker[5], Alexei Zverovitch[5], Samir H. Patel[3].

*** Correspondence:** J. John Lucido: lucido.joseph@mayo.edu

1 Gold Standard Contouring Process and Example

The gold standard (GS) was intended to be the best possible realization of the institutional guidelines and standards for organ-at-risk (OAR) contouring for a specific patient dataset. The institutional guidelines for head and neck (HN) OAR-contouring that have been adopted at this institution were primarily developed by two ROs (authors RLF and SHP) with expertise in HN cancer radiation therapy, along with an experienced dosimetrist (author AEH), with review and consensus approval by the departmental Head and Neck Cancer disease site group. The institutional standards are primarily based on international consensus guidelines,(1) but include additional OARs that are not part of those guidelines, such as the external auditory canals and mastoid sinuses (including air cells). In addition, there were institutional-specific modifications for grouping regional-anatomical structures as a single OAR (for instance, oral cavity and nasal cavity), and for separately delineating critical substructures of individual OARs (such as the cervical esophagus). A list of structure names and descriptions is given in Table S1.

The curation of the GS contours was performed on retrospectively identified datasets for patients using dedicated time without other clinical obligations. This ensured that the contouring could be performed without the time-pressure associated with getting patient treatment underway. The same two ROs who played a key role in defining the institutional standards worked with certified medical dosimetrists (CMDs) and medical dosimetry assistants (MDAs) to prepare the GS contours, all of whom had extensive experience with HN OAR contouring as part of their regular responsibilities. This meant that the team was able to spend substantially longer contouring time than is typical for routine clinical care. Figure S1 shows multiple CT slices with the GS contours below (for the same patient depicted in Fig. 2(b) in the main text).

Table S1: Organ-At-Risk Nomenclature

ID	Description	Abbreviations (if used)
brachial_plex_[l,r]	(Left or right) brachial plexus	bp
brain	Brain	
brain_stem	Brain stem	b_stem
carotid_artery_[l,r]	(Left or right) carotid artery	ca
cochlea_[l,r]	(Left or right) cochlea	
constrictors_p	Pharyngeal constrictor muscles	const_p
cord	Spinal cord	
crico_p_inlet	Cricopharyngeal inlet (upper esophageal sphincter)	cp_inlet
esophagus	Thoracic/abdominal esophagus	esoph
esophagus_cerv	Cervical esophagus	esoph_c
ext_aud_canal_[l,r]	(Left or right) external auditory canal	eac
eye_[l,r]	(Left or right) eyeball	
lacrimal_[l,r]	(Left or right) lacrimal gland	lac
larynx	Larynx (supraglottis, glottis, and subglottis)	
lens_[l,r]	(Left or right) lens	
lips	Lips	
lung_[l,r]	(Left or right) lung	
mandible	mandible	
mastoid_[l,r]	(Left or right) mastoid process	

nasal_cavity	Nasal cavity and paranasal sinuses	nasal_c
optic_nrv_[l,r]	(Left or right) optic nerve	oral_c
oral_cavity	Extended oral cavity	
parotid_[l,r]	(Left or right) parotid gland	
pituitary	Pituitary gland	
retina_[l,r]	(Left or right) retina	
semi_circ_canal_[l,r]	Semicircular canal	scc
sub_mandib_[l,r]	(Left or right) submandibular gland	smg
thyroid	Thyroid	

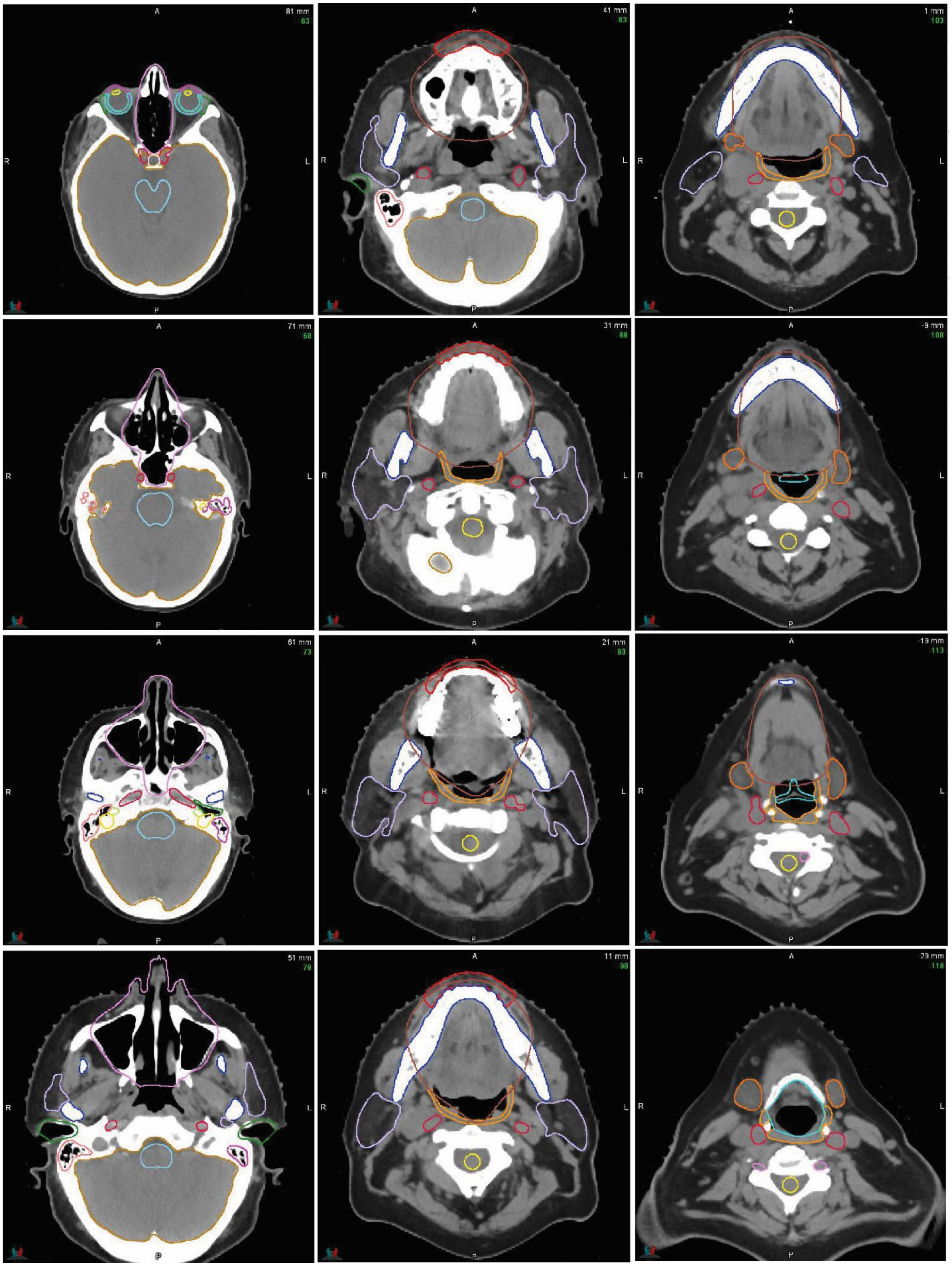
A
R
L
P
81 mm
63
41 mm
83
1 mm
103
71 mm
68
31 mm
88
-9 mm
108
61 mm
73
21 mm
93
-19 mm
113
51 mm
78
11 mm
98
-29 mm
118

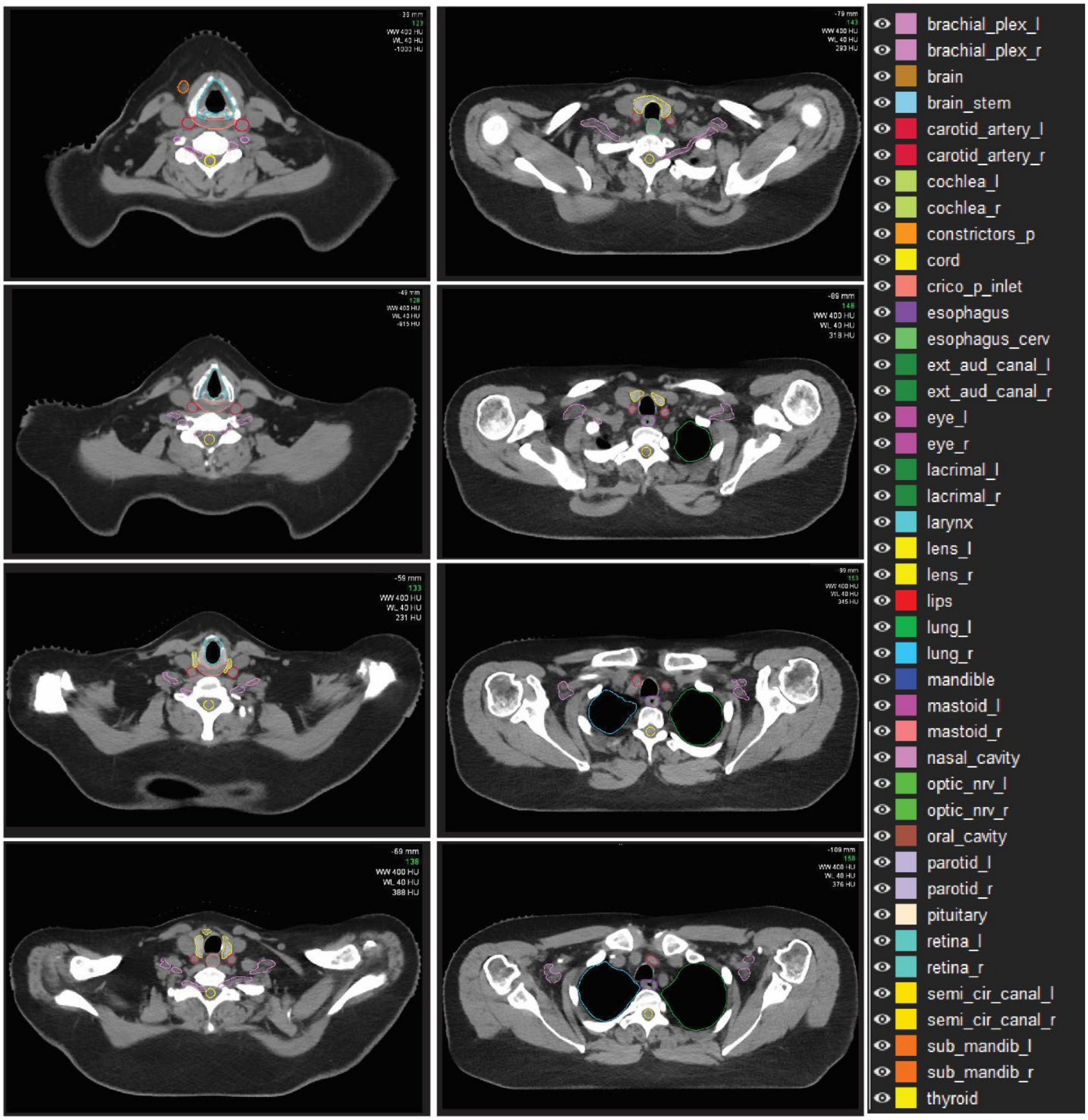

Figure S.1: Sample computed tomography image slices with GS contours represented for the same subject as in Figure 1(b) of the main text.

2 Deep Learning Model Architecture and Training

Model input: The inputs are subvolumes of 32 contiguous slices, with a stride of 4 slices at a time, where each slice is a 512x512 image. The output is a 32x512x512x42 tensor where 42 probability estimates are made for each voxel on whether a given organ is present in the voxel or not.

Preprocessing: The CTs are normalized from [-1024, 1024] Hounsfield Units to [-1, 1] to preserve the zero-point. During training only: 3D elastic deformation and affine transforms such as rotation and shearing are applied to provide augmentation and regularization to the model.

Architecture: We used a custom 3D U-Net architecture consisting of 6 convolutional blocks each in the encoder and decoder as shown in Figure E.1. Each residual block consists of 3 convolutional layers and a skip connection. The initial number of filters are 128, doubled every 2 blocks and the activations are down-sampled in the z axis every 2 blocks. Spatial dropout is applied for blocks 1-5 at a dropout rate of 0.25. The lateral connections from encoder to decoder are performed via a concatenation, as shown in Figure 1 in main text.

Loss: A hybrid combination of a region-based Dice loss and voxel-wise focal loss.(2) The focal loss parameterized with γ=2.0 and a relative weight of λ=20000. The Dice loss is computed in 2D for each slice and aggregated. The loss is further weighted by a novel class volume balancing term. Each class is weighted by a dynamic term:

$$w_c = \frac{1-\beta}{1-\beta^{V_c}}$$

where V_c is the exponential-moving average of the number of organ voxels in the training subvolumes for stable training statistics, and β = 0.999 in our experiments.

Training: The model is trained using a 8x8 TPUv3 pod using 16-way model parallelism and 8 replicas. The global batch size is 32 and the model is trained for 200K steps. We trained the model using the Adafactor(3) optimizer using an initial learning rate of 0.006 with exponential decay of 0.95 every 5000 steps.

Post-processing: The full volume probability mask is reconstructed by averaging probabilities from the predicted subvolume probability masks. These final probability masks are then vectorized to produce planar contours as final outputs. A marching squares method to find constant valued (0.5) contours from the probability mask. We used Catmull-Rom spline interpolation to introduce smoothness for visual aesthetics and small unphysical contours less than 5 mm^3 are removed.

3 Normalized Plan Quality Metric ScoreCard

The plan quality metric (PQM) framework introduced by Nelms, *et al.*,(4) provides a quantitative methodology for assessing the quality of a plan as a whole, including balancing the trade-offs between competing objectives. Clinical judgment and prioritization is built into the composite metric by assigning different relative weights to each of these objectives. The specific scorecard for the dose-volume constraints used to calculate the PQM for this study is shown below in Table S2, which represent the institutional guidelines for standard-fractionation head and neck radiation therapy. If the specified organ-at-risk (OAR) or target structure is present for that patient, the constraints for that structure are evaluated, and "points" are awarded for that constraint, and the total of these points is the PQM. In the PQM computed for this study, if the Ideal Objective was achieved for a given constraint, the maximum points were awarded. For the dose constraints that do not have Acceptable Objective listed in Table S2, no points were awarded if the plan did not achieve the Ideal Objective. If an Acceptable Objective is listed, no points were awarded if the value did not meet the Acceptable Objective, and if the value was between the Ideal and Acceptable objectives, the number of points awarded was linearly interpolated accordingly.

A single scorecard was used to evaluate all patient datasets in this study, while the number of target dose-levels and OARs present varied between the patients depending on the site of disease, disease staging, and past surgical history. Since this leads to variation in the number of points available for each patient, we computed a Normalized Plan Quality Metric (NPQM), which is the PQM reported as a percentage of the maximum total of points possible given the present target and OAR structures. The Scorecard takes into account clinical target volumes (CTVs) and planning target volumes (PTVs), and OAR nomenclature is given in Table S1.

Table S2: ScoreCard used to calculate the NPQM for HN RT plans, indicating the specific dose-volume constraint metrics, the objectives, and maximum possible points.

OAR	Metric	Ideal Objective	Acceptable Objective	Max Points
brachial_plex_l	D0.01cc	<63 Gy		5
brachial_plex_r	D0.01cc	<63 Gy		5
brain	D0.01cc	<56 Gy		5
brain_stem	D0.01cc	<54 Gy		5
cochlea_l	Mean Dose	<35 Gy		1
cochlea_r	Mean Dose	<35 Gy		1
constrictors_p	Mean Dose	<49 Gy	<59 Gy	2.5
cord	D0.01cc	<45 Gy		5

crico_p_inlet	Mean Dose	<49 Gy	<59 Gy	2
esophagus	Mean Dose	<29 Gy	<39 Gy	2.5
esophagus_cerv	Mean Dose	<29 Gy	<39 Gy	2.5
ext_aud_canal_l	D0.1cc	<60 Gy		1
ext_aud_canal_r	D0.1cc	<60 Gy		1
eye_l	D0.01cc	<50 Gy		1
eye_r	D0.01cc	<50 Gy		1
lacrimal_l	D0.03cc	<30 Gy		1
lacrimal_r	D0.03cc	<30 Gy		1
larynx-ptv	Mean Dose	<15 Gy	<25 Gy	3
lung_total	Mean Dose	<20 Gy		1
mandible	D0.03cc	<67.5 Gy	<75 Gy	1
nasal_cavity-ptv	Mean Dose	<35 Gy	<45 Gy	5
optic_nrv_l	D0.01cc	<50 Gy		5
optic_nrv_r	D0.01cc	<50 Gy		5
oral_cavity-ptv	Mean Dose	<20 Gy	<30 Gy	5
parotid_l-ptv	Mean Dose	<20 Gy	<30 Gy	2.5
parotid_r-ptv	Mean Dose	<20 Gy	<30 Gy	2.5
pituitary	Mean Dose	<10 Gy	<30 Gy	1
sub_mandib_l-ptv	Mean Dose	<30 Gy	<45 Gy	2.5
sub_mandib_r-ptv	Mean Dose	<30 Gy	<45 Gy	2.5

ptv_high	D1%	<108%	<112%	5
ptv_high	D95%	>100%	>95%	5
ptv_high	V100%	>98%	>90%	5
ptv_intermediate	D95%	>100%	>95%	2.5
ptv_intermediate	V100%	>98%	>90%	2.5
ptv_low	D95%	>100%	>95%	1
ptv_low	V100%	>98%	>90%	1
ctv_high	D95%	>100%	>95%	2.5
ctv_high	V100%	>98%	>90%	2.5
ctv_intermediate	D95%	>100%	>95%	2
ctv_intermediate	V100%	>98%	>90%	2
ctv_low	D95%	>100%	>95%	1
ctv_low	V100%	>98%	>90%	1

4 Additional Results

Table S3: Key Patient Characteristics for Hold Out Cohort

**Total**	**19 (100%)**
Disease Site	
Pharynx	6 (32%)
Lip and Oral Cavity	4 (21%)
Larynx	3 (16%)
Nasopharynx	3 (16%)
Salivary Glands	2 (11%)
Tonsil	1 (5%)
Treatment	
Definitive	10 (53%)
Adjuvant	9 (47%)
Age, Median (range) [years]	54.6 (22-81)
Gender Assigned at Birth	
Female	10 (53%)
Male	9 (47%)
Race	
Asian / Pacific Islander	2 (11%)
American Indian/Alaska Native	1 (5%)
Black, African American, African or American-born African	2 (11%)

White	11 (58%)
Other	2 (11%)
Unknown	1 (5%)
Ethnicity	
Hispanic or Latino	3 (16%)
Not Hispanic or Latino	15 (79%)
Unknown or Undisclosed	1 (5%)
Area Deprivation Index*	
5th Quintile (Most Deprived)	2 (11%)
4th Quintile	8 (42%)
3rd Quintile	4 (21%)
2nd Quintile	4 (21%)
1st Quintile (Least Deprived)	0 (0%)
Not Available	1 (5%)

* Area Deprivation Index is a census-tract based metric related to community socioeconomic indicators to measure the material and social conditions within a community.(5)

Table S4: Mean agreement with Gold Standard for all contours for metrics not included in Table 2 of main text.

Metric	Unrevised Deep Learning, DL (95%-CI)	Unrevised Deep Learning, DL+RO (95%-CI)	Manual, MDA+RO (95%-CI)
SDSC-1.5mm	0.93 (0.01)	0.91 (0.01)	0.86 (0.01)
SDSC-2mm	0.97 (0.00)	0.95 (0.01)	0.91 (0.01)
SDSC-3mm	0.98 (0.00)	0.97 (0.01)	0.94 (0.01)
APL-0mm [mm]	94.8 (7.4)	93.6 (6.7)	105.9 (7.4)
APL-1.5mm [mm]	19.1 (1.9)	21.6 (2.2)	25.5 (2.5)
APL-2mm [mm]	11.1 (1.4)	13.0 (1.7)	16.3 (2.0)
APL-3mm [mm]	7.3 (1.0)	9.0 (1.3)	11.7 (1.6)
APL-5mm [mm]	3.8 (0.6)	5.4 (1.0)	7.2 (1.2)
APL-10mm [mm]	1.6 (0.3)	2.7 (0.6)	3.5 (0.8)
CDC-1mm [mm]	0.77 (0.01)	0.73 (0.01)	0.64 (0.02)
CDC-1.5mm [mm]	0.85 (0.01)	0.82 (0.01)	0.74 (0.01)
CDC-2mm [mm]	0.89 (0.01)	0.86 (0.01)	0.79 (0.01)
CDC-3mm [mm]	0.92 (0.01)	0.89 (0.01)	0.84 (0.01)
CDC-5mm [mm]	0.93 (0.01)	0.91 (0.01)	0.87 (0.01)
CDC-10mm [mm]	0.94 (0.01)	0.93 (0.01)	0.89 (0.01)

Abbreviations: DL = unrevised deep-learning model contours; DL+RO = radiation oncologist-revised DL contours; MDA+RO = contours after revision by radiation oncologist of the medical dosimetry assistant's initial contours;95%-CI = 95%-confidence interval; SDSC = surface Dice similarity coefficient; APL= added path length.

Table S5: Mean of Key Geometric Similarity Metrics By Individual OAR *

OAR	VDSC (95-%CI)			HD95% (95-%CI) [mm]			APL-1mm (95-%CI) [mm]			SDSC-1mm (95-%CI)		
	DL	DL + RO	MDA + RO	DL	DL + RO	MDA + RO	DL	DL + RO	MDA + RO	DL	DL + RO	MDA + RO
brain	0.99 (0.00)	0.99 (0.00)	**0.99 (0.00)**	1.1 (0.2)	1.4 (0.3)	1.2 (0.2)	**19.0 (1.8)**	21.6 (2.0)	29.1 (7.9)	**0.93 (0.01)**	0.92 (0.01)	0.91 (0.03)
lung_l	0.98 (0.00)	0.98 (0.00)	**0.98 (0.00)**	1.7 (0.3)	1.9 (0.4)	2.1 (0.5)	**38.3 (4.7)**	49.2 (5.9)	54.1 (7.5)	**0.86 (0.02)**	0.84 (0.03)	0.82 (0.03)
lung_r	0.98 (0.00)	0.98 (0.00)	0.98 (0.00)	1.8 (0.3)	2.4 (0.6)	2.4 (0.6)	**40.0 (5.9)**	55.9 (9.2)	58.2 (7.2)	**0.86 (0.03)**	0.83 (0.03)	0.82 (0.03)
oral_c	0.95 (0.01)	0.92 (0.04)	0.86 (0.05)	**3.3 (0.7)**	5.9 (2.1)	6.9 (1.9)	130.6 (6.7)	143.6 (8.3)	153.7 (10.6)	**0.57 (0.04)**	0.50 (0.07)	0.37 (0.09)
nasal_c	0.95 (0.01)	0.94 (0.01)	**0.86 (0.04)**	**2.8 (0.4)**	4.1 (1.6)	6.7 (2.7)	82.1 (5.8)	87.1 (6.3)	100.9 (6.8)	**0.68 (0.03)**	0.64 (0.05)	**0.52 (0.08)**
mandible	0.96 (0.01)	0.96 (0.01)	**0.95 (0.01)**	0.9 (0.5)	1.2 (0.5)	1.8 (1.0)	4.7 (0.9)	5.7 (1.0)	9.4 (1.8)	**0.95 (0.01)**	0.94 (0.01)	**0.90 (0.02)**
esoph	0.86 (0.02)	0.86 (0.02)	**0.80 (0.03)**	4.0 (1.2)	4.9 (1.8)	5.7 (1.8)	11.5 (1.0)	12.1 (1.1)	13.4 (1.3)	0.73 (0.04)	0.72 (0.04)	0.65 (0.06)
parotid_l	0.89 (0.01)	0.89 (0.01)	**0.86 (0.01)**	3.5 (0.7)	3.6 (0.7)	4.6 (1.5)	37.4 (2.6)	38.2 (2.7)	33.6 (3.3)	0.69 (0.04)	0.68 (0.04)	0.63 (0.06)
parotid_r	0.89 (0.01)	0.89 (0.01)	**0.85 (0.02)**	3.4 (0.6)	3.8 (0.8)	4.0 (1.2)	**34.5 (2.1)**	35.4 (2.2)	32.5 (3.0)	0.68 (0.04)	0.68 (0.04)	0.63 (0.06)
lips	0.84 (0.02)	0.83 (0.02)	**0.74 (0.02)**	**4.0 (0.9)**	4.4 (0.9)	**6.4 (1.8)**	42.1 (3.6)	43.9 (3.8)	**69.7 (5.9)**	0.66 (0.04)	0.63 (0.05)	**0.53 (0.07)**
larynx	0.92 (0.01)	0.89 (0.04)	**0.80 (0.04)**	**2.1 (0.3)**	3.0 (0.8)	3.9 (1.0)	**18.3 (1.3)**	28.0 (4.6)	38.4 (6.0)	**0.78 (0.04)**	0.68 (0.10)	0.56 (0.09)
cord	0.86 (0.01)	0.85 (0.01)	**0.75 (0.05)**	1.4 (0.1)	1.5 (0.2)	**49.5 (36.4)**	5.7 (0.4)	7.4 (1.1)	9.8 (1.5)	0.76 (0.03)	0.71 (0.06)	0.61 (0.08)
b_stem	0.90 (0.01)	0.89 (0.01)	0.87 (0.01)	**2.7 (0.4)**	3.2 (0.5)	11.8 (15.4)	25.9 (1.4)	25.3 (1.3)	27.3 (2.3)	0.64 (0.04)	0.63 (0.03)	0.60 (0.06)

const_p	0.74 (0.05)	0.73 (0.05)	**0.59 (0.04)**	2.8 (0.6)	3.9 (1.4)	5.2 (1.0)	**34.5 (2.6)**	39.7 (3.2)	38.3 (3.2)	0.71 (0.04)	0.69 (0.05)	**0.57 (0.06)**
bp_l	0.75 (0.05)	0.71 (0.06)	**0.49 (0.08)**	2.9 (0.8)	5.0 (3.8)	**14.9 (6.2)**	20.4 (1.7)	21.9 (2.1)	**32.3 (2.3)**	0.73 (0.06)	0.70 (0.07)	**0.49 (0.08)**
bp_r	0.74 (0.05)	0.71 (0.06)	**0.48 (0.06)**	3.1 (0.7)	4.8 (2.9)	**15.9 (7.1)**	21.3 (1.8)	23.1 (2.2)	**32.9 (2.1)**	0.72 (0.06)	0.69 (0.07)	**0.49 (0.08)**
thyroid	0.91 (0.01)	0.91 (0.01)	**0.87 (0.02)**	1.8 (0.6)	2.0 (0.7)	1.9 (0.5)	9.7 (0.9)	10.4 (1.1)	12.2 (1.7)	0.86 (0.02)	0.86 (0.02)	0.82 (0.04)
mastoid_l	0.89 (0.06)	0.89 (0.07)	**0.85 (0.06)**	2.5 (2.2)	3.9 (2.3)	3.6 (2.6)	10.0 (1.2)	11.7 (1.5)	**17.3 (2.1)**	0.86 (0.06)	0.83 (0.08)	**0.77 (0.09)**
mastoid_r	0.89 (0.05)	0.88 (0.06)	0.86 (0.04)	2.7 (2.1)	3.6 (2.3)	4.3 (2.3)	10.4 (1.3)	11.0 (1.1)	**19.0 (2.4)**	0.85 (0.06)	0.84 (0.07)	**0.76 (0.08)**
eye_l	**0.95 (0.01)**	0.95 (0.01)	**0.93 (0.01)**	1.4 (0.1)	1.4 (0.1)	1.5 (0.2)	5.7 (0.9)	6.5 (0.9)	11.4 (2.8)	0.89 (0.02)	0.88 (0.02)	0.83 (0.05)
eye_r	0.95 (0.01)	0.95 (0.01)	**0.93 (0.02)**	1.4 (0.1)	1.4 (0.1)	1.7 (0.3)	6.6 (1.0)	7.8 (1.1)	11.2 (2.1)	0.88 (0.03)	0.87 (0.03)	0.82 (0.05)
smg_l	0.89 (0.02)	0.89 (0.02)	**0.87 (0.03)**	2.5 (1.2)	3.0 (1.5)	2.8 (1.3)	**10.8 (0.7)**	11.1 (0.7)	10.9 (1.2)	0.78 (0.04)	0.78 (0.05)	0.77 (0.04)
smg_r	0.90 (0.01)	0.90 (0.01)	**0.87 (0.03)**	1.8 (0.4)	1.8 (0.4)	3.3 (1.4)	**10.5 (0.8)**	10.7 (0.8)	10.0 (1.3)	0.80 (0.03)	0.79 (0.03)	0.75 (0.04)
cp_inlet	0.88 (0.01)	0.87 (0.01)	**0.75 (0.06)**	2.1 (0.4)	2.9 (1.1)	5.9 (3.9)	8.8 (1.0)	8.8 (1.0)	**14.1 (1.4)**	0.81 (0.03)	0.77 (0.06)	**0.61 (0.06)**
eac_l	0.87 (0.03)	0.86 (0.03)	**0.71 (0.04)**	1.8 (0.3)	2.7 (1.0)	3.8 (0.8)	19.0 (2.4)	23.4 (3.5)	34.7 (3.0)	0.79 (0.06)	0.76 (0.06)	**0.62 (0.06)**
eac_r	0.87 (0.03)	0.87 (0.03)	**0.70 (0.04)**	2.2 (0.3)	2.6 (0.8)	**4.2 (0.9)**	19.3 (2.7)	22.0 (3.7)	**34.5 (2.6)**	0.79 (0.05)	0.77 (0.07)	**0.63 (0.06)**
retina_l	0.82 (0.01)	0.79 (0.02)	**0.72 (0.05)**	1.4 (0.1)	1.9 (0.7)	2.6 (0.9)	**11.3 (1.1)**	14.8 (1.3)	18.6 (2.1)	**0.88 (0.02)**	0.85 (0.02)	0.79 (0.05)
retina_r	0.80 (0.02)	0.78 (0.02)	**0.69 (0.05)**	1.4 (0.1)	1.8 (0.6)	2.6 (1.0)	**12.3 (1.3)**	16.3 (1.3)	17.4 (2.0)	**0.87 (0.02)**	0.84 (0.02)	0.79 (0.05)

esoph_c	0.84 (0.04)	0.81 (0.08)	**0.70 (0.08)**	2.4 (0.5)	2.4 (0.5)	**4.2 (1.3)**	4.0 (0.7)	4.0 (0.8)	2.7 (0.5)	0.77 (0.06)	0.74 (0.09)	0.63 (0.06)
lacrimal_l	**0.73 (0.07)**	0.71 (0.06)	**0.55 (0.06)**	3.5 (1.4)	4.5 (1.4)	6.4 (1.6)	5.4 (0.6)	5.7 (0.7)	6.0 (0.8)	0.76 (0.07)	0.74 (0.06)	**0.62 (0.06)**
lacrimal_r	0.74 (0.05)	0.71 (0.05)	**0.55 (0.06)**	3.5 (1.2)	4.5 (1.3)	5.9 (1.5)	6.1 (0.8)	6.2 (0.9)	6.9 (1.1)	**0.76 (0.06)**	0.72 (0.07)	0.61 (0.06)
scc_l	0.85 (0.01)	0.79 (0.07)	**0.59 (0.09)**	1.7 (0.2)	2.4 (0.7)	2.6 (0.6)	**6.3 (0.7)**	7.6 (1.0)	7.5 (0.7)	0.83 (0.02)	0.73 (0.10)	0.61 (0.10)
scc_r	0.85 (0.02)	0.80 (0.06)	**0.59 (0.09)**	1.7 (0.2)	2.3 (0.7)	2.6 (0.6)	6.0 (0.9)	7.5 (1.0)	8.3 (1.0)	0.83 (0.03)	0.74 (0.09)	0.59 (0.11)
on_l	0.84 (0.02)	0.83 (0.02)	**0.76 (0.02)**	1.6 (0.2)	1.6 (0.2)	**2.4 (0.7)**	7.4 (1.1)	7.5 (1.1)	6.8 (0.7)	0.87 (0.03)	0.86 (0.03)	0.82 (0.03)
on_r	0.85 (0.02)	0.85 (0.02)	**0.78 (0.03)**	1.6 (0.2)	1.6 (0.2)	**2.4 (0.5)**	6.2 (0.7)	6.1 (0.8)	6.6 (1.0)	0.89 (0.02)	0.89 (0.02)	**0.84 (0.03)**
pituitary	0.83 (0.05)	0.83 (0.05)	**0.71 (0.06)**	1.8 (0.5)	1.9 (0.5)	2.0 (0.4)	4.5 (0.6)	4.7 (0.5)	**7.8 (1.0)**	0.82 (0.06)	0.81 (0.06)	0.72 (0.07)
cochlea_l	0.88 (0.02)	0.87 (0.02)	**0.79 (0.03)**	1.2 (0.3)	1.2 (0.3)	1.3 (0.3)	1.0 (0.2)	1.3 (0.4)	1.8 (0.6)	0.93 (0.01)	0.92 (0.02)	0.87 (0.04)
cochlea_r	0.86 (0.02)	0.86 (0.02)	**0.76 (0.05)**	1.2 (0.3)	1.5 (0.4)	1.4 (0.3)	0.9 (0.2)	1.1 (0.3)	1.8 (0.8)	0.93 (0.02)	0.90 (0.04)	0.85 (0.06)
lens_l	0.86 (0.02)	0.85 (0.03)	0.83 (0.04)	1.4 (0.3)	1.4 (0.3)	1.2 (0.3)	0.9 (0.5)	0.9 (0.5)	0.3 (0.1)	0.91 (0.03)	0.91 (0.03)	0.90 (0.04)
lens_r	0.86 (0.02)	0.86 (0.02)	0.85 (0.02)	1.4 (0.3)	1.4 (0.2)	1.2 (0.3)	0.6 (0.2)	0.6 (0.2)	0.4 (0.1)	0.91 (0.02)	0.92 (0.02)	0.93 (0.02)
ca_l	0.83 (0.02)	0.82 (0.02)	--	2.2 (1.4)	2.0 (0.4)	--	5.4 (0.6)	5.4 (0.6)	--	0.78 (1.6)	0.78 (1.6)	--
ca_r	0.81 (0.02)	0.82 (0.02)	--	3.0 (1.0)	2.6 (1.0)	--	6.3 (0.0)	6.0 (1.0)	--	0.76 (1.4)	0.76 (1.6)	--

Abbreviations: VDSC = volumetric Dice Similarity coefficient; HD95% = 95-percentile Hausdorff distance; APL = added path length; SDSC = surface Dice similarity coefficient; 95%-CI =95% confidence interval; DL = unrevised deep-learning model contours; DL+RO = radiation oncologis-

revised DL contours; MDA+RO = contours after revision by radiation oncologist of the medical dosimetry assistant’s initial contours.

*Bold indicates significant difference compared to DL+RO ($p<0.05$). Significance testing not performed for carotid arteries

Table S6: Mean Surface Dice Similarity Coefficient By Individual OAR with select values of τ

OAR	SDCS–1.5mm (95%-CI)			SDCS–2mm (95%-CI)			SDCS–3mm (95%-CI)		
	DL	DL + RO	MDA + RO	DL	DL + RO	MDA + RO	DL	DL + RO	MDA + RO
brain	0.99 (0.00)	0.99 (0.00)	0.97 (0.01)	1.00 (0.00)	1.00 (0.00)	0.99 (0.00)	1.00 (0.00)	1.00 (0.00)	1.00 (0.00)
lung_l	0.95 (0.01)	0.95 (0.02)	0.93 (0.02)	0.97 (0.01)	0.97 (0.01)	0.96 (0.01)	0.98 (0.01)	0.98 (0.01)	0.97 (0.01)
lung_r	0.95 (0.01)	0.94 (0.02)	0.93 (0.02)	0.97 (0.01)	0.97 (0.01)	0.96 (0.01)	0.98 (0.01)	0.98 (0.01)	0.97 (0.01)
oral_c	0.77 (0.04)	0.68 (0.08)	0.50 (0.08)	0.90 (0.03)	0.80 (0.08)	0.65 (0.08)	0.95 (0.02)	0.86 (0.08)	0.74 (0.08)
nasal_c	0.83 (0.03)	0.79 (0.05)	0.63 (0.07)	0.94 (0.02)	0.90 (0.04)	0.77 (0.07)	0.97 (0.02)	0.94 (0.03)	0.83 (0.07)
mandible	0.98 (0.01)	0.98 (0.01)	0.96 (0.01)	0.99 (0.01)	0.99 (0.01)	0.98 (0.01)	0.99 (0.01)	0.99 (0.01)	0.99 (0.01)
esoph	0.90 (0.02)	0.89 (0.03)	0.84 (0.03)	0.94 (0.02)	0.93 (0.02)	0.89 (0.02)	0.96 (0.01)	0.96 (0.02)	0.93 (0.02)
parotid_l	0.88 (0.03)	0.87 (0.02)	0.81 (0.03)	0.92 (0.02)	0.92 (0.02)	0.88 (0.02)	0.96 (0.01)	0.96 (0.01)	0.93 (0.02)
parotid_r	0.87 (0.02)	0.87 (0.02)	0.80 (0.03)	0.92 (0.02)	0.92 (0.02)	0.87 (0.03)	0.96 (0.01)	0.95 (0.01)	0.92 (0.03)
lips	0.81 (0.03)	0.79 (0.03)	0.72 (0.04)	0.90 (0.03)	0.89 (0.03)	0.82 (0.04)	0.94 (0.02)	0.93 (0.02)	0.88 (0.03)
larynx	0.92 (0.02)	0.82 (0.09)	0.74 (0.08)	0.97 (0.01)	0.89 (0.08)	0.83 (0.07)	0.99 (0.01)	0.93 (0.05)	0.90 (0.05)
cord	0.98 (0.01)	0.96 (0.02)	0.84 (0.06)	0.99 (0.00)	0.98 (0.01)	0.88 (0.06)	1.00 (0.00)	0.99 (0.00)	0.92 (0.06)
b_stem	0.84 (0.03)	0.83 (0.02)	0.81 (0.03)	0.94 (0.02)	0.92 (0.02)	0.90 (0.03)	0.97 (0.02)	0.96 (0.02)	0.95 (0.02)
const_p	0.90 (0.03)	0.88 (0.04)	0.80 (0.04)	0.94 (0.02)	0.92 (0.04)	0.85 (0.04)	0.97 (0.01)	0.95 (0.03)	0.90 (0.03)
bp_l	0.89 (0.03)	0.87 (0.04)	0.68 (0.07)	0.94 (0.02)	0.92 (0.03)	0.77 (0.06)	0.97 (0.01)	0.95 (0.02)	0.84 (0.05)

bp_r	0.88 (0.02)	0.86 (0.03)	0.68 (0.06)	0.94 (0.02)	0.92 (0.02)	0.76 (0.06)	0.97 (0.01)	0.95 (0.02)	0.83 (0.05)
thyroid	0.97 (0.01)	0.97 (0.01)	0.95 (0.02)	0.99 (0.01)	0.98 (0.01)	0.97 (0.02)	0.99 (0.00)	0.99 (0.01)	0.98 (0.01)
mastoid_l	0.95 (0.06)	0.93 (0.07)	0.90 (0.06)	0.96 (0.06)	0.95 (0.06)	0.94 (0.05)	0.97 (0.06)	0.96 (0.05)	0.96 (0.04)
mastoid_r	0.94 (0.06)	0.94 (0.06)	0.91 (0.03)	0.96 (0.06)	0.95 (0.06)	0.95 (0.02)	0.96 (0.06)	0.96 (0.05)	0.97 (0.01)
eye_l	0.98 (0.01)	0.97 (0.01)	0.96 (0.02)	1.00 (0.00)	1.00 (0.00)	0.99 (0.01)	1.00 (0.00)	1.00 (0.00)	1.00 (0.00)
eye_r	0.98 (0.01)	0.98 (0.01)	0.94 (0.04)	1.00 (0.00)	1.00 (0.00)	0.98 (0.02)	1.00 (0.00)	1.00 (0.00)	0.99 (0.01)
smg_l	0.94 (0.03)	0.94 (0.03)	0.91 (0.03)	0.97 (0.02)	0.96 (0.02)	0.94 (0.02)	0.98 (0.01)	0.98 (0.02)	0.97 (0.02)
smg_r	0.94 (0.02)	0.94 (0.02)	0.91 (0.04)	0.97 (0.02)	0.97 (0.02)	0.94 (0.03)	0.99 (0.01)	0.99 (0.01)	0.96 (0.02)
cp_inlet	0.94 (0.01)	0.90 (0.06)	0.83 (0.04)	0.98 (0.01)	0.95 (0.05)	0.90 (0.03)	0.99 (0.01)	0.96 (0.05)	0.94 (0.03)
ecc_l	0.93 (0.03)	0.90 (0.04)	0.77 (0.04)	0.98 (0.01)	0.95 (0.03)	0.88 (0.04)	0.99 (0.00)	0.97 (0.03)	0.93 (0.03)
ecc_r	0.92 (0.03)	0.91 (0.04)	0.75 (0.04)	0.97 (0.02)	0.95 (0.03)	0.86 (0.03)	0.99 (0.01)	0.98 (0.03)	0.91 (0.03)
retina_l	0.98 (0.01)	0.96 (0.02)	0.93 (0.02)	1.00 (0.00)	0.98 (0.02)	0.96 (0.02)	1.00 (0.00)	0.99 (0.02)	0.97 (0.02)
retina_r	0.97 (0.01)	0.96 (0.02)	0.92 (0.03)	0.99 (0.00)	0.98 (0.02)	0.95 (0.03)	1.00 (0.00)	0.99 (0.01)	0.97 (0.02)
esoph_c	0.84 (0.07)	0.80 (0.09)	0.74 (0.09)	0.95 (0.04)	0.94 (0.05)	0.86 (0.07)	0.96 (0.03)	0.95 (0.04)	0.90 (0.06)
lac_l	0.89 (0.06)	0.87 (0.05)	0.73 (0.06)	0.92 (0.05)	0.90 (0.04)	0.80 (0.06)	0.94 (0.05)	0.93 (0.03)	0.85 (0.06)
lac_r	0.90 (0.05)	0.86 (0.06)	0.75 (0.04)	0.93 (0.04)	0.90 (0.05)	0.82 (0.04)	0.96 (0.03)	0.93 (0.04)	0.88 (0.03)
scc_l	0.95 (0.01)	0.87 (0.09)	0.76 (0.08)	0.99 (0.01)	0.92 (0.07)	0.85 (0.07)	1.00 (0.00)	0.95 (0.05)	0.93 (0.05)

scc_r	0.95 (0.02)	0.88 (0.08)	0.77 (0.08)	0.99 (0.01)	0.94 (0.06)	0.87 (0.07)	1.00 (0.00)	0.96 (0.05)	0.94 (0.04)
on_l	0.96 (0.01)	0.95 (0.02)	0.92 (0.02)	1.00 (0.00)	0.99 (0.00)	0.98 (0.01)	1.00 (0.00)	1.00 (0.00)	0.99 (0.01)
on_r	0.96 (0.01)	0.97 (0.01)	0.92 (0.02)	1.00 (0.00)	1.00 (0.00)	0.98 (0.01)	1.00 (0.00)	1.00 (0.00)	0.99 (0.01)
pituitary	0.92 (0.04)	0.92 (0.04)	0.85 (0.07)	0.98 (0.02)	0.98 (0.02)	0.93 (0.05)	0.99 (0.02)	0.99 (0.02)	0.97 (0.03)
cochlea_l	0.99 (0.01)	0.99 (0.01)	0.97 (0.02)	1.00 (0.00)	1.00 (0.00)	0.99 (0.01)	1.00 (0.00)	1.00 (0.00)	1.00 (0.01)
cochlea_r	0.98 (0.01)	0.96 (0.03)	0.96 (0.03)	1.00 (0.00)	0.99 (0.01)	0.99 (0.02)	1.00 (0.00)	1.00 (0.00)	0.99 (0.01)
lens_l	0.99 (0.01)	0.99 (0.01)	0.98 (0.01)	1.00 (0.00)	1.00 (0.00)	1.00 (0.00)	1.00 (0.00)	1.00 (0.00)	1.00 (0.00)
lens_r	0.98 (0.01)	0.98 (0.01)	0.98 (0.01)	1.00 (0.00)	1.00 (0.00)	1.00 (0.00)	1.00 (0.00)	1.00 (0.00)	1.00 (0.00)

Abbreviations: SDSC = surface Dice similarity coefficient; 95%-CI =95% confidence interval; DL = unrevised deep-learning model contours; DL+RO = radiation oncologis-revised DL contours; MDA+RO = contours after revision by radiation oncologist of the medical dosimetry assistant’s initial contours.

Table S7 Mean By Individual OAR for Select Volumetric Measures

OAR	Precision (95%-CI)			Sensitivity (95%-CI)			Specificity (95%-CI)		
	DL	DL + RO	MDA + RO	DL	DL + RO	MDA + RO	DL	DL + RO	MDA + RO
brain	0.99 (0.00)	0.99 (0.00)	0.99 (0.00)	0.99 (0.00)	0.99 (0.00)	0.99 (0.00)	1.0 (0.0)	1.0 (0.0)	1.0 (0.0)
lung_l	0.98 (0.00)	0.97 (0.01)	0.97 (0.01)	0.99 (0.00)	0.99 (0.00)	0.99 (0.01)	1.0 (0.0)	1.0 (0.0)	1.0 (0.0)
lung_r	0.98 (0.00)	0.97 (0.01)	0.97 (0.01)	0.99 (0.00)	0.99 (0.00)	0.99 (0.00)	1.0 (0.0)	1.0 (0.0)	1.0 (0.0)
oral_c	0.95 (0.01)	0.95 (0.01)	0.85 (0.05)	0.94 (0.01)	0.89 (0.06)	0.95 (0.02)	1.0 (0.0)	1.0 (0.0)	1.0 (0.0)
nasal_c	0.95 (0.01)	0.94 (0.03)	0.85 (0.07)	0.94 (0.01)	0.93 (0.02)	0.95 (0.02)	1.0 (0.0)	1.0 (0.0)	1.0 (0.0)
mandible	0.97 (0.01)	0.96 (0.01)	0.94 (0.02)	0.98 (0.01)	0.97 (0.01)	0.97 (0.02)	1.0 (0.0)	1.0 (0.0)	1.0 (0.0)
esoph	0.82 (0.03)	0.81 (0.03)	0.79 (0.04)	0.91 (0.02)	0.91 (0.03)	0.88 (0.03)	1.0 (0.0)	1.0 (0.0)	1.0 (0.0)
parotid_l	0.88 (0.02)	0.88 (0.02)	0.82 (0.03)	0.91 (0.01)	0.90 (0.02)	0.95 (0.01)	1.0 (0.0)	1.0 (0.0)	1.0 (0.0)
parotid_r	0.88 (0.02)	0.88 (0.02)	0.82 (0.04)	0.90 (0.02)	0.90 (0.02)	0.95 (0.02)	1.0 (0.0)	1.0 (0.0)	1.0 (0.0)
lips	0.80 (0.03)	0.78 (0.03)	0.75 (0.06)	0.88 (0.05)	0.88 (0.05)	0.81 (0.07)	1.0 (0.0)	1.0 (0.0)	1.0 (0.0)
larynx	0.92 (0.02)	0.85 (0.06)	0.81 (0.04)	0.92 (0.02)	0.91 (0.03)	0.88 (0.07)	1.0 (0.0)	1.0 (0.0)	1.0 (0.0)
cord	0.86 (0.03)	0.81 (0.04)	0.80 (0.08)	0.89 (0.03)	0.90 (0.03)	0.80 (0.05)	1.0 (0.0)	1.0 (0.0)	1.0 (0.0)
b_stem	0.89 (0.02)	0.88 (0.02)	0.87 (0.02)	0.91 (0.02)	0.90 (0.02)	0.88 (0.03)	1.0 (0.0)	1.0 (0.0)	1.0 (0.0)

const_p	0.73 (0.06)	0.72 (0.06)	0.57 (0.06)	0.77 (0.04)	0.73 (0.06)	0.71 (0.05)	1.0 (0.0)	1.0 (0.0)	1.0 (0.0)
bp_l	0.71 (0.07)	0.69 (0.08)	0.48 (0.11)	0.81 (0.04)	0.77 (0.07)	0.58 (0.07)	1.0 (0.0)	1.0 (0.0)	1.0 (0.0)
bp_r	0.71 (0.07)	0.69 (0.07)	0.48 (0.10)	0.80 (0.04)	0.76 (0.06)	0.56 (0.07)	1.0 (0.0)	1.0 (0.0)	1.0 (0.0)
thyroid	0.90 (0.02)	0.90 (0.02)	0.85 (0.04)	0.93 (0.02)	0.92 (0.02)	0.95 (0.02)	1.0 (0.0)	1.0 (0.0)	1.0 (0.0)
mastoid_l	0.94 (0.02)	0.89 (0.07)	0.91 (0.04)	0.88 (0.07)	0.88 (0.07)	0.83 (0.10)	1.0 (0.0)	1.0 (0.0)	1.0 (0.0)
mastoid_r	0.94 (0.02)	0.89 (0.06)	0.94 (0.02)	0.87 (0.07)	0.88 (0.07)	0.80 (0.09)	1.0 (0.0)	1.0 (0.0)	1.0 (0.0)
eye_l	0.94 (0.01)	0.93 (0.01)	0.92 (0.02)	0.98 (0.01)	0.97 (0.01)	0.96 (0.02)	1.0 (0.0)	1.0 (0.0)	1.0 (0.0)
eye_r	0.93 (0.01)	0.93 (0.01)	0.92 (0.02)	0.98 (0.01)	0.97 (0.01)	0.97 (0.02)	1.0 (0.0)	1.0 (0.0)	1.0 (0.0)
smg_l	0.85 (0.04)	0.85 (0.04)	0.85 (0.05)	0.95 (0.01)	0.93 (0.02)	0.94 (0.02)	1.0 (0.0)	1.0 (0.0)	1.0 (0.0)
smg_r	0.86 (0.02)	0.86 (0.02)	0.83 (0.06)	0.95 (0.02)	0.95 (0.02)	0.93 (0.02)	1.0 (0.0)	1.0 (0.0)	1.0 (0.0)
crico_p_inlet	0.88 (0.02)	0.89 (0.02)	0.70 (0.06)	0.88 (0.03)	0.83 (0.08)	0.88 (0.05)	1.0 (0.0)	1.0 (0.0)	1.0 (0.0)
ext_aud_canal_l	0.85 (0.06)	0.85 (0.05)	0.72 (0.06)	0.91 (0.02)	0.86 (0.06)	0.85 (0.05)	1.0 (0.0)	1.0 (0.0)	1.0 (0.0)
ext_aud_canal_r	0.86 (0.06)	0.87 (0.06)	0.75 (0.07)	0.90 (0.03)	0.87 (0.06)	0.83 (0.06)	1.0 (0.0)	1.0 (0.0)	1.0 (0.0)
retina_l	0.77 (0.02)	0.75 (0.03)	0.67 (0.06)	0.89 (0.02)	0.85 (0.05)	0.80 (0.05)	1.0 (0.0)	1.0 (0.0)	1.0 (0.0)
retina_r	0.75 (0.02)	0.72 (0.03)	0.67 (0.05)	0.88 (0.02)	0.84 (0.04)	0.79 (0.05)	1.0 (0.0)	1.0 (0.0)	1.0 (0.0)

esoph_c	0.79 (0.06)	0.77 (0.09)	0.66 (0.07)	0.93 (0.04)	0.87 (0.10)	0.88 (0.06)	1.0 (0.0)	1.0 (0.0)	1.0 (0.0)
lac_l	0.76 (0.07)	0.75 (0.08)	0.54 (0.08)	0.77 (0.07)	0.73 (0.08)	0.76 (0.06)	1.0 (0.0)	1.0 (0.0)	1.0 (0.0)
lac_r	0.76 (0.07)	0.73 (0.09)	0.55 (0.08)	0.77 (0.06)	0.70 (0.08)	0.75 (0.07)	1.0 (0.0)	1.0 (0.0)	1.0 (0.0)
scc_l	0.89 (0.02)	0.90 (0.02)	0.54 (0.11)	0.82 (0.03)	0.71 (0.11)	0.94 (0.02)	1.0 (0.0)	1.0 (0.0)	1.0 (0.0)
scc_r	0.89 (0.03)	0.90 (0.03)	0.56 (0.11)	0.83 (0.04)	0.73 (0.10)	0.94 (0.03)	1.0 (0.0)	1.0 (0.0)	1.0 (0.0)
on_l	0.83 (0.02)	0.84 (0.02)	0.71 (0.05)	0.85 (0.04)	0.83 (0.04)	0.91 (0.03)	1.0 (0.0)	1.0 (0.0)	1.0 (0.0)
on_r	0.86 (0.02)	0.87 (0.01)	0.75 (0.05)	0.84 (0.03)	0.84 (0.03)	0.90 (0.04)	1.0 (0.0)	1.0 (0.0)	1.0 (0.0)
pituitary	0.86 (0.07)	0.85 (0.08)	0.75 (0.07)	0.84 (0.04)	0.84 (0.04)	0.83 (0.07)	1.0 (0.0)	1.0 (0.0)	1.0 (0.0)
cochlea_l	0.92 (0.03)	0.91 (0.04)	0.81 (0.06)	0.85 (0.02)	0.83 (0.03)	0.87 (0.05)	1.0 (0.0)	1.0 (0.0)	1.0 (0.0)
cochlea_r	0.90 (0.02)	0.90 (0.04)	0.76 (0.08)	0.86 (0.03)	0.83 (0.06)	0.90 (0.05)	1.0 (0.0)	1.0 (0.0)	1.0 (0.0)
lens_l	0.86 (0.04)	0.85 (0.04)	0.78 (0.05)	0.89 (0.05)	0.89 (0.05)	0.97 (0.01)	1.0 (0.0)	1.0 (0.0)	1.0 (0.0)
lens_r	0.87 (0.04)	0.88 (0.04)	0.82 (0.04)	0.88 (0.03)	0.87 (0.03)	0.95 (0.03)	1.0 (0.0)	1.0 (0.0)	1.0 (0.0)

Abbreviations: 95%-CI =95% confidence interval; DL = unrevised deep-learning model contours; DL+RO = radiation oncologist-revised DL contours; MDA+RO = contours after revision by radiation oncologist of the medical dosimetry assistant’s initial contours.

Table S8: Mean Added Path Length Using multiple tolerances, By OAR

OAR	APL - 2 mm [mm] (95%-CI)			APL - 3 mm [mm] (95%-CI)			APL - 5 mm [mm] (95%-CI)		
	DL	DL + RO	MDA + RO	DL	DL + RO	MDA + RO	DL	DL + RO	MDA + RO
brain	3.8 (0.3)	4.5 (0.4)	7.1 (1.9)	1.9 (0.3)	2.3 (0.3)	3.2 (0.8)	0.9 (0.2)	1.1 (0.3)	1.3 (0.3)
lung_l	16.1 (2.2)	19.7 (2.4)	22.2 (3.6)	10.6 (1.5)	12.7 (1.6)	14.0 (2.4)	6.2 (1.0)	7.2 (1.1)	7.7 (1.3)
lung_r	17.4 (3.0)	24.1 (4.1)	22.6 (3.6)	12.2 (2.2)	16.3 (2.7)	14.7 (2.4)	8.1 (1.6)	10.6 (2.0)	9.5 (1.5)
oral_c	63.5 (7.1)	83.1 (10.8)	111.1 (9.8)	32.4 (5.4)	53.2 (10.5)	79.3 (9.7)	11.8 (3.1)	27.9 (8.1)	43.6 (8.3)
nasal_c	36.0 (4.1)	40.0 (4.9)	55.9 (5.4)	18.1 (2.3)	22.1 (3.5)	35.3 (5.1)	6.5 (1.1)	9.1 (1.9)	18.7 (4.8)
mandible	3.0 (0.8)	3.8 (1.0)	2.8 (0.8)	2.3 (0.8)	3.1 (0.9)	2.0 (0.7)	1.5 (0.6)	2.3 (0.8)	1.1 (0.6)
esoph	3.7 (0.7)	3.7 (0.6)	4.5 (0.7)	1.7 (0.4)	1.6 (0.4)	1.8 (0.4)	0.6 (0.2)	0.5 (0.2)	0.4 (0.2)
parotid_l	16.2 (1.6)	16.3 (1.6)	13.3 (2.0)	9.8 (0.9)	10.0 (1.1)	6.4 (1.3)	4.7 (0.5)	5.0 (0.6)	2.3 (0.6)
parotid_r	14.3 (1.5)	14.9 (1.5)	12.3 (1.5)	8.6 (1.1)	9.2 (1.1)	5.5 (0.8)	4.3 (0.7)	4.7 (0.8)	1.5 (0.3)
lips	18.2 (1.7)	17.8 (1.6)	33.6 (3.7)	9.5 (1.1)	9.3 (1.0)	21.0 (3.2)	2.5 (0.5)	2.5 (0.5)	11.4 (2.5)
larynx	6.3 (0.8)	15.5 (4.4)	21.9 (5.7)	2.9 (0.5)	10.3 (3.4)	15.2 (4.8)	1.0 (0.3)	4.0 (1.5)	7.3 (2.5)
cord	0.4 (0.1)	0.9 (0.3)	1.9 (0.4)	0.0 (0.0)	0.1 (0.0)	0.2 (0.1)	0.0 (0.0)	0.0 (0.0)	0.0 (0.0)
b_stem	8.3 (0.9)	7.8 (0.8)	11.9 (1.6)	3.0 (0.6)	2.9 (0.6)	6.2 (1.2)	0.3 (0.2)	0.4 (0.2)	1.9 (0.6)
const_p	11.9 (1.6)	16.3 (2.7)	13.2 (2.2)	6.5 (1.1)	10.8 (2.4)	7.0 (1.6)	2.8 (0.6)	6.3 (1.9)	2.9 (0.9)

bp_l	8.1 (1.1)	9.6 (1.7)	18.7 (2.2)	4.3 (0.7)	5.7 (1.2)	12.4 (1.8)	2.0 (0.4)	3.0 (0.8)	6.3 (1.3)
bp_r	9.2 (1.1)	10.8 (1.6)	19.3 (2.1)	5.2 (0.7)	6.8 (1.3)	12.7 (1.8)	2.4 (0.5)	3.9 (0.9)	6.3 (1.1)
thyroid	2.3 (0.5)	2.6 (0.7)	2.2 (0.9)	1.4 (0.4)	1.7 (0.5)	1.0 (0.5)	0.9 (0.3)	1.0 (0.4)	0.4 (0.2)
mastoid_l	2.7 (0.6)	4.1 (1.2)	8.3 (1.4)	1.7 (0.5)	3.0 (1.0)	5.6 (1.1)	1.1 (0.3)	2.1 (0.9)	3.4 (0.9)
mastoid_r	3.0 (0.7)	3.1 (0.6)	9.3 (1.6)	1.9 (0.5)	1.9 (0.5)	6.4 (1.3)	1.0 (0.3)	1.1 (0.3)	3.9 (1.0)
eye_l	1.2 (0.3)	1.1 (0.3)	1.9 (1.0)	0.2 (0.1)	0.2 (0.1)	0.3 (0.2)	0.0 (0.0)	0.0 (0.0)	0.0 (0.0)
eye_r	1.2 (0.3)	1.2 (0.3)	2.4 (0.8)	0.2 (0.1)	0.2 (0.1)	0.6 (0.3)	0.0 (0.0)	0.0 (0.0)	0.0 (0.0)
smg_l	2.5 (0.5)	2.5 (0.4)	3.0 (0.5)	0.7 (0.2)	0.7 (0.2)	1.1 (0.3)	0.0 (0.0)	0.1 (0.1)	0.2 (0.1)
smg_r	2.6 (0.5)	2.6 (0.5)	3.0 (0.8)	0.9 (0.3)	0.9 (0.2)	1.2 (0.4)	0.1 (0.1)	0.1 (0.1)	0.1 (0.1)
crico_p_inlet	1.5 (0.4)	1.5 (0.4)	3.5 (0.6)	0.6 (0.2)	0.5 (0.2)	0.8 (0.2)	0.2 (0.1)	0.0 (0.0)	0.1 (0.1)
ext_aud_canal_l	6.2 (1.6)	10.2 (2.8)	15.0 (2.3)	1.8 (0.3)	5.9 (2.5)	7.5 (1.2)	0.4 (0.2)	4.1 (2.2)	2.8 (0.8)
ext_aud_canal_r	6.9 (1.6)	8.8 (2.6)	14.9 (1.9)	2.8 (0.9)	4.8 (2.3)	8.0 (1.2)	1.1 (0.7)	2.8 (2.0)	2.8 (0.9)
retina_l	3.6 (0.6)	4.4 (1.0)	5.0 (0.7)	1.8 (0.4)	2.4 (0.8)	2.6 (0.5)	0.1 (0.0)	0.6 (0.5)	0.6 (0.2)
retina_r	3.6 (0.4)	4.7 (0.8)	5.0 (0.7)	1.9 (0.3)	2.5 (0.7)	2.4 (0.4)	0.2 (0.1)	0.7 (0.4)	0.7 (0.2)
esoph_c	0.5 (0.2)	0.4 (0.2)	0.1 (0.1)	0.0 (0.0)	0.0 (0.0)	0.0 (0.0)	0.0 (0.0)	0.0 (0.0)	0.0 (0.0)
lac_l	1.5 (0.3)	1.7 (0.4)	1.9 (0.4)	0.6 (0.2)	0.8 (0.3)	0.9 (0.3)	0.1 (0.1)	0.2 (0.1)	0.3 (0.1)
lac_r	2.0 (0.4)	2.1 (0.5)	2.4 (0.6)	0.7 (0.2)	0.9 (0.4)	1.1 (0.4)	0.1 (0.1)	0.3 (0.2)	0.3 (0.2)
scc_l	2.0 (0.4)	3.5 (0.9)	2.9 (0.4)	0.7 (0.2)	2.1 (0.7)	1.2 (0.2)	0.0 (0.0)	0.8 (0.4)	0.0 (0.0)
scc_r	1.5 (0.4)	2.9 (0.7)	3.1 (0.5)	0.4 (0.1)	1.5 (0.5)	1.3 (0.3)	0.0 (0.0)	0.5 (0.3)	0.0 (0.0)

on_l	2.3 (0.7)	2.3 (0.7)	1.5 (0.5)	1.4 (0.6)	1.5 (0.6)	1.0 (0.4)	0.8 (0.5)	0.9 (0.5)	0.6 (0.3)
on_r	1.8 (0.5)	1.7 (0.5)	1.7 (0.7)	1.0 (0.3)	0.9 (0.4)	1.2 (0.6)	0.5 (0.3)	0.5 (0.3)	0.7 (0.4)
pituitary	0.8 (0.4)	0.4 (0.2)	2.4 (0.7)	0.3 (0.3)	0.0 (0.0)	1.1 (0.5)	0.2 (0.2)	0.0 (0.0)	0.3 (0.2)
cochlea_l	0.1 (0.1)	0.1 (0.1)	0.1 (0.1)	0.0 (0.0)	0.0 (0.0)	0.0 (0.0)	0.0 (0.0)	0.0 (0.0)	0.0 (0.0)
cochlea_r	0.2 (0.1)	0.2 (0.1)	0.3 (0.3)	0.0 (0.0)	0.0 (0.0)	0.0 (0.0)	0.0 (0.0)	0.0 (0.0)	0.0 (0.0)
lens_l	0.1 (0.1)	0.1 (0.1)	0.0 (0.0)	0.0 (0.0)	0.0 (0.0)	0.0 (0.0)	0.0 (0.0)	0.0 (0.0)	0.0 (0.0)
lens_r	0.0 (0.0)	0.0 (0.0)	0.0 (0.0)	0.0 (0.0)	0.0 (0.0)	0.0 (0.0)	0.0 (0.0)	0.0 (0.0)	0.0 (0.0)

Abbreviations: APL = added path length; 95%-CI =95% confidence interval; DL = unrevised deep-learning model contours; DL+RO = radiation oncologist-revised DL contours; MDA+RO = contours after revision by radiation oncologist of the medical dosimetry assistant's initial contours.

Table S9: Mean Contour Dice Coefficient By Organ-At-Risk

OAR	CDC - 1 mm (95%-CI)			CDC - 2 mm (95%-CI)			CDC - 3 mm (95%-CI)			CDC - 5 mm (95%-CI)		
	DL	DL + RO	MDA + RO	DL	DL + RO	MDA + RO	DL	DL + RO	MDA + RO	DL	DL + RO	MDA + RO
brain	0.92 (0.01)	0.92 (0.01)	0.89 (0.05)	0.97 (0.00)	0.97 (0.01)	0.96 (0.02)	0.98 (0.00)	0.98 (0.01)	0.97 (0.01)	0.99 (0.00)	0.98 (0.01)	0.98 (0.01)
lung_l	0.89 (0.03)	0.86 (0.03)	0.84 (0.04)	0.95 (0.01)	0.94 (0.01)	0.92 (0.02)	0.96 (0.01)	0.96 (0.01)	0.94 (0.02)	0.98 (0.01)	0.97 (0.01)	0.95 (0.02)
lung_r	0.89 (0.02)	0.86 (0.03)	0.85 (0.03)	0.94 (0.02)	0.93 (0.01)	0.93 (0.02)	0.95 (0.01)	0.95 (0.01)	0.95 (0.01)	0.96 (0.01)	0.96 (0.01)	0.96 (0.01)
oral_c	0.48 (0.04)	0.41 (0.06)	0.28 (0.09)	0.73 (0.05)	0.65 (0.09)	0.45 (0.10)	0.85 (0.04)	0.76 (0.09)	0.58 (0.10)	0.93 (0.02)	0.86 (0.07)	0.73 (0.09)
nasal_c	0.64 (0.05)	0.59 (0.06)	0.45 (0.08)	0.82 (0.03)	0.78 (0.05)	0.64 (0.08)	0.89 (0.02)	0.85 (0.05)	0.72 (0.08)	0.93 (0.02)	0.90 (0.04)	0.79 (0.08)
mandible	0.97 (0.01)	0.97 (0.01)	0.93 (0.02)	0.98 (0.01)	0.98 (0.01)	0.97 (0.01)	0.98 (0.01)	0.98 (0.01)	0.97 (0.01)	0.99 (0.01)	0.99 (0.01)	0.98 (0.01)
esoph	0.76 (0.04)	0.73 (0.05)	0.67 (0.06)	0.90 (0.03)	0.89 (0.03)	0.84 (0.04)	0.94 (0.02)	0.93 (0.02)	0.90 (0.02)	0.96 (0.01)	0.95 (0.02)	0.94 (0.02)
parotid_l	0.63 (0.05)	0.62 (0.05)	0.57 (0.07)	0.82 (0.04)	0.81 (0.04)	0.76 (0.05)	0.88 (0.03)	0.87 (0.03)	0.83 (0.04)	0.93 (0.02)	0.92 (0.02)	0.88 (0.03)
parotid_r	0.62 (0.04)	0.62 (0.04)	0.56 (0.07)	0.81 (0.03)	0.81 (0.03)	0.75 (0.05)	0.86 (0.03)	0.86 (0.03)	0.82 (0.05)	0.90 (0.02)	0.90 (0.02)	0.87 (0.04)
lips	0.63 (0.04)	0.60 (0.05)	0.46 (0.08)	0.78 (0.04)	0.76 (0.04)	0.68 (0.05)	0.84 (0.04)	0.82 (0.04)	0.76 (0.05)	0.89 (0.03)	0.87 (0.03)	0.82 (0.05)
larynx	0.77 (0.04)	0.66 (0.11)	0.55 (0.10)	0.90 (0.02)	0.80 (0.10)	0.74 (0.10)	0.94 (0.02)	0.85 (0.08)	0.81 (0.08)	0.96 (0.02)	0.91 (0.04)	0.90 (0.05)
cord	0.81 (0.03)	0.74 (0.07)	0.60 (0.10)	0.98 (0.01)	0.96 (0.02)	0.81 (0.10)	1.00 (0.00)	0.99 (0.01)	0.86 (0.10)	1.00 (0.00)	0.99 (0.00)	0.86 (0.10)
b_stem	0.65 (0.03)	0.62 (0.03)	0.62 (0.06)	0.86 (0.02)	0.84 (0.03)	0.81 (0.04)	0.92 (0.02)	0.89 (0.03)	0.87 (0.03)	0.95 (0.02)	0.92 (0.03)	0.92 (0.03)

const_p	0.70 (0.04)	0.68 (0.05)	0.56 (0.06)	0.86 (0.03)	0.85 (0.04)	0.76 (0.05)	0.90 (0.02)	0.89 (0.04)	0.82 (0.04)	0.93 (0.02)	0.92 (0.03)	0.88 (0.03)
bp_l	0.68 (0.06)	0.63 (0.09)	0.37 (0.07)	0.85 (0.04)	0.79 (0.09)	0.56 (0.09)	0.90 (0.03)	0.85 (0.08)	0.65 (0.08)	0.93 (0.02)	0.88 (0.07)	0.73 (0.07)
bp_r	0.67 (0.06)	0.61 (0.08)	0.36 (0.07)	0.84 (0.04)	0.79 (0.07)	0.56 (0.08)	0.89 (0.03)	0.84 (0.06)	0.65 (0.08)	0.92 (0.02)	0.88 (0.05)	0.73 (0.07)
thyroid	0.86 (0.03)	0.85 (0.04)	0.81 (0.06)	0.93 (0.03)	0.93 (0.03)	0.92 (0.03)	0.94 (0.03)	0.94 (0.03)	0.94 (0.03)	0.95 (0.03)	0.95 (0.03)	0.95 (0.03)
mastoid_l	0.85 (0.07)	0.82 (0.09)	0.75 (0.10)	0.93 (0.08)	0.91 (0.09)	0.86 (0.09)	0.94 (0.08)	0.93 (0.08)	0.89 (0.09)	0.95 (0.08)	0.94 (0.08)	0.91 (0.09)
mastoid_r	0.82 (0.07)	0.81 (0.08)	0.71 (0.10)	0.91 (0.08)	0.91 (0.08)	0.83 (0.09)	0.92 (0.08)	0.92 (0.08)	0.87 (0.09)	0.93 (0.08)	0.93 (0.07)	0.90 (0.09)
eye_l	0.84 (0.04)	0.81 (0.04)	0.75 (0.07)	0.93 (0.03)	0.92 (0.03)	0.91 (0.04)	0.96 (0.03)	0.95 (0.02)	0.94 (0.03)	0.97 (0.02)	0.96 (0.02)	0.94 (0.02)
eye_r	0.83 (0.04)	0.81 (0.04)	0.75 (0.06)	0.94 (0.02)	0.94 (0.02)	0.90 (0.04)	0.96 (0.02)	0.96 (0.02)	0.94 (0.02)	0.97 (0.02)	0.97 (0.02)	0.95 (0.02)
smg_l	0.74 (0.06)	0.72 (0.07)	0.72 (0.06)	0.90 (0.05)	0.88 (0.06)	0.87 (0.06)	0.93 (0.05)	0.92 (0.05)	0.91 (0.06)	0.95 (0.04)	0.93 (0.05)	0.93 (0.05)
smg_r	0.77 (0.03)	0.76 (0.03)	0.70 (0.06)	0.91 (0.02)	0.91 (0.02)	0.83 (0.06)	0.95 (0.02)	0.95 (0.02)	0.87 (0.05)	0.97 (0.02)	0.97 (0.02)	0.90 (0.05)
cp_inlet	0.83 (0.03)	0.78 (0.07)	0.62 (0.06)	0.92 (0.02)	0.88 (0.07)	0.77 (0.05)	0.94 (0.02)	0.89 (0.07)	0.82 (0.04)	0.94 (0.02)	0.89 (0.07)	0.84 (0.04)
ecc_l	0.66 (0.08)	0.62 (0.09)	0.42 (0.09)	0.82 (0.06)	0.79 (0.07)	0.66 (0.07)	0.88 (0.04)	0.85 (0.06)	0.75 (0.06)	0.90 (0.04)	0.87 (0.06)	0.81 (0.06)
ecc_r	0.69 (0.07)	0.66 (0.09)	0.44 (0.08)	0.85 (0.05)	0.82 (0.06)	0.68 (0.06)	0.91 (0.03)	0.88 (0.05)	0.76 (0.05)	0.94 (0.03)	0.91 (0.05)	0.83 (0.06)
retina_l	0.78 (0.03)	0.74 (0.04)	0.64 (0.06)	0.90 (0.03)	0.88 (0.04)	0.83 (0.04)	0.94 (0.02)	0.92 (0.03)	0.88 (0.04)	0.97 (0.02)	0.95 (0.03)	0.92 (0.03)
retina_r	0.76 (0.04)	0.72 (0.04)	0.65 (0.06)	0.90 (0.02)	0.89 (0.03)	0.84 (0.04)	0.94 (0.02)	0.93 (0.03)	0.89 (0.04)	0.97 (0.02)	0.96 (0.02)	0.93 (0.04)
esoph_c	0.76 (0.06)	0.72 (0.10)	0.63 (0.07)	0.84 (0.06)	0.80 (0.10)	0.69 (0.08)	0.85 (0.06)	0.80 (0.10)	0.69 (0.08)	0.85 (0.06)	0.81 (0.10)	0.69 (0.08)

lac_l	0.67 (0.08)	0.63 (0.08)	0.47 (0.09)	0.78 (0.08)	0.74 (0.08)	0.59 (0.08)	0.80 (0.08)	0.77 (0.07)	0.63 (0.08)	0.82 (0.08)	0.79 (0.07)	0.65 (0.08)
lac_r	0.69 (0.09)	0.63 (0.10)	0.47 (0.08)	0.81 (0.09)	0.74 (0.09)	0.61 (0.08)	0.84 (0.09)	0.77 (0.09)	0.66 (0.08)	0.85 (0.08)	0.79 (0.09)	0.68 (0.08)
scc_l	0.78 (0.03)	0.67 (0.11)	0.52 (0.11)	0.90 (0.03)	0.79 (0.11)	0.70 (0.11)	0.93 (0.03)	0.83 (0.11)	0.76 (0.11)	0.94 (0.03)	0.86 (0.09)	0.82 (0.09)
scc_r	0.78 (0.04)	0.67 (0.10)	0.50 (0.11)	0.90 (0.04)	0.80 (0.10)	0.70 (0.10)	0.93 (0.04)	0.83 (0.10)	0.76 (0.09)	0.94 (0.04)	0.85 (0.09)	0.81 (0.09)
on_l	0.80 (0.06)	0.79 (0.06)	0.72 (0.06)	0.90 (0.04)	0.89 (0.04)	0.83 (0.06)	0.91 (0.04)	0.90 (0.04)	0.84 (0.06)	0.93 (0.04)	0.92 (0.04)	0.85 (0.06)
on_r	0.77 (0.05)	0.79 (0.05)	0.73 (0.06)	0.84 (0.05)	0.87 (0.05)	0.81 (0.05)	0.86 (0.05)	0.88 (0.05)	0.83 (0.05)	0.87 (0.05)	0.89 (0.05)	0.84 (0.05)
pituitary	0.75 (0.08)	0.74 (0.08)	0.62 (0.08)	0.86 (0.07)	0.87 (0.07)	0.80 (0.07)	0.87 (0.07)	0.88 (0.06)	0.84 (0.06)	0.88 (0.07)	0.88 (0.06)	0.86 (0.06)
cochlea_l	0.93 (0.03)	0.91 (0.04)	0.83 (0.07)	0.96 (0.04)	0.96 (0.04)	0.91 (0.06)	0.97 (0.04)	0.96 (0.04)	0.92 (0.06)	0.97 (0.04)	0.96 (0.04)	0.92 (0.05)
cochlea_r	0.90 (0.05)	0.85 (0.08)	0.87 (0.08)	0.93 (0.05)	0.88 (0.07)	0.94 (0.05)	0.93 (0.04)	0.89 (0.06)	0.96 (0.05)	0.93 (0.04)	0.89 (0.06)	0.96 (0.05)
lens_l	0.90 (0.06)	0.89 (0.06)	0.92 (0.04)	0.93 (0.06)	0.93 (0.06)	0.96 (0.04)	0.94 (0.06)	0.94 (0.06)	0.96 (0.04)	0.94 (0.06)	0.94 (0.06)	0.96 (0.04)
lens_r	0.90 (0.05)	0.92 (0.05)	0.93 (0.05)	0.93 (0.05)	0.94 (0.05)	0.96 (0.05)	0.93 (0.05)	0.94 (0.05)	0.96 (0.05)	0.93 (0.05)	0.94 (0.05)	0.96 (0.05)

Abbreviations: CDC = Contour Dice Coefficient; 95%-CI =95% confidence interval; DL = unrevised deep-learning model contours; DL+RO = radiation oncologist-revised DL contours; MDA+RO = contours after revision by radiation oncologist of the medical dosimetry assistant's initial contours.

Table S10: Other Geometric Metrics, By Organ-At-Risk

OAR	Volume (cc) (95%-CI)			ΔVolume (cc) (95%-CI)			ΔVolume (%) (95%-CI)			ΔCentroid (mm) (95%-CI)		
	DL	DL + RO	MDA + RO	DL	DL + RO	MDA + RO	DL	DL + RO	MDA + RO	DL	DL + RO	MDA + RO
brain	1394.2 (77.6)	1396.5 (77.8)	1387.9 (79.6)	-0.8 (5.8)	1.5 (5.9)	-7.1 (10.1)	-0.1 (0.4)	0.1 (0.4)	-0.6 (0.7)	0.1 (0.0)	0.1 (0.0)	0.1 (0.0)
lung_l	1266.9 (179.4)	1273.0 (180.2)	1242.7 (171.6)	13.8 (11.3)	19.9 (12.1)	-10.4 (18.9)	1.2 (0.7)	1.7 (0.8)	-0.4 (1.5)	0.3 (0.2)	0.3 (0.2)	0.3 (0.1)
lung_r	1518.3 (231.0)	1526.7 (232.0)	1489.7 (225.3)	13.9 (12.5)	22.3 (14.0)	-14.8 (18.0)	1.0 (0.7)	1.6 (0.9)	-0.7 (1.2)	0.3 (0.2)	0.3 (0.2)	0.4 (0.1)
oral_c	308.5 (31.2)	296.7 (35.1)	265.1 (33.7)	-2.5 (6.0)	-14.3 (15.6)	-45.9 (22.4)	-0.8 (2.0)	-4.8 (5.5)	-15.0 (7.5)	0.8 (0.3)	1.5 (0.8)	1.6 (0.6)
nasal_c	174.5 (15.4)	176.0 (14.6)	152.3 (15.2)	-2.4 (3.2)	-0.9 (6.1)	-24.7 (17.7)	-1.2 (2.2)	0.1 (4.6)	-12.4 (7.7)	0.7 (0.2)	0.9 (0.4)	1.6 (0.7)
mandible	70.7 (7.5)	70.8 (7.6)	70.0 (8.4)	-0.2 (1.0)	-0.1 (0.9)	-0.9 (2.7)	-0.1 (1.4)	-0.1 (1.4)	-1.4 (3.6)	0.4 (0.2)	0.4 (0.2)	1.0 (0.4)
esoph	35.3 (4.5)	35.8 (4.6)	32.2 (4.9)	3.2 (1.0)	3.7 (1.0)	0.0 (2.1)	10.9 (3.3)	12.4 (3.3)	0.5 (7.1)	1.0 (0.3)	0.9 (0.3)	2.1 (0.7)
parotid_l	34.1 (4.3)	34.3 (4.4)	29.8 (4.1)	0.8 (0.8)	0.9 (1.0)	-3.5 (1.2)	2.8 (2.7)	3.3 (2.9)	-10.7 (3.1)	0.7 (0.2)	0.7 (0.1)	1.0 (0.2)
parotid_r	32.7 (5.0)	32.7 (5.0)	28.6 (4.4)	1.0 (1.2)	1.1 (1.2)	-3.1 (1.4)	2.9 (3.5)	3.1 (3.6)	-10.3 (5.6)	0.8 (0.2)	0.8 (0.2)	1.2 (0.4)
lips	32.1 (3.8)	33.1 (3.9)	30.4 (4.5)	3.0 (2.1)	4.0 (2.3)	1.3 (3.9)	11.0 (8.9)	14.6 (9.4)	6.2 (13.4)	1.4 (0.3)	1.4 (0.3)	2.1 (0.6)
larynx	27.3 (4.3)	29.4 (4.9)	25.9 (4.0)	-0.1 (0.8)	2.0 (2.4)	-1.5 (2.1)	0.1 (3.5)	7.8 (8.8)	-3.7 (8.2)	0.5 (0.1)	0.5 (0.1)	0.9 (0.3)
cord	25.0 (2.3)	26.7 (3.6)	26.7 (4.7)	1.0 (1.6)	2.8 (2.5)	2.7 (4.2)	5.6 (6.4)	11.8 (9.0)	12.3 (16.1)	2.8 (0.9)	2.4 (0.8)	8.5 (4.2)
b_stem	23.6 (1.4)	24.0 (1.3)	23.3 (1.5)	0.4 (0.7)	0.9 (0.8)	0.2 (1.0)	2.0 (3.2)	4.2 (3.8)	0.8 (4.2)	0.6 (0.2)	0.6 (0.2)	1.0 (0.5)
const_p	18.1 (1.9)	18.0 (1.9)	14.5 (1.9)	0.6 (1.0)	0.4 (1.0)	-3.0 (1.6)	6.4 (7.3)	5.3 (7.2)	-14.9 (8.1)	1.0 (0.2)	0.9 (0.3)	1.7 (0.7)
bp_l	15.3 (1.8)	15.1 (1.8)	13.1 (2.7)	1.3 (1.5)	1.2 (1.6)	-0.9 (2.6)	18.8 (20.3)	18.2 (21.3)	-0.6 (19.6)	3.4 (1.7)	5.1 (4.0)	14.9 (5.2)
bp_r	15.1 (2.3)	14.9 (2.1)	13.1 (2.3)	1.1 (1.2)	1.0 (1.4)	-0.9 (2.5)	15.9 (17.6)	15.6 (18.5)	2.9 (21.9)	4.4 (1.5)	5.4 (3.5)	15.3 (5.0)

thyroid	12.7 (1.6)	12.7 (1.6)	11.5 (1.6)	0.3 (0.4)	0.4 (0.4)	-0.8 (0.8)	3.3 (3.5)	4.0 (3.5)	-6.4 (6.4)	0.5 (0.1)	0.4 (0.1)	0.7 (0.2)
mastoid_l	10.1 (2.4)	10.4 (2.3)	11.2 (2.2)	-0.4 (0.2)	-0.1 (0.2)	0.7 (0.7)	-7.1 (7.8)	-0.4 (3.1)	22.6 (31.0)	0.9 (1.2)	0.6 (0.6)	1.0 (0.6)
mastoid_r	9.1 (2.1)	9.5 (2.0)	10.4 (1.9)	-0.4 (0.4)	0.0 (0.3)	0.9 (0.7)	-7.5 (7.7)	-0.2 (5.6)	17.2 (15.7)	0.9 (0.9)	0.8 (0.6)	0.9 (0.3)
eye_l	9.4 (0.6)	9.4 (0.6)	9.1 (0.7)	0.4 (0.1)	0.4 (0.1)	0.0 (0.4)	4.3 (1.4)	5.0 (1.7)	0.9 (4.6)	0.2 (0.1)	0.2 (0.1)	0.4 (0.1)
eye_r	9.3 (0.6)	9.4 (0.6)	8.8 (0.6)	0.5 (0.2)	0.5 (0.2)	0.0 (0.5)	5.3 (1.9)	6.2 (1.9)	0.6 (5.9)	0.2 (0.0)	0.2 (0.1)	0.4 (0.1)
smg_l	9.0 (1.5)	9.0 (1.5)	8.3 (1.6)	0.8 (0.3)	0.8 (0.3)	0.1 (0.4)	12.2 (5.9)	12.8 (6.0)	0.3 (6.3)	0.4 (0.2)	0.4 (0.2)	0.6 (0.1)
smg_r	8.8 (1.2)	8.8 (1.2)	8.0 (1.3)	0.8 (0.3)	0.8 (0.3)	0.0 (0.5)	10.3 (3.5)	10.7 (3.6)	-1.9 (7.3)	0.4 (0.2)	0.4 (0.2)	0.6 (0.3)
cp_inlet	6.7 (0.9)	6.6 (0.9)	5.1 (0.8)	0.0 (0.3)	-0.1 (0.4)	-1.6 (0.8)	-0.3 (4.7)	-2.1 (5.3)	-21.8 (11.1)	0.5 (0.1)	0.5 (0.1)	1.0 (0.3)
ecc_l	4.3 (0.4)	4.2 (0.4)	3.5 (0.4)	0.2 (0.2)	0.1 (0.3)	-0.6 (0.6)	10.4 (12.4)	9.3 (12.9)	-1.8 (23.0)	1.4 (0.3)	1.7 (0.5)	3.4 (0.8)
ecc_r	4.2 (0.4)	4.2 (0.4)	3.8 (0.5)	0.0 (0.3)	0.0 (0.3)	-0.4 (0.8)	7.5 (13.8)	7.5 (14.1)	6.2 (28.4)	1.2 (0.5)	1.2 (0.5)	3.6 (0.8)
retina_l	3.8 (0.2)	3.8 (0.4)	3.2 (0.3)	0.5 (0.1)	0.5 (0.3)	-0.1 (0.2)	14.4 (3.5)	15.7 (9.2)	-3.4 (6.9)	0.5 (0.1)	0.7 (0.4)	1.2 (0.5)
retina_r	3.8 (0.2)	3.8 (0.3)	3.2 (0.3)	0.6 (0.1)	0.6 (0.3)	0.0 (0.2)	17.9 (2.6)	19.2 (8.7)	0.3 (6.5)	0.5 (0.1)	0.6 (0.3)	1.3 (0.5)
esoph_c	2.3 (0.4)	2.3 (0.5)	1.7 (0.3)	0.3 (0.2)	0.3 (0.2)	-0.2 (0.3)	20.2 (13.1)	13.9 (12.3)	-9.4 (11.2)	0.5 (0.1)	0.6 (0.2)	1.2 (0.3)
lac_l	1.0 (0.1)	1.0 (0.1)	0.7 (0.1)	0.0 (0.1)	-0.1 (0.1)	-0.3 (0.2)	2.7 (16.7)	1.9 (18.4)	-25.2 (16.4)	1.0 (0.4)	1.0 (0.4)	2.0 (0.7)
lac_r	1.0 (0.1)	1.0 (0.1)	0.8 (0.1)	-0.1 (0.1)	-0.1 (0.1)	-0.3 (0.2)	3.5 (19.4)	-0.1 (20.1)	-17.4 (23.2)	1.0 (0.3)	1.1 (0.3)	2.0 (0.6)
scc_l	0.9 (0.1)	0.8 (0.1)	0.5 (0.1)	-0.1 (0.1)	-0.2 (0.1)	-0.5 (0.1)	-8.4 (5.2)	-17.2 (10.8)	-50.0 (11.7)	0.4 (0.1)	0.6 (0.3)	1.0 (0.4)
scc_r	0.9 (0.1)	0.8 (0.1)	0.5 (0.1)	-0.1 (0.1)	-0.1 (0.1)	-0.5 (0.1)	-6.5 (5.9)	-14.5 (10.8)	-46.5 (13.0)	0.4 (0.1)	0.5 (0.2)	0.9 (0.3)
on_l	1.0 (0.1)	1.0 (0.1)	0.8 (0.1)	0.0 (0.1)	0.0 (0.1)	-0.2 (0.1)	0.3 (5.2)	1.6 (5.1)	-21.9 (6.1)	1.1 (0.4)	1.0 (0.4)	1.7 (0.6)

on_r	0.9 (0.1)	0.9 (0.1)	0.8 (0.1)	0.0 (0.0)	0.0 (0.0)	-0.2 (0.1)	-3.0 (4.5)	-2.1 (4.7)	-16.3 (8.4)	0.8 (0.2)	0.7 (0.3)	1.7 (0.5)
pituitary	0.6 (0.1)	0.6 (0.1)	0.6 (0.1)	0.0 (0.1)	0.0 (0.1)	-0.1 (0.1)	3.5 (21.4)	7.9 (22.2)	5.6 (34.9)	0.4 (0.1)	0.3 (0.1)	0.5 (0.2)
cochlea_l	0.2 (0.0)	0.2 (0.0)	0.2 (0.0)	0.0 (0.0)	0.0 (0.0)	0.0 (0.0)	-9.5 (3.4)	-7.5 (5.5)	-8.7 (13.7)	0.3 (0.1)	0.3 (0.1)	0.4 (0.1)
cochlea_r	0.2 (0.0)	0.2 (0.0)	0.2 (0.0)	0.0 (0.0)	0.0 (0.0)	0.0 (0.0)	-6.3 (5.2)	-5.9 (4.7)	-14.9 (15.6)	0.3 (0.1)	0.3 (0.1)	0.4 (0.1)
lens_l	0.2 (0.0)	0.2 (0.0)	0.2 (0.0)	0.0 (0.0)	0.0 (0.0)	0.0 (0.0)	-1.0 (9.4)	0.3 (10.3)	-16.1 (8.3)	0.2 (0.1)	0.2 (0.1)	0.3 (0.1)
lens_r	0.2 (0.0)	0.2 (0.0)	0.2 (0.0)	0.0 (0.0)	0.0 (0.0)	0.0 (0.0)	-6.6 (5.6)	-3.9 (6.0)	-11.1 (7.5)	0.2 (0.1)	0.2 (0.0)	0.2 (0.1)

Abbreviations: ΔVolume = the mean difference in volume for each contour relative to the gold standard, ΔCentroid = the mean difference in the center of the contours relative to the gold standard. 95%-CI =95% confidence interval.

Table S11: Mean Absolute Value of Differences in Dose Metrics By Individual OAR*

OAR	|ΔD0.03cc| [Gy] (95%-CI)			|ΔDmean| [Gy] (95%-CI)		
	DL	DL + RO	MDA + RO	DL	DL + RO	MDA + RO
brain	0.7 (0.6)	0.7 (0.6)	1.0 (0.6)	0.0 (0.0)	0.0 (0.0)	0.0 (0.0)
lung_l	0.3 (0.1)	0.4 (0.2)	0.4 (0.3)	0.0 (0.0)	0.0 (0.0)	0.0 (0.0)
lung_r	0.3 (0.1)	0.3 (0.1)	0.5 (0.3)	0.0 (0.0)	0.0 (0.0)	0.0 (0.0)
oral_c	0.3 (0.4)	0.5 (0.5)	0.6 (0.6)	0.3 (0.1)	0.6 (0.4)	0.7 (0.3)
nasal_c	1.3 (1.1)	4.7 (6.8)	3.7 (2.0)	0.4 (0.1)	0.6 (0.4)	0.7 (0.4)
mandible	0.0 (0.0)	0.0 (0.0)	0.0 (0.0)	0.2 (0.1)	0.2 (0.1)	**0.4 (0.1)**
esoph	0.3 (0.2)	0.3 (0.2)	0.5 (0.3)	0.4 (0.2)	0.3 (0.2)	0.6 (0.3)
parotid_l	0.3 (0.2)	0.4 (0.2)	0.5 (0.2)	0.4 (0.2)	0.5 (0.2)	0.8 (0.4)
parotid_r	0.6 (0.4)	0.7 (0.5)	0.9 (0.4)	0.8 (0.4)	0.7 (0.4)	**1.0 (0.4)**
lips	1.3 (0.4)	1.4 (0.4)	1.9 (0.9)	0.7 (0.3)	0.7 (0.3)	**1.8 (0.7)**
larynx	0.5 (0.3)	0.7 (0.3)	**1.5 (0.7)**	0.6 (0.2)	1.2 (0.7)	1.1 (0.4)
cord	0.8 (0.4)	0.9 (0.4)	1.2 (0.4)	1.4 (0.5)	1.2 (0.4)	**3.9 (2.0)**
b_stem	0.6 (0.3)	0.9 (0.5)	1.5 (0.7)	0.6 (0.2)	0.5 (0.2)	0.8 (0.4)
const_p	0.4 (0.2)	0.4 (0.2)	0.5 (0.3)	0.5 (0.2)	0.5 (0.2)	**1.1 (0.5)**
bp_l	0.1 (0.1)	0.1 (0.1)	0.9 (0.8)	1.7 (1.3)	2.6 (2.1)	6.0 (2.8)
bp_r	0.4 (0.2)	0.3 (0.2)	1.9 (1.7)	1.5 (0.9)	2.1 (1.9)	**6.4 (2.4)**
thyroid	0.6 (1.0)	0.6 (1.0)	0.4 (0.7)	0.2 (0.1)	0.2 (0.1)	0.3 (0.1)
mastoid_l	1.5 (2.2)	2.0 (2.3)	1.5 (1.2)	0.4 (0.4)	0.5 (0.5)	0.7 (0.4)
mastoid_r	1.0 (1.3)	1.0 (1.3)	0.9 (0.9)	0.3 (0.2)	0.3 (0.2)	0.3 (0.2)

eye_l	0.2 (0.2)	0.5 (0.4)	0.8 (1.3)	0.0 (0.0)	0.1 (0.1)	0.1 (0.1)
eye_r	0.2 (0.2)	0.2 (0.2)	0.3 (0.2)	0.1 (0.1)	0.1 (0.1)	0.1 (0.0)
smg_l	0.1 (0.1)	0.1 (0.1)	0.3 (0.4)	0.5 (0.5)	0.5 (0.5)	0.6 (0.4)
smg_r	0.2 (0.3)	0.2 (0.3)	0.2 (0.1)	0.2 (0.1)	0.2 (0.1)	0.4 (0.3)
crico_p_inlet	1.1 (0.8)	1.7 (1.4)	**3.7 (1.7)**	0.5 (0.2)	0.6 (0.3)	**1.2 (0.5)**
ext_aud_canal_l	2.2 (1.9)	1.8 (1.2)	**3.6 (2.2)**	0.5 (0.2)	0.8 (0.5)	1.5 (0.9)
ext_aud_canal_r	0.8 (0.8)	1.1 (0.8)	**2.2 (1.5)**	0.3 (0.2)	0.3 (0.2)	**1.0 (0.5)**
retina_l	0.2 (0.2)	0.3 (0.2)	0.8 (1.3)	0.1 (0.1)	0.1 (0.1)	0.1 (0.1)
retina_r	0.2 (0.2)	0.6 (0.7)	0.3 (0.2)	0.1 (0.1)	0.2 (0.2)	0.2 (0.2)
esoph_c	0.5 (0.4)	0.8 (0.5)	1.8 (1.1)	0.4 (0.2)	0.5 (0.3)	0.8 (0.3)
lac_l	0.4 (0.3)	0.4 (0.3)	1.2 (1.2)	0.1 (0.1)	0.2 (0.1)	0.3 (0.2)
lac_r	0.7 (0.7)	0.7 (0.7)	0.9 (0.8)	0.2 (0.2)	0.4 (0.3)	0.5 (0.4)
scc_l	0.9 (0.5)	1.1 (0.6)	2.3 (1.1)	0.4 (0.2)	0.4 (0.2)	0.6 (0.3)
scc_r	1.1 (0.8)	1.4 (1.0)	1.8 (1.1)	0.4 (0.3)	0.4 (0.3)	0.5 (0.2)
on_l	0.3 (0.5)	0.4 (0.5)	**0.5 (0.7)**	0.2 (0.2)	0.2 (0.2)	0.2 (0.2)
on_r	0.6 (1.0)	0.6 (1.0)	1.1 (1.8)	0.3 (0.3)	0.2 (0.3)	0.4 (0.5)
pituitary	0.1 (0.1)	0.1 (0.1)	0.8 (1.4)	0.3 (0.3)	0.3 (0.3)	0.7 (0.9)
cochlea_l	0.6 (0.3)	0.5 (0.2)	1.4 (1.0)	0.4 (0.3)	0.2 (0.1)	**0.5 (0.2)**
cochlea_r	0.4 (0.3)	0.4 (0.3)	1.1 (0.9)	0.4 (0.3)	0.3 (0.2)	0.4 (0.3)
lens_l	0.1 (0.0)	0.1 (0.0)	0.1 (0.0)	0.0 (0.0)	0.0 (0.0)	0.0 (0.0)
lens_r	0.1 (0.1)	0.1 (0.1)	0.1 (0.1)	0.1 (0.0)	0.1 (0.0)	0.0 (0.0)
ca_l	0.5 (0.3)	0.7 (0.4)	--	1.3 (0.4)	1.6 (0.6)	--

ca_r	1.4 (0.9)	1.5 (1.0)	--	1.6 (0.8)	1.4 (0.5)	--

Abbreviations: 95%-CI =95% confidence interval; VDSC = volumetric Dice similarity coefficient; |ΔD0.03cc| = absolute value of the difference in minimum dose received by 0.03cc of the organ-at-risk; |ΔDmean| = absolute value of difference in mean dose received by the organ-at-risk; |ΔNPQM| = absolute value of the difference in normalized plan quality metric.

*Bold indicates significant difference compared to DL+RO ($p<0.05$). Significance testing not performed for carotid arteries

5 Comparison with previous work

To evaluate the performance of this model compared to other published atlas and machine-learning approaches, we refer to the extensive literature review included in the supplementary materials of Nikolov *et al.*(6) An additional literature search performed for this study for deep learning approaches to head and neck organ-at-risk (OAR) autosegmentation found seven additional studies that reported the mean Volumetric Dice Similarity Coefficients (VDSCs) for individual OARs for unrevised deep learning (DL) contours compared to manual delineations for at least one of the OARs included in this study that have been published in a peer-reviewed journal in PubMed. The mean VDSC for each OAR are shown in Table S12, along with the results from this study.

Most of the DL models reported multiple OARs with mean VDSC greater than 0.8, demonstrating very good agreement with the gold standard. However, none reported that level of performance for more than 16 individual OARs, and only 7 showed that for more than 10. In comparison, the DL model presented in this study achieved this for 36 OARs without any manual intervention. In addition, there were 10 OARs included in this study that were not reported in any of the studies in Table S12 or in Nikolov, *et a*l.(6): brachial_plex_l, brachial_plex_r, carotid_artery_l, carotid_artery_r, lips, mastoid_l, mastoid_r, nasal_cavity, retina_r, and retina_l. The inclusion of all of these OARs enables this deep learning model to be the only autosegmentation tool capable of accurately contouring the comprehensive set of OARs necessary for HN cancer treatment planning.

Table S12: Volumetric Dice Similarity Coefficients from Additional References

OAR	This Study*	Bunenberg [2020](7)[†]	van der Veen [2019](8)[‡]	Zhong [2021](9)	Fang [2021](10)[¦]	Amjad [2022](11)[¦][¶]	Thor [2021](12)[¶]	Wang [2021](13)	Costea [2022] [14][¦]
brain	0.99					0.98		0.99	
b_stem	0.90	0.78	0.95	0.8	0.83	0.90	0.82	0.86	0.85
cochlea_l	0.88		0.99						
cochlea_r	0.86		0.96						
const_p	0.74	0.60	0.84[§]					0.82	
cord	0.86		0.98	0.87	0.80	0.83		0.88	0.85
cp_inlet	0.88	0.60							
esoph	0.86		0.83			0.78			0.84
ecc_l	0.87					0.83			
ecc_r	0.87					0.82			
eye_l	0.95			0.93	0.86	0.91		0.94	0.91[#]
eye_r	0.95			0.93	0.86	0.91		0.94	
larynx	0.92		0.77[§]	0.84	0.75	0.85		0.90	
lens_l	0.86			0.71	0.68	0.74		0.89	
lens_r	0.86			0.78	0.66	0.79		0.91	
mandible	0.96	0.90	0.99			0.88	0.91	0.93	0.9
on_l	0.84			0.65	0.63	0.79		0.89	
on_r	0.85			0.69	0.64	0.79		0.91	
oral_c	0.95	0.86	0.93	0.93				0.92	0.85
parotid_l	0.89	0.83	0.96	0.92	0.78	0.82	0.83	0.82	
parotid_r	0.89	0.83	0.96	0.85	0.79	0.84	0.82	0.83	
pituitary	0.75							0.92	

smg_l	0.89	0.79	0.99			0.83	0.79		0.80#
smg_r	0.89	0.78	0.98			0.80	0.75		
thyroid	0.90	0.81						0.72	

Abbreviation: OAR = organ-at-risk.
* Data from unrevised deep learning (DL) model contours
† Corresponds to "global" VDSC
‡ This study compared deep learning model to two radiation oncologists; we entered the maximum value from the two presented.
§ Corresponds to the maximum over each of the subregions listed for this OAR.
¦ Corresponds to the maximum over all of the reported models.
¶ Estimated from graph
# Reported for both lateralities combined

6 Survey Results

If you have the option, how interested would you be in using Autogenerated OARs on your future H&N patient cases?

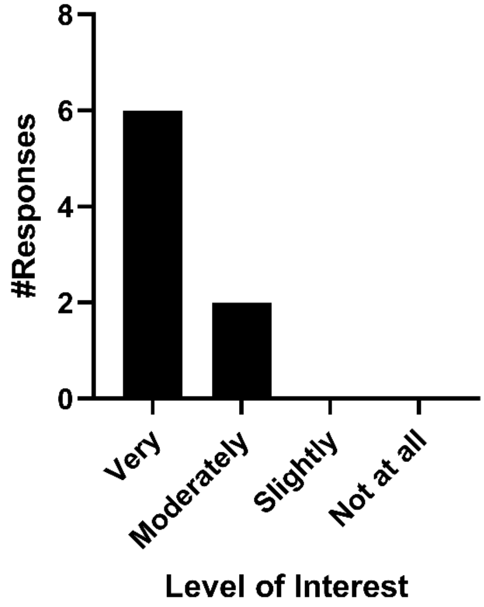

Would you want the model to automatically generate contours for all H&N cases?

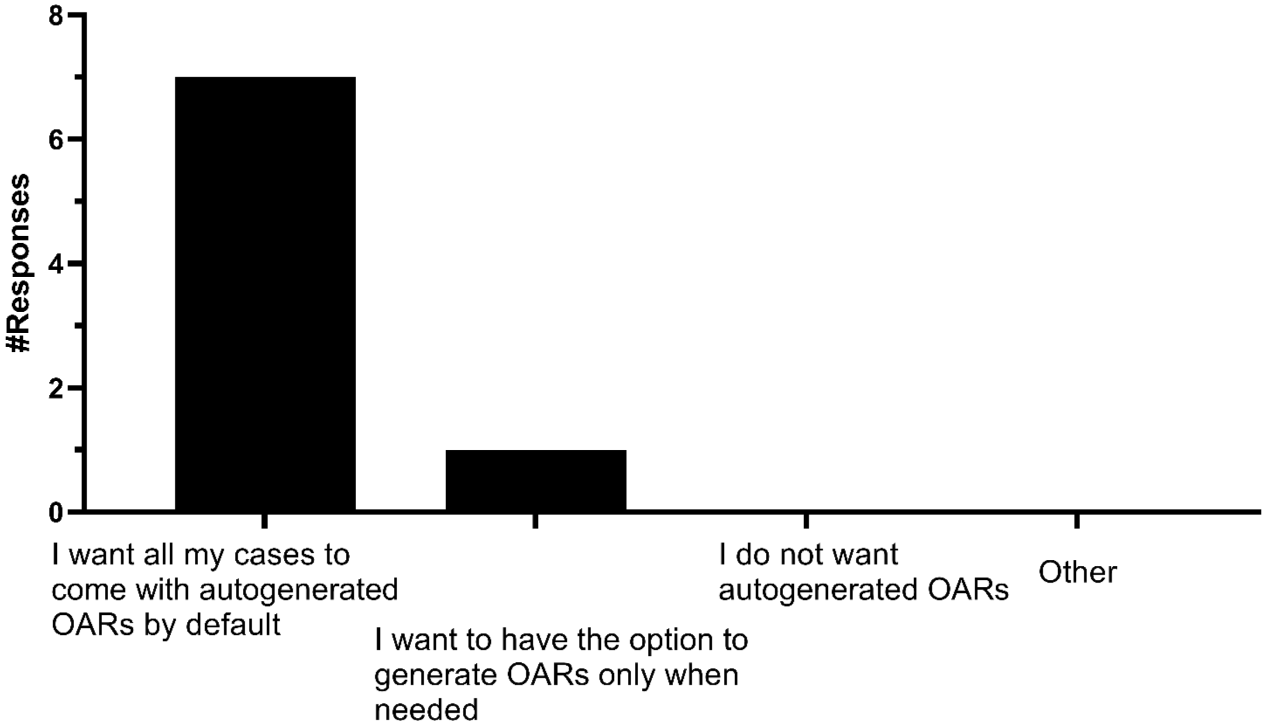

Sum of NASA-TLX Survey Results (lower score means better)

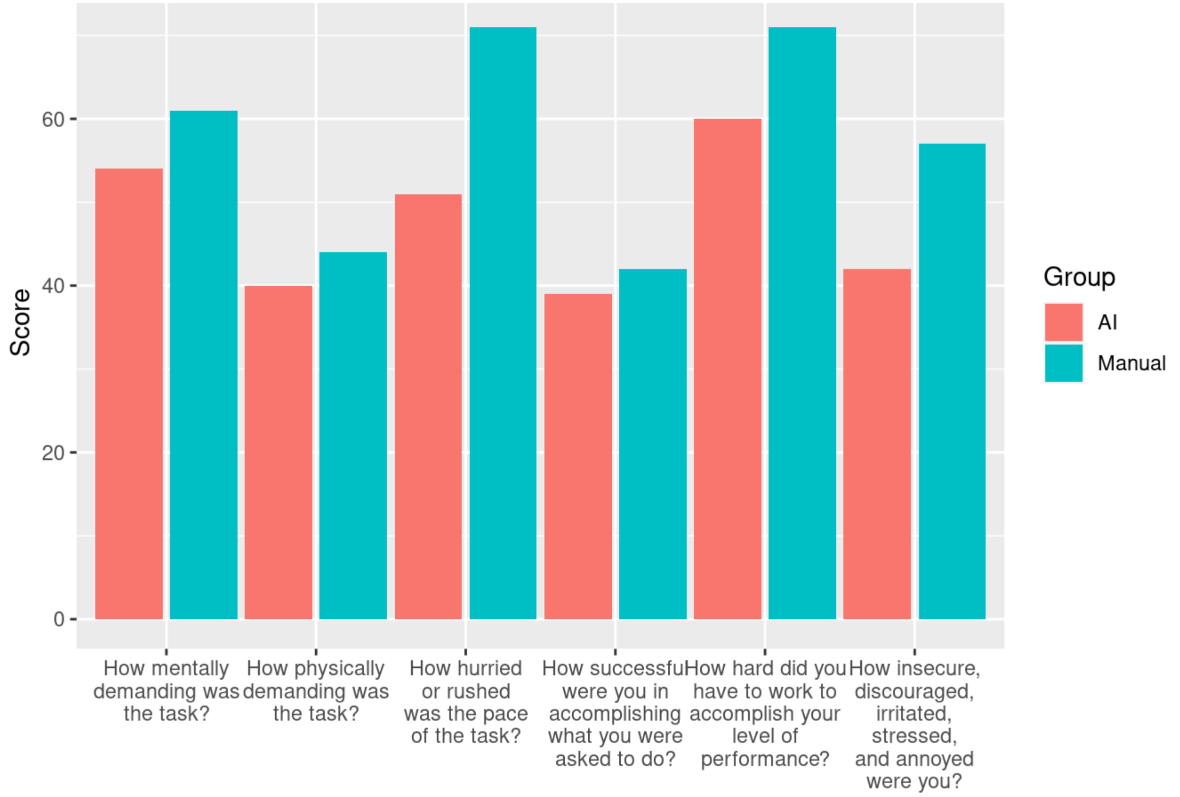

Count of Overall Contouring Errors Selected

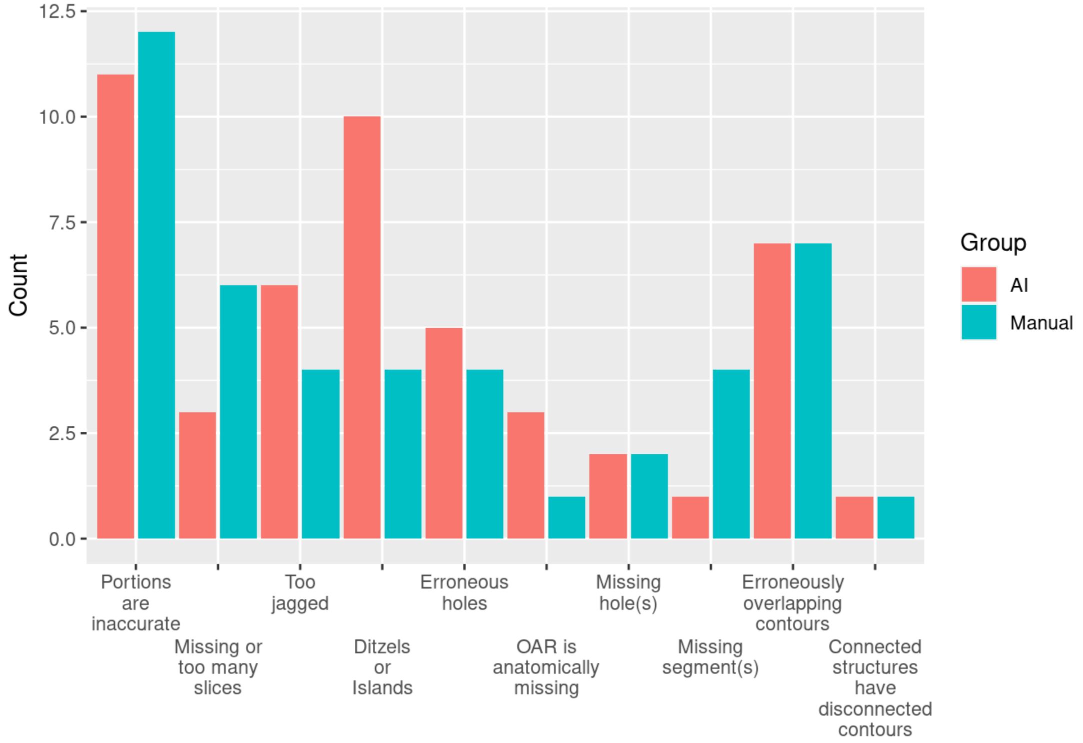

6.1 Severity of Modifications Required, per type of error

Portions are inaccurate

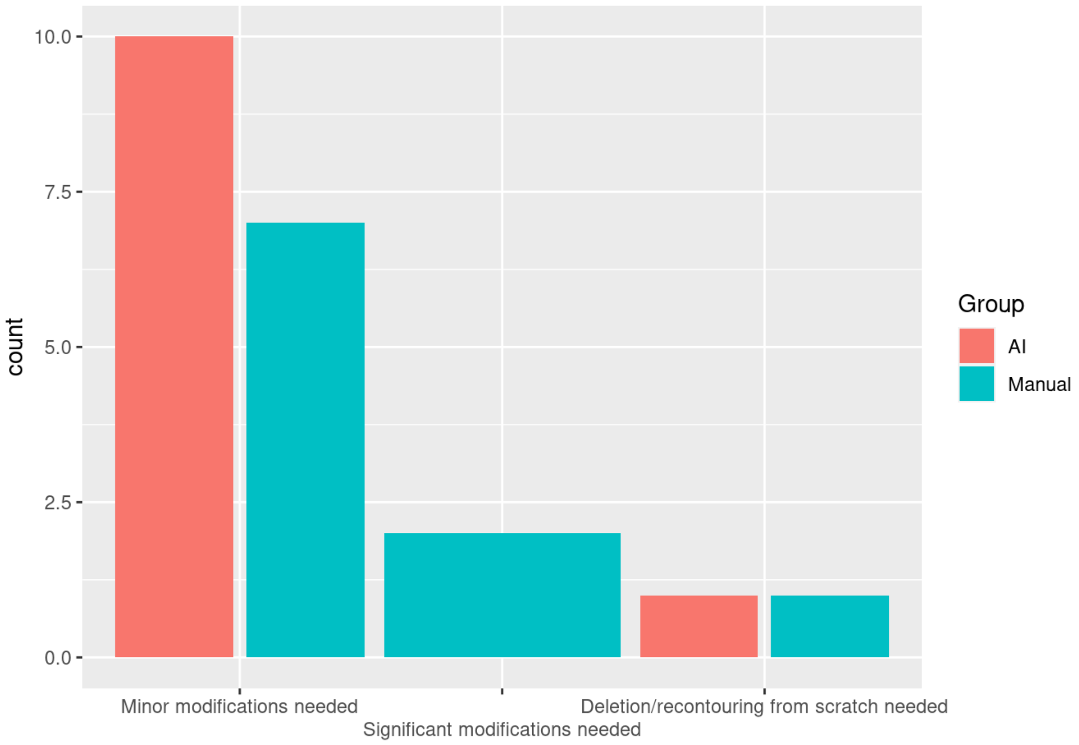

Missing or too many slices

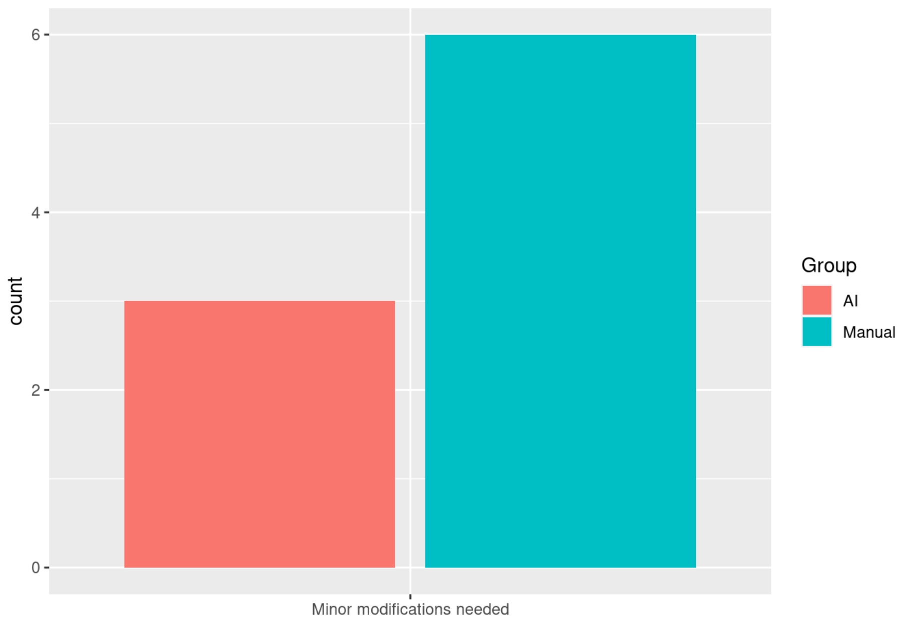

Too Jagged

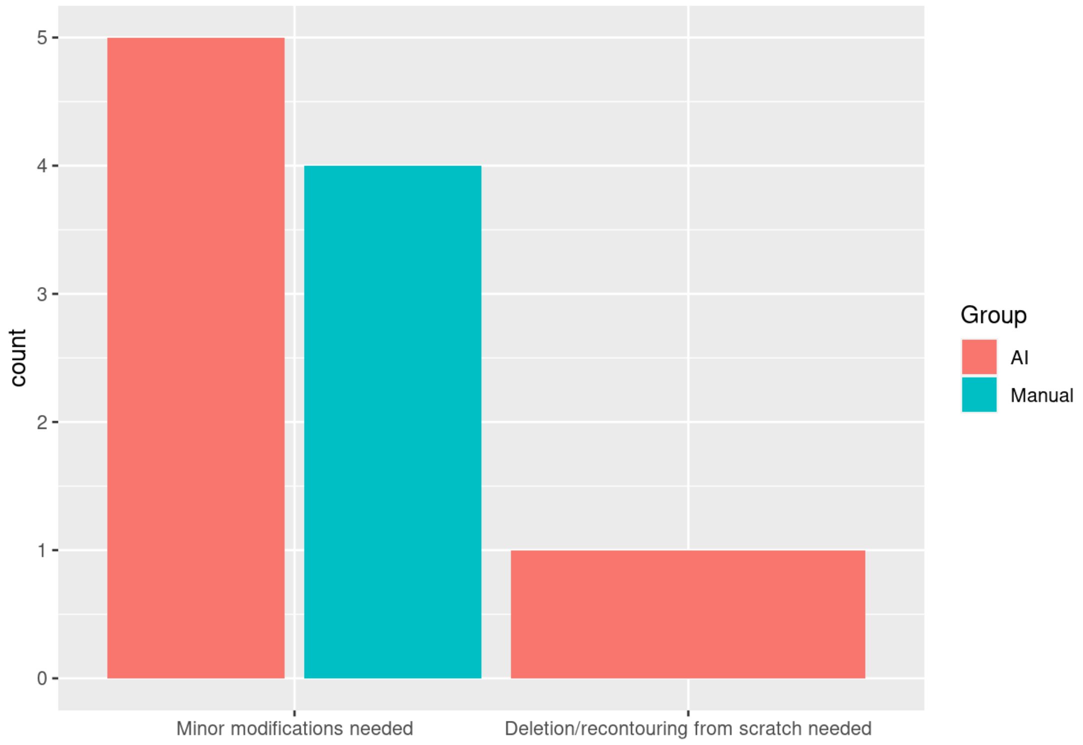

Ditzels or Islands

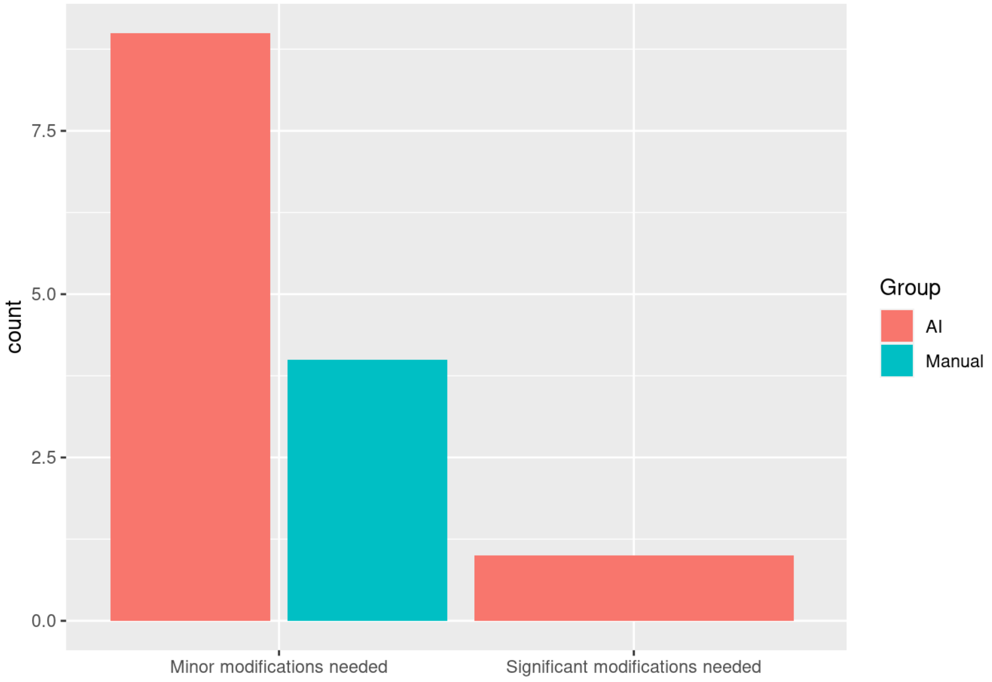

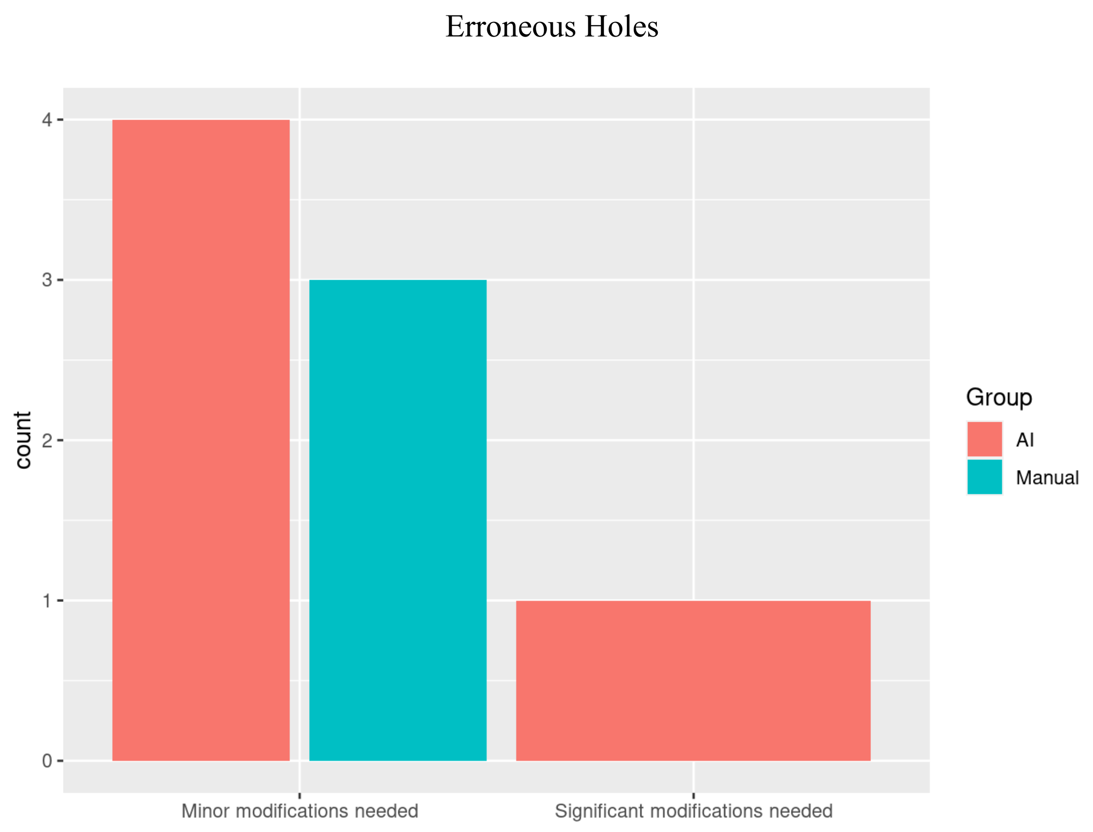
Erroneous Holes
count
4
3
2
1
0
Minor modifications needed
Significant modifications needed
Group
AI
Manual

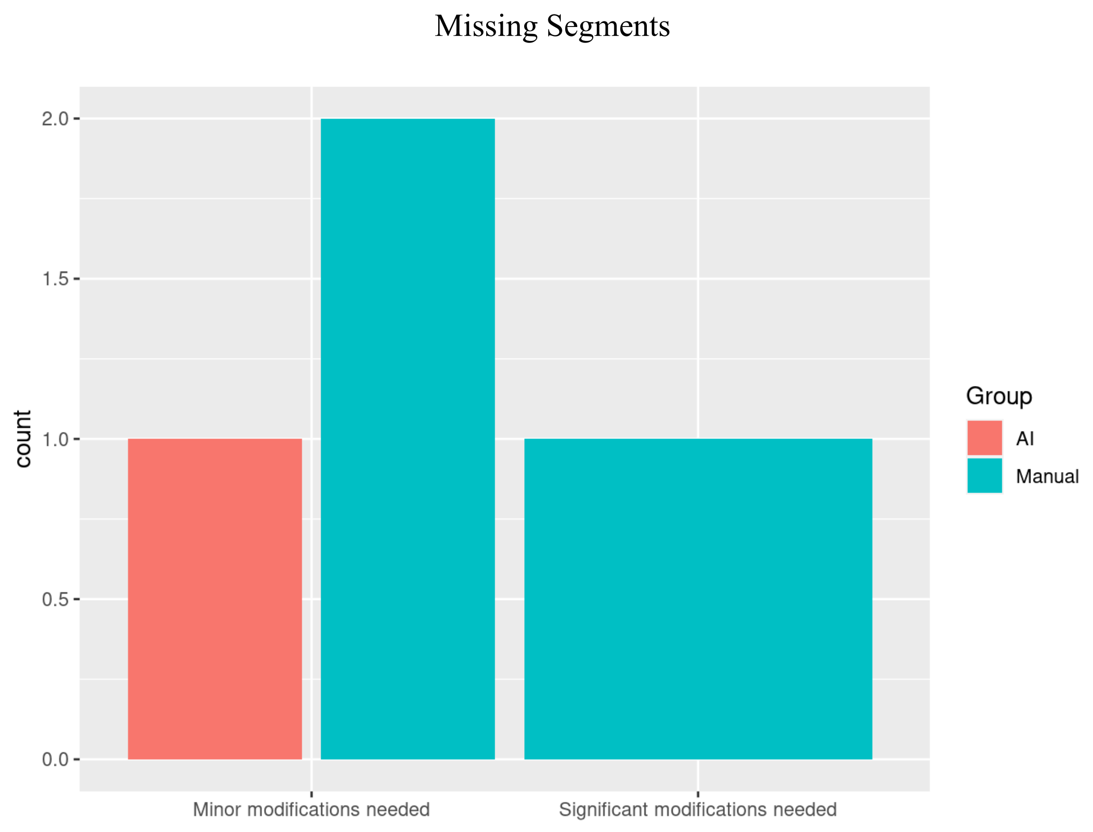
Missing Segments
count
2.0
1.5
1.0
0.5
0.0
Minor modifications needed
Significant modifications needed
Group
AI
Manual

Erroneously Overlapping Contours

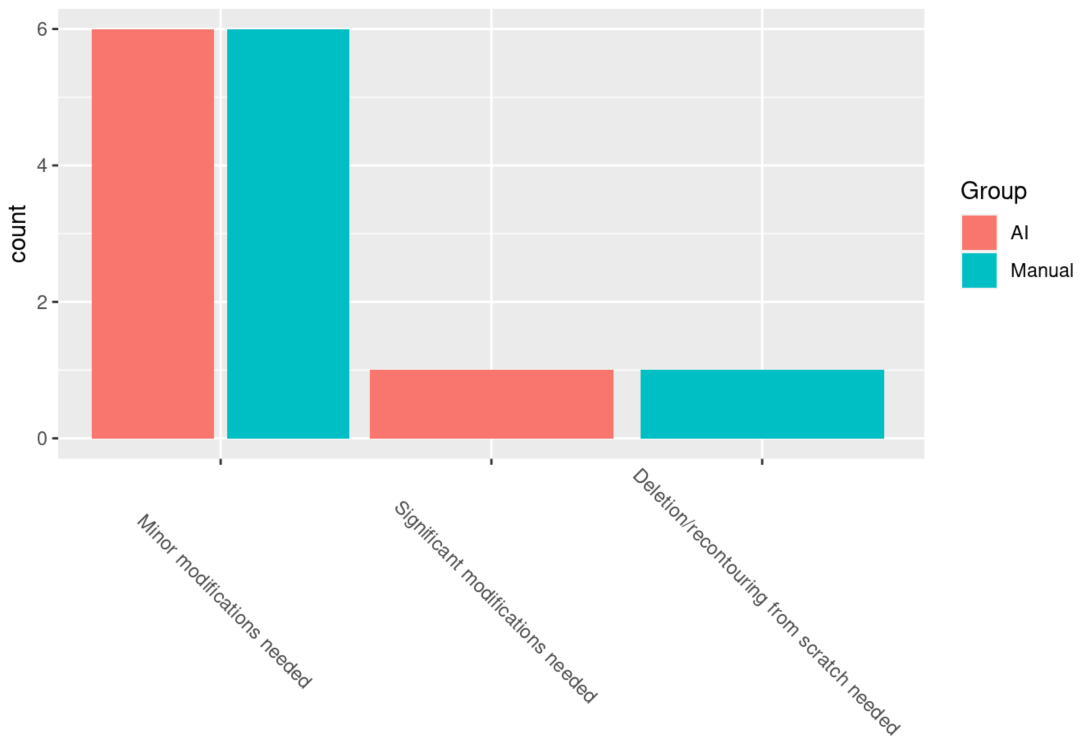

Connected structures have disconnected segments

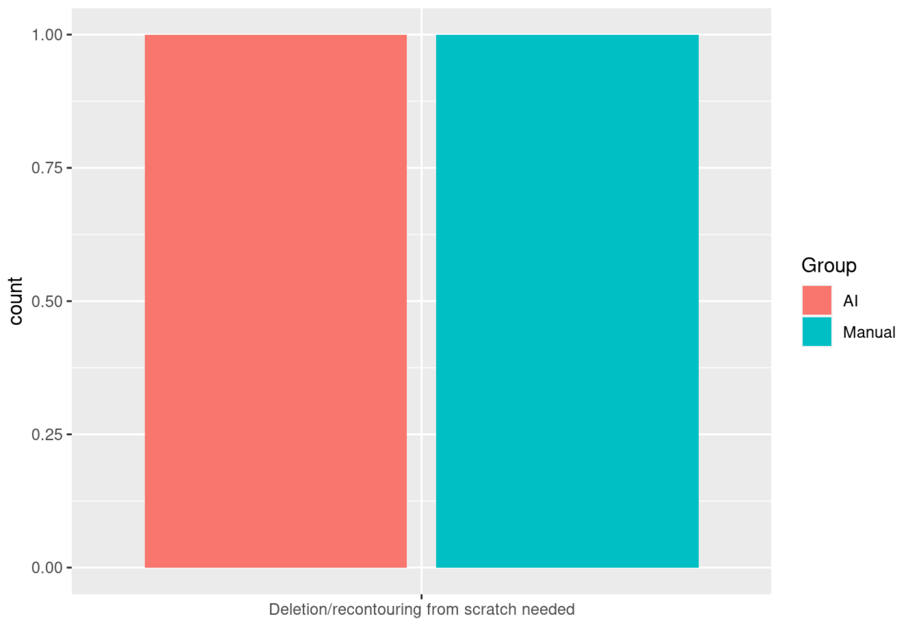

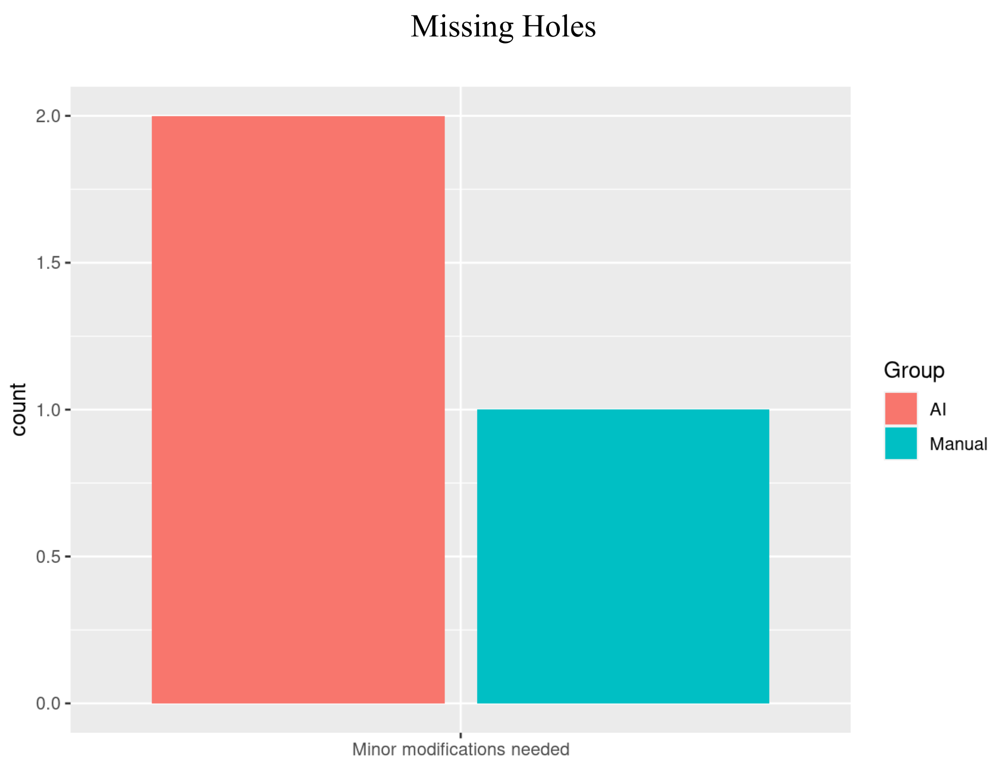

6.2 Organs Indicated as needing improvement

Selected as most severe organ error

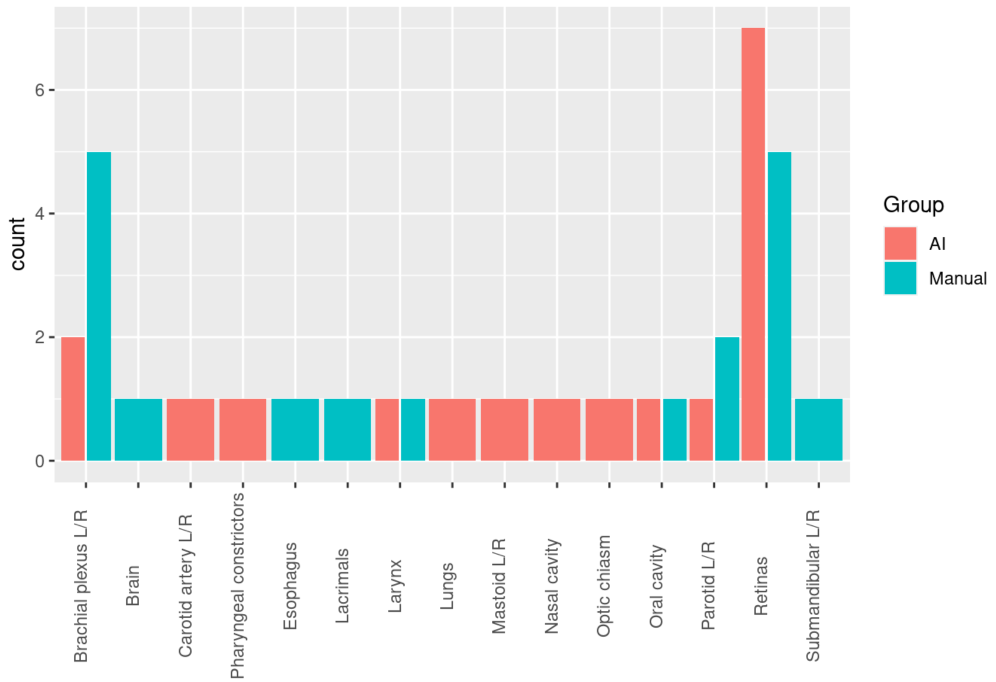

Selected as Most 2nd Most severe organ error

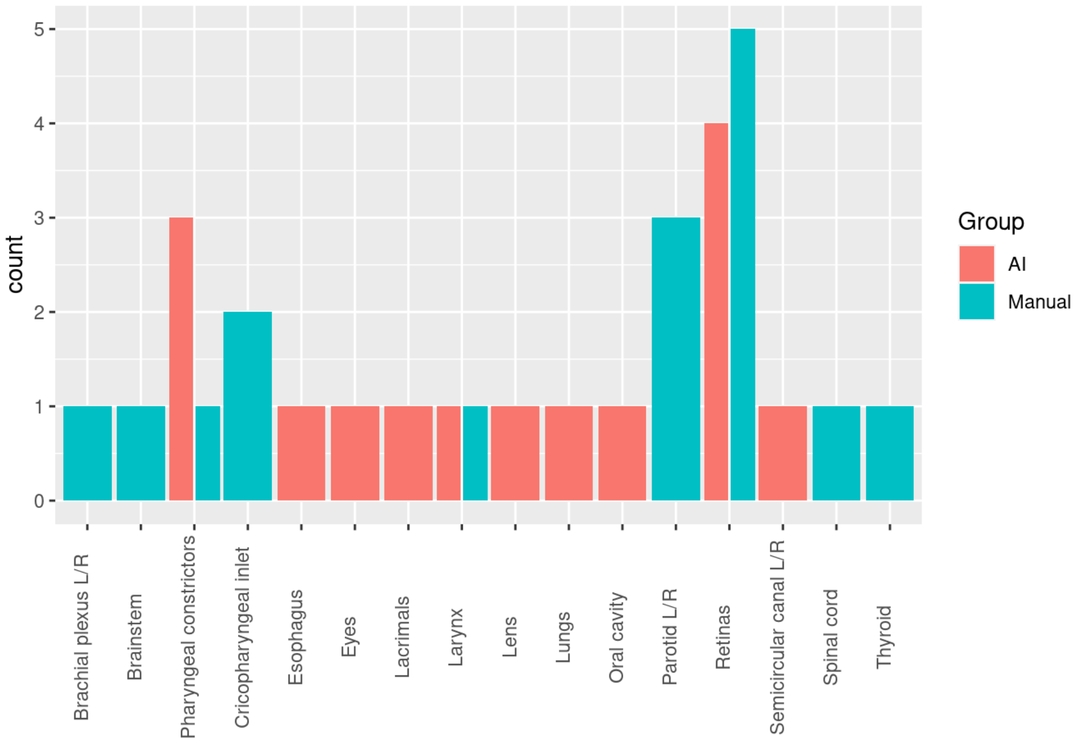

Selected as third most severe organ error

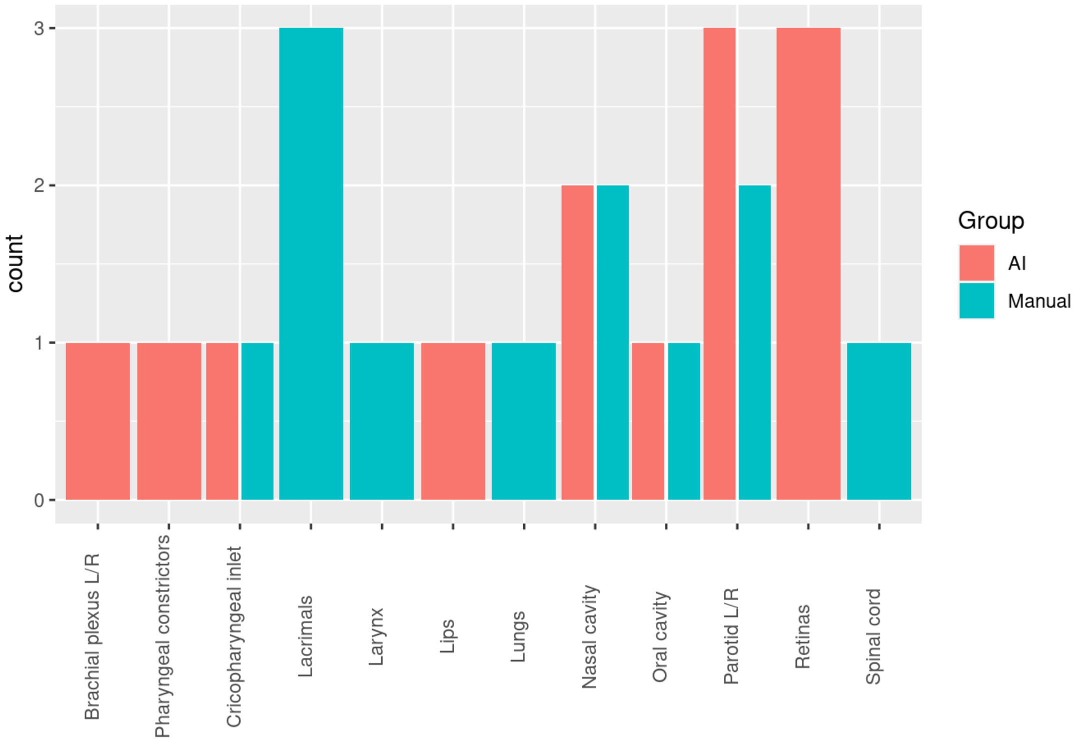

Overall Count of Selected Organs with errors

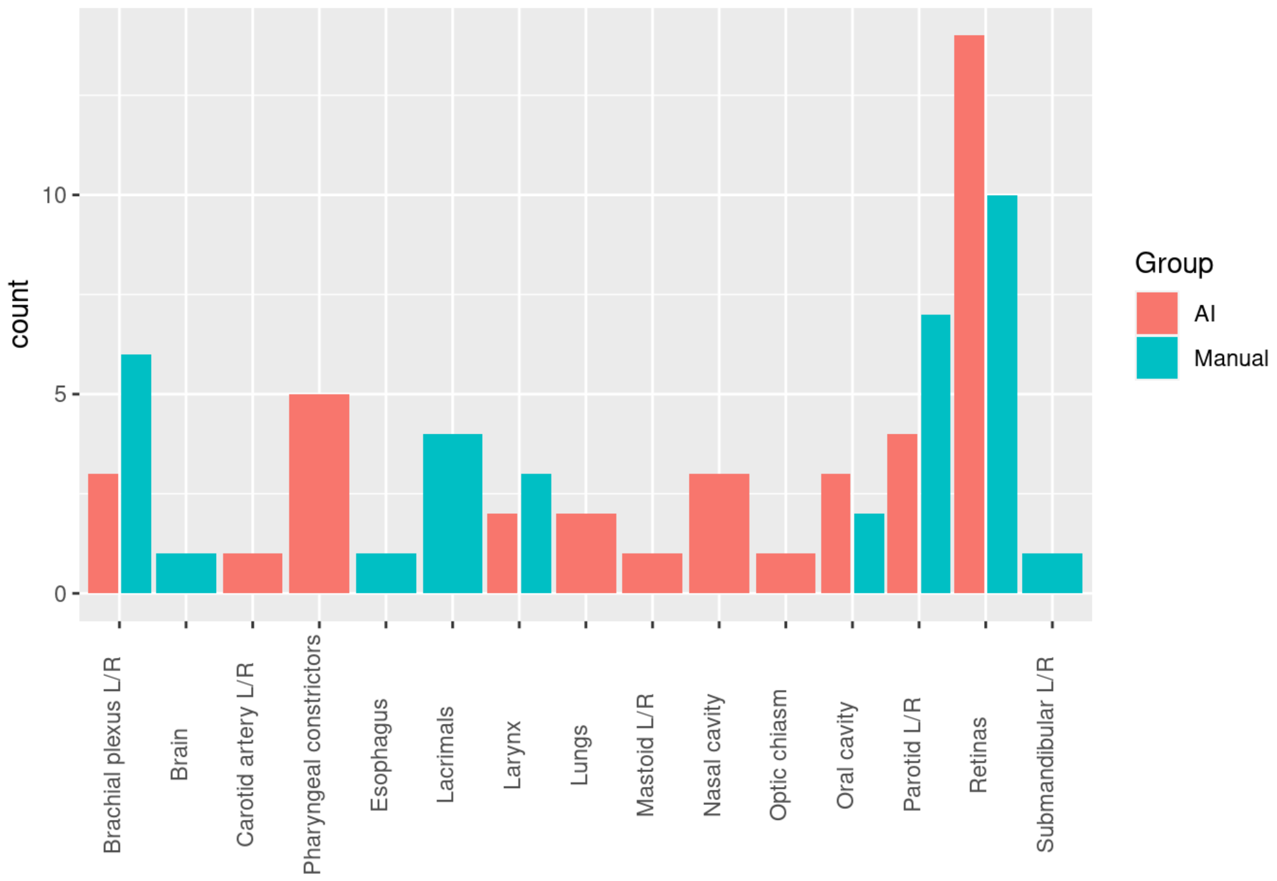

7 CONSORT-AI Checklist

Section	Item	CONSORT 2010 Item[a]		CONSORT-AI Item	Addressed on Page No[b]
			Title and Abstract		
**Title and Abstract**	1a	Identification as a randomised trial in the title	CONSORT-AI 1a,b Elaboration	(i) Indicate that the intervention involves artificial intelligence/machine learning in the title and/or abstract and specify the type of model.	1
	1b	Structured summary of trial design, methods, results, and conclusions (for specific guidance see CONSORT for abstracts)		(ii) State the intended use of the AI intervention within the trial in the title and/or abstract.	1
			Introduction		
**Background and objectives**	2a	Scientific background and explanation of rationale	CONSORT-AI 2a (i) Extension	Explain the intended use of the AI intervention in the context of the clinical pathway, including its purpose and its intended users (e.g. healthcare professionals, patients, public).	Abstract
	2b	Specific objectives or hypotheses			Introduction
			Methods		
**Trial design**	3a	Description of trial design (such as parallel, factorial) including allocation ratio			Methods, §2.5
	3b	Important changes to methods after trial commencement (such as eligibility criteria), with reasons			n/a
**Participants**	4a	Eligibility criteria for participants	CONSORT-AI 4a (i) Elaboration	State the inclusion and exclusion criteria at the level of participants.	Methods, §2.5

			CONSORT-AI 4a (ii) Extension	State the inclusion and exclusion criteria at the level of the input data.	Methods, §2.2
	4b	Settings and locations where the data were collected	CONSORT-AI 4b Extension	Describe how the AI intervention was integrated into the trial setting, including any onsite or offsite requirements.	n/a (retrospectiv e validation)
**Interventions**	5	The interventions for each group with sufficient details to allow replication, including how and when they were actually administered	CONSORT-AI 5 (i) Extension	State which version of the AI algorithm was used.	Methods, §2.5
			CONSORT-AI 5 (ii) Extension	Describe how the input data were acquired and selected for the AI intervention.	Methods, §2.2
			CONSORT-AI 5 (iii) Extension	Describe how poor quality or unavailable input data were assessed and handled.	Methods, §2.2
			CONSORT-AI 5 (iv) Extension.	Specify whether there was human-AI interaction in the handling of the input data, and what level of expertise was required of users.	Methods, §2.5
			CONSORT-AI 5 (v) Extension	Specify the output of the AI intervention	Methods, §2.4
			CONSORT-AI 5 (vi) Extension	Explain how the AI intervention's outputs contributed to decision-making or other elements of clinical practice.	Introduction, Methods, §2.5
**Outcomes**	6a	Completely defined pre-specified primary and secondary outcome measures, including how and when they were assessed			Ref 23, Results, Appendix D
	6b	Any changes to trial outcomes after the trial commenced, with reasons			n/a
**Sample size**	7a	How sample size was determined			Methods (§2.7)
	7b	When applicable, explanation of any interim analyses and			n/a

		stopping guidelines			
Randomisation					
**Sequence generation**	8a	Method used to generate the random allocation sequence			Methods, §2.5
	8b	Type of randomisation; details of any restriction (such as blocking and block size)			Methods, §2.5
**Allocation concealment mechanism**	9	Mechanism used to implement the random allocation sequence (such as sequentially numbered containers), describing any steps taken to conceal the sequence until interventions were assigned			Methods, §2.5
**Implementation**	10	Who generated the random allocation sequence, who enrolled participants, and who assigned participants to interventions			Methods, §2.5
**Blinding**	11a	If done, who was blinded after assignment to interventions (for example, participants, care providers, those assessing outcomes) and how			Methods, §2.5
	11b	If relevant, description of the similarity of interventions			n/a

**Statistical methods**	12a	Statistical methods used to compare groups for primary and secondary outcomes			Methods, §2.7
	12b	Methods for additional analyses, such as subgroup analyses and adjusted analyses			Methods, §2.5-2.7
Results					
**Participant flow** (a diagram is strongly recommended)	13a	For each group, the numbers of participants who were randomly assigned, received intended treatment, and were analysed for the primary outcome			Ref 23
	13b	For each group, losses and exclusions after randomisation, together with reasons			Results, §3.1
**Recruitment**	14a	Dates defining the periods of recruitment and follow-up			Methods, §2.5
	14b	Why the trial ended or was stopped			n/a
**Baseline data**	15	A table showing baseline demographic and clinical characteristics for each group			Table D.1
**Numbers analysed**	16	For each group, number of participants (denominator) included in each analysis and whether the analysis was by original			Results, §3.1

		assigned groups			
Outcomes and estimation	17a	For each primary and secondary outcome, results for each group, and the estimated effect size and its precision (such as 95% confidence interval)			Results
	17b	For binary outcomes, presentation of both absolute and relative effect sizes is recommended			n/a
Ancillary analyses	18	Results of any other analyses performed, including subgroup analyses and adjusted analyses, distinguishing pre-specified from exploratory			Appendix D
Harms	19	All important harms or unintended effects in each group (for specific guidance see CONSORT for harms)	CONSORT-AI 19 Extension	Describe results of any analysis of performance errors and how errors were identified, where applicable. If no such analysis was planned or done, explain why not.	Results, Appendix D
Discussion					
Limitations	20	Trial limitations, addressing sources of potential bias, imprecision, and, if relevant, multiplicity of analyses			Discussion
Generalisability	21	Generalisability (external validity,			Discussion

		applicability) of the trial findings			
**Interpretation**	22	Interpretation consistent with results, balancing benefits and harms, and considering other relevant evidence			Discussion
			Other Information		
**Registration**	23	Registration number and name of trial registry			IRB # 21-008372
**Protocol**	24	Where the full trial protocol can be accessed, if available			Ref 23
**Funding**	25	Sources of funding and other support (such as supply of drugs), role of funders	CONSORT-AI 25 Extension.	State whether and how the AI intervention and/or its code can be accessed, including any restrictions to access or re-use.	Internal

8 References – check formatting

1. Brouwer CL, Steenbakkers RJ, Bourhis J, Budach W, Grau C, Grégoire V, et al. CT-based delineation of organs at risk in the head and neck region: DAHANCA, EORTC, GORTEC, HKNPCSG, NCIC CTG, NCRI, NRG Oncology and TROG consensus guidelines. Radiother Oncol. 2015;117(1):83-90. https://doi.org/10.1016/j.radonc.2015.07.041.

2. Lu Y, Zhou JH, Guan C, editors. Minimizing Hybrid Dice Loss for Highly Imbalanced 3D Neuroimage Segmentation. 2020 42nd Annual International Conference of the IEEE Engineering in Medicine & Biology Society (EMBC); 2020 20-24 July 2020.

3. Shazeer N, Stern M. Adafactor: Adaptive Learning Rates with Sublinear Memory Cost. In: Jennifer D, Andreas K, editors. Proceedings of the 35th International Conference on Machine Learning; Proceedings of Machine Learning Research: PMLR; 2018. p. 4596--604.

4. Nelms BE, Robinson G, Markham J, Velasco K, Boyd S, Narayan S, et al. Variation in external beam treatment plan quality: An inter-institutional study of planners and planning systems. Pract Radiat Oncol. 2012;2(4):296-305. https://doi.org/10.1016/j.prro.2011.11.012.

5. Singh GK. Area Deprivation and Widening Inequalities in US Mortality, 1969–1998. Am J Public Health. 2003;93(7):1137-43. 10.2105/ajph.93.7.1137.

6. Nikolov S, Blackwell S, Zverovitch A, Mendes R, Livne M, De Fauw J, et al. Clinically Applicable Segmentation of Head and Neck Anatomy for Radiotherapy: Deep Learning Algorithm Development and Validation Study. J Med Internet Res. 2021;23(7):e26151. 10.2196/26151.

7. Brunenberg EJL, Steinseifer IK, van den Bosch S, Kaanders JHAM, Brouwer CL, Gooding MJ, et al. External validation of deep learning-based contouring of head and neck organs at risk. Physics Imag Radiat Oncol. 2020;15:8-15. https://doi.org/10.1016/j.phro.2020.06.006.

8. van der Veen J, Willems S, Deschuymer S, Robben D, Crijns W, Maes F, et al. Benefits of deep learning for delineation of organs at risk in head and neck cancer. Radiotherapy and Oncology. 2019;138:68-74. https://doi.org/10.1016/j.radonc.2019.05.010.

9. Zhong Y, Yang Y, Fang Y, Wang J, Hu W. A Preliminary Experience of Implementing Deep-Learning Based Auto-Segmentation in Head and Neck Cancer: A Study on Real-World Clinical Cases. Front Oncol. 2021;11:638197. 10.3389/fonc.2021.638197.

10. Fang Y, Wang J, Ou X, Ying H, Hu C, Zhang Z, et al. The impact of training sample size on deep learning-based organ auto-segmentation for head-and-neck patients. Phys Med Biol. 2021;66(18):185012.

11. Amjad A, Xu J, Thill D, Lawton C, Hall W, Awan MJ, et al. General and custom deep learning autosegmentation models for organs in head and neck, abdomen, and male pelvis. Med Phys. 2022;49(3):1686-700. https://doi.org/10.1002/mp.15507.

12. Thor M, Iyer A, Jiang J, Apte A, Veeraraghavan H, Allgood NB, et al. Deep learning auto-segmentation and automated treatment planning for trismus risk reduction in head and neck cancer radiotherapy. Phys Imag Radiat Oncol. 2021;19:96-101. https://doi.org/10.1016/j.phro.2021.07.009.

13. Wang W, Wang Q, Jia M, Wang Z, Yang C, Zhang D, et al. Deep Learning-Augmented Head and Neck Organs at Risk Segmentation From CT Volumes. Front Phys. 2021;9. 10.3389/fphy.2021.743190.
